# Microbial-based natural products as potential inhibitors targeting DNA gyrase B of *Mycobacterium tuberculosis*: an *in silico* study

**DOI:** 10.3389/fchem.2025.1524607

**Published:** 2025-01-23

**Authors:** Tilal Elsaman, Magdi Awadalla Mohamed, Malik Suliman Mohamed, Eyman Mohamed Eltayib, Abualgasim Elgaili Abdalla

**Affiliations:** ^1^ Department of Pharmaceutical Chemistry, College of Pharmacy, Jouf University, Sakaka, Saudi Arabia; ^2^ Department of Pharmaceutics, College of Pharmacy, Jouf University, Sakaka, Saudi Arabia; ^3^ Department of Clinical Laboratory Sciences, College of Applied Medical Sciences, Jouf University, Sakaka, Saudi Arabia

**Keywords:** *Mycobacterium tuberculosis*, DNA gyrase B, natural products, virtual screening, resistance

## Abstract

**Introduction:**

Since the emergence of *Mycobacterium tuberculosis* (MBT) strains resistant to most currently used anti-tubercular drugs, there has been an urgent need to develop efficient drugs capable of modulating new therapeutic targets. Mycobacterial DNA gyrase is an enzyme that plays a crucial role in the replication and transcription of DNA in MBT. Consequently, targeting this enzyme is of particular interest in developing new drugs for the treatment of drug-resistant tuberculosis, including multidrug-resistant tuberculosis (MDR-TB) and extensively drug-resistant tuberculosis (XDR-TB).

**Methods:**

In the present study, multiple computational tools were adopted to screen a microbial-based natural products database (NPAtlas) for potential inhibitors of the ATPase activity of MBT DNA gyrase.

**Results and discussion:**

Twelve hits were initially identified as the top candidates based on their docking scores (ranging from −9.491 to −10.77 kcal/mol) and binding free energies (−60.37 to −73.21 kcal/mol). Following this, computational filters, including ADME-T profiling and pharmacophore modeling, were applied to further refine the selection. As a result, three compounds 1-Hydroxy-D-788-7, Erythrin, and Pyrindolol K2 emerged as the most promising, exhibiting favorable drug-like properties. Notably, 1-Hydroxy-D-788-7, an anthracycline derivative, demonstrated superior binding affinity in molecular dynamics simulations. The RMSD values, ranging from 1.7 to 2.5 Å, alongside RMSF analysis and a detailed evaluation of the established interaction forces, revealed that 1-Hydroxy-D-788-7 was the strongest binder to Mycobacterial DNA Gyrase B. The stable binding and favorable interaction profile highlighted 1-Hydroxy-D-788-7 as a top hit. These comprehensive computational findings strongly support the potential of 1-Hydroxy-D-788-7 as an effective anti-TB lead compound, warranting further experimental validation to confirm its therapeutic efficacy.

## 1 Introduction

**FIGURE 1:**
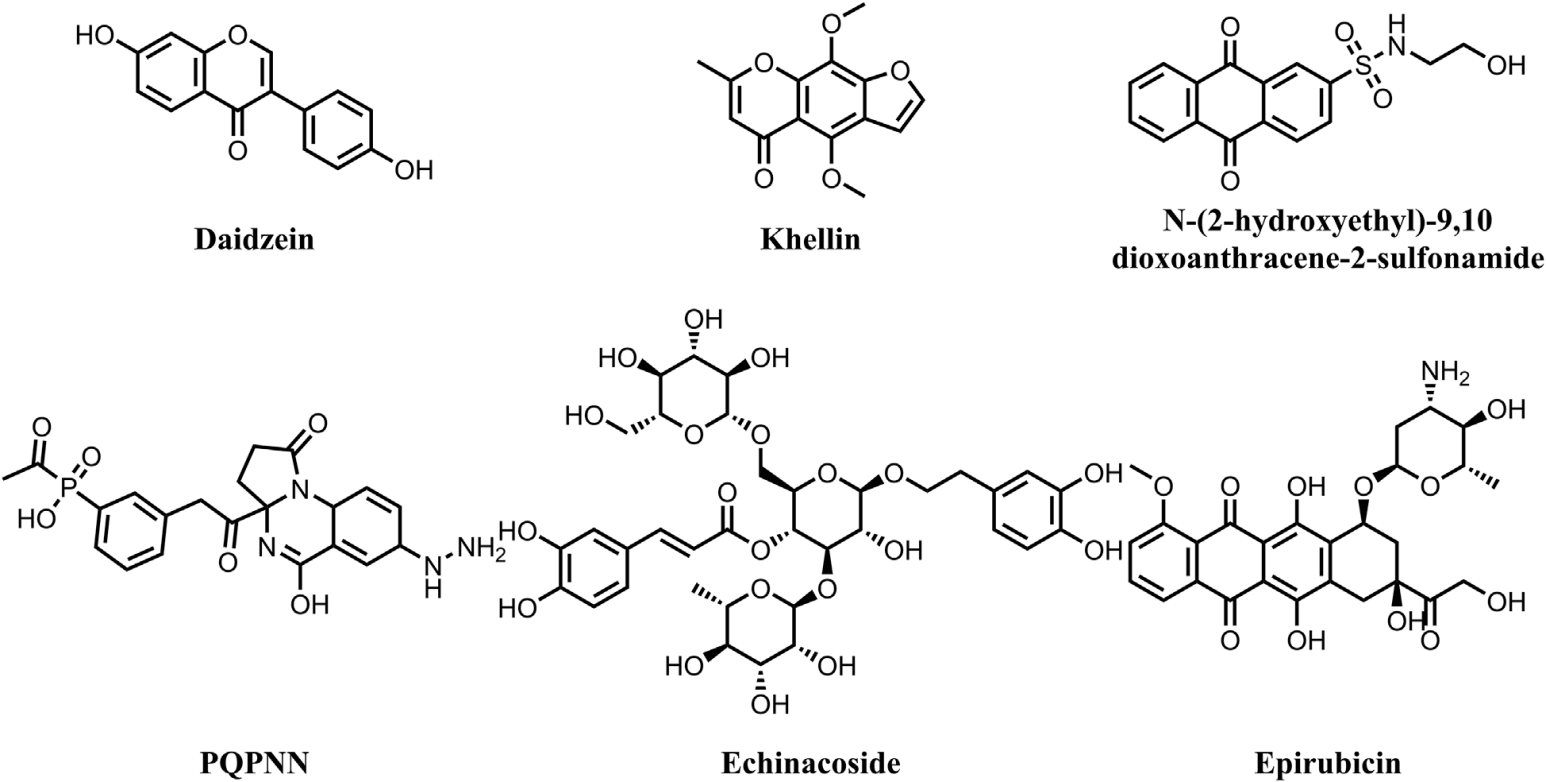
Chemical structures of natural products demonstrating potent inhibitory activity against mycobacterial DNA gyrase.

*Mycobacterium tuberculosis* (MBT) is the etiological agent of tuberculosis (TB) and affects one-third of world’s population latently. Tuberculosis continues to be a longstanding global health problem, with 10.6 million new cases diagnosed and 1.6 million deaths attributed to TB worldwide in 2021 (Bagcchi, 2023). The recent surge in morbidity and mortality rates of tuberculosis is linked to the consistent rise of mycobacterial strains that are resistant to most of the currently used anti-TB medications (Seung et al., 2015; Miotto et al., 2018; Allue-Guardia et al., 2021). The global effort to end TB faces significant obstacles due to the increasing incidence of multidrug-resistant (MDR), extensively drug-resistant (XDR), and extremely drug-resistant (XXDR) TB (Dheda et al., 2017). Furthermore, the emergence of totally drug-resistant TB (TDR-TB) strains has severely compromised treatment options (Verma et al., 2021). Reports indicate that 75% of TB patients infected with XDR-MBT strains do not respond to the current anti-TB drugs (Dookie et al., 2018). Therefore, discovery and development of new effective anti-tuberculous therapies is urgently needed to replace the current medications, as the microorganism has developed multiple resistance mechanisms. DNA gyrase is an ATP-dependent enzyme that controls the transcription, replication and recombination processes by introducing transient breaks in both DNA strands. The DNA gyrase of MBT consists of four subunits, including two alpha (2α) and two beta (2β) subunits (Qiu et al., 2024). The α-subunits (gyrase A) are responsible for introducing negative supercoil (Germe et al., 2024), a process that requires energy from ATP hydrolysis by the β subunits (gyrase B) (Kashyap et al., 2018). Mycobacterial DNA gyrase A is the direct target for the fluoroquinolones, which are used as part of second-line treatment in combination with injectable drugs such as Amikacin, Capreomycin, and Streptomycin for treatment of MDR-TB (Miotto et al., 2018). They have proven effective against MBT and are considered the first anti-TB drugs developed since rifampin and are now a key part of treatment protocols for rifampin-resistant TB (Aubry et al., 2004). Studies have demonstrated that MBT’s resistances to fluoroquinolone primarily results due to mutations in gyrase A rather than gyrase B (Avalos et al., 2015; Kashyap et al., 2018). The surge in resistance to fluoroquinolones has gathered the interest in focusing on the GyrB subunit. Targeting this subunit could offer similar phenotypic impacts on bacterial survival as fluoroquinolones, thereby extending the utility of DNA gyrase as a therapeutic target for tuberculosis (Chopra et al., 2012). In this context, the objective of this study was to investigate potential inhibitors of DNA gyrase B in MBT from natural products, with a particular focus on addressing the challenges posed by drug-resistant strains. The study aimed to enhance understanding of how natural compounds interact with DNA gyrase B, potentially paving the way for the development of new therapies targeting drug-resistant tuberculosis. For many years natural products have long been known as a valuable source of anti-TB drugs (Sudarshan et al., 2024). Many of the currently used anti-TB drugs, such as Streptomycin, Kanamycin, Amikacin, Rifampicin, Cycloserin, Capreomycin, originate from natural sources. Nonetheless, no natural product-derived anti-TB drug has advanced to the market since the 1970s (Han et al., 2022). This could be ascribed to several challenges, including inefficient isolation, difficulties in chemical structures verification, and limited knowledge about potential targets (Atanasov et al., 2021; Han et al., 2022). Fortunately, recent technological advancements have overcome these obstacles, comprising the improved isolation and structure verification tools, microbial platforms for production enhancement, and modern methods for identifying and validating the targets of natural bioactive molecules (Atanasov et al., 2021; Thuan et al., 2022; Zhu et al., 2022). Consequently, natural products have once again received a great attention from the scientific community, particularly for addressing the problem of microbial drug resistance. Multiple studies revealed the potential of natural products for inhibiting the ATPase activity of mycobacterial DNA gyrase (Figure 1). Jagatab et al. reported the potent DNA gyrase inhibitory activity of daidzein (isofalvonoid) and khelline (furanochromone) with IC_50_ values of 0.042 and 0.822 μg/mL, respectively (Jagatap et al., 2023). In addition, Amorim et al. reported the potential of Anthraquinones, a class of naturally occurring organic compounds with a characteristic three-ring structure. Computational investigations identified 7122772 (N-(2-hydroxyethyl)-9,10 dioxoanthracene-2-sulfonamide) as the best-scored ligand (Amorim et al., 2022). Moreover, an independent study involved virtual screening of a dataset of 20,098 natural products reveled compound PQPNN as the best in terms of binding affinity towards mycobacterial DNA gyrase B (Arevalo and Amorim, 2022). Furthermore, Structure-based drug repurposing study identified Echinacoside and Epirubicin as potent inhibitors of the catalytic activity of mycobacterial DNA gyrase with IC_50_ values of 2.1–4.7 µM (Balasubramani et al., 2020).

Computer-aided drug design approach gain popularity in modern drug discovery and development field, guiding and expediting various phases of the drug design process. These approaches can be classified into (i) Structure based drug design (SBDD) and (ii) Ligand based drug design (LBDD) (Yu and MacKerell, 2017; Chang et al., 2022). The SBDD approach is used to predict the positioning of a small molecule into a target protein active site and to accurately estimate its binding free energetics (Wang et al., 2018). SBDD is regarded as a faster and more cost-effective approach for hit identification and optimization compared to conventional drug discovery approaches (Batool et al., 2019). SBDD comprises numerous methods, such as molecular docking, molecular dynamics (MD) simulations and *de novo* drug design (Chang et al., 2022). In this context, the aim of this study was to explore the potential of microbial-based natural products as inhibitors of DNA gyrase B in *M. tuberculosis* using various computational approaches. This study is the first to screen a library of natural products from microbial origins, which are known for their potent chemotherapeutic properties, with many of the most effective currently used antimycobacterial drugs having originated from microbes. In addition, multiple computational tools were efficiently integrated to extensively analyze and interpret the binding modes, affinities and interactions of the top hits, enhancing the reliability of our findings. To this end, in the present study, structure-based virtual screening was applied using ‘Virtual Work Flow’ to identify potential ATPase inhibitors from the microbial-based Natural Products Atlas (NPAtlas) database (https://www.npatlas.org). The MM-GBSA (molecular mechanics/Generalized-Boltzmann surface area) calculation was performed to estimate the binding free energetics of the enzyme-ligand complexes. Following this, the identified potential inhibitors were filtered through *in silico* physicochemical and pharmacokinetic analyses. In addition, molecular dynamics (MD) simulation was carried out to assess the binding stability of the potential hits with the enzyme binding pocket.

## 2 Materials and methods

In this study, softwares implanted in Schrödinger suite were used including, Protein Preparation Wizard (Madhavi Sastry et al., 2013), LigPrep (Schrödinger, LLC, 2023a), Glide (Friesner et al., 2004; Halgren et al., 2004; Friesner et al., 2006), Prime (Jacobson et al., 2004; Klyshko et al., 2024), QikProp (Divyashri et al., 2020), Phase (Dixon et al., 2006a; Dixon et al., 2006b), Jaguar (Bochevarov et al., 2013) and Desmond (Ivanczi et al., 2023). Maestro graphical interface (Schrödinger, LLC, 2023b) was used to access these softwares. Additionally, ADMETlab 2.0 (Xiong et al., 2021), an integrated free web-based platform was employed in ADMET properties predictions.

### 2.1 Target selection and validation

The protein reliability report was generated using the PDB structures (**4B6C**, **4BAE**, **3ZKB**, and **3ZM7**) and their corresponding reflection data. The structures were refined using the Protein Preparation Wizard in Schrödinger, and the reliability of the models was assessed through both pre- and post-refinement reports. During the refinement process, missing atoms, steric clashes, and unsatisfied buried donors were manually addressed using PyMOL and Swiss PDB Viewer (SPDBV) (Guex et al., 2009). The best crystal structure was selected based on the overall quality of the refinement, the results of the reliability assessments, and the stability of the model, ensuring that it would provide an accurate basis for subsequent analysis.

### 2.2 Receptor based virtual screening

Virtual screening analysis of the microbial-based Natural Products Atlas (NPAtlas) database was carried out using Virtual screening workflow of Schrödinger software suite. This database was selected to perform the screening based on several factors including: (i) its relevancy to our study aimed at identifying microbial-derived natural products which are known for their potential in developing new medicines for treating infectious diseases (Almansour et al., 2023). In addition, it comprehensively covers microbial-derived natural products comprising curated data on microbial metabolites, making it a valuable resource for identifying bioactive compounds. Furthermore, its user-friendly web interface and updated repository of microbial metabolites were key factors in our selection. For docking studies the downloaded compounds library in 2D SDF formats were converted to 3D formats and then were optimized using OPLS4 force field. The LigPrep module of Schrödinger was used to prepare the structures for docking, providing that the original chirality of the ligands were maintained. The SDF files, containing 2D structures of the investigated library, were imported into Maestro and processed using the LigPrep module. This module converted the 2D structures into 3D formats, optimizing their geometry, generating appropriate ionization states, and ensuring stereochemical accuracy. The resulting output was saved in the MAE (Maestro) file format. The possible ionization states were generated at pH 7.00 ± 2 units using Epik, and we obtained one low energy conformer for each ligand. In the present work, the 3D crystal structure of the target MBT DNA Gyrase B (PDB code: 4B6C) with co-crystal ligand was downloaded from the protein data bank website (http://www.rcsb.org) (Agrawal et al., 2013). Subsequently, the multi-step Protein Preparation Wizard (PrepWizard) from Maestro Task was employed to prepare the target protein structure. All water molecules beyond 5 Å were removed and the co-crystal ligand was kept in the binding site. Furthermore, OPLS4 force field was employed for optimization and energy minimization. Following this, receptor grid generation tool embedded within the Schrödinger suite was utilized for the 3D cubic grid box generation around the co-crystal ligand. Molecular docking was performed with constraints applied to the critical residue ASP79 (Supplementary Figure S1) to ensure accurate and focused interaction analysis. This approach emphasized the role of ASP79 in stabilizing ligand binding and allowed for the identification of compounds with strong and specific interactions at this key site. Next, Glide program was used to screen the prepared ligands against the refined target protein following multistep screening approach. This approach is an advanced technique that refines the docking process, enhancing both accuracy and reliability, which results in more precise predictions of ligand binding (Owoloye et al., 2022). It is designed to efficiently run a full sequence of tasks that facilitate the screening of large compound libraries. These libraries are tested against one or more target proteins to identify potential inhibitors. This process helps in rapidly narrowing down promising hits for further analysis and testing (Perez-Regidor et al., 2022). Several studies in the literature have used multistep molecular docking to identify inhibitors from large compound libraries targeting MBT (Mustyala et al., 2015; Naz et al., 2019; Kumar et al., 2023). Initially, Glide high-throughput virtual screening (HTVS) mode was utilized for filtering the compounds library, then for further screening the standard precision (SP) mode was used and finally more accurate docking calculations results were obtained using extra precision (XP) mode. For each input molecule, one best pose was generated, and the molecules were ranked based on their Glide XP docking score. Additionally, enrichment calculations were performed to validate the docking results and evaluate the effectiveness of the virtual screening process in distinguishing between active compounds and decoys for the potential inhibition of MBT GyrB. The enrichment process assessed the screening workflow’s ability to prioritize active compounds from a dataset of 1,466 ligands. Key metrics, including EF, AUC, BEDROC, and ROC curve analysis, were calculated, resulting in the identification of 18 active compounds. These findings demonstrated the reliability and robustness of the virtual screening methodology in identifying potential GyrB inhibitors.

### 2.3 MM-GBSA calculations

Estimation of the binding free energies of receptors and docked ligands was done using Prime module interfaced with Schrödinger. Post docking generated Pose Viewer Files (PVF) of the top hits were used as input files for energy computation for each hit. VSGB 2.0 solvation model and OPLS4 force field were used to calculate the binding free energy descriptors following the protocol that was previously reported in the literature (Azam et al., 2021). MMGBSA ∆G binding free energy score was employed to rank the ligands.

### 2.4 *In silico* ADMET profiling

In this study, the computational tool QikProp (Schrodinger Release 2023-1) was used to evaluate the ADMET profiles and drug-likeness descriptors of the top hits with high binding free energy scores along with the reference drug candidate SPR720. Ligand structures were prepared using the LigPrep tool to optimize 3D geometries and assign protonation states at a physiological pH of 7.4. Predictions were generated using the default settings of QikProp, which include evaluations of key parameters such as LogP, solubility (LogS), CNS permeability, human oral absorption, and toxicological risks. All calculations were conducted in standalone mode within the Schrödinger software environment. The program offers a set of recommended ranges for various properties and descriptors of small molecules, based on an analysis of 95% of known drugs. The results were exported in an MS Excel file, displaying the number of principal descriptors and ADME predictions in MS Excel file format. Additional toxicity parameters were estimated using the web-based tool ADMETlab 2.0 freely available at (https://admetmesh.scbdd.com). The molecules were drawn in Schrödinger, and its canonical SMILES representation was then submitted to the ADMETlab 2.0 webserver for evaluation using default parameters. All of the calculated parameters were evaluated for compliance with their respective specified limits.

### 2.5 Pharmacophore modeling

Pharmacophore modeling was carried out using the Phase module implanted in Schrödinger suite. The ligands obtained from the literature (67 compounds) were considered active when the IC_50_ for the inhibition of MBT DNA Gyrase B was <20 µM. These ligands were prepared via retention of the original chirality, generation of the ionization states at pH 7.00 ± 2 using OPLS4 force fields. Fifty conformers were generated for each active ligand and the hypothesis requirement was set to match 50% of the active ligands. The pharmacophore model was developed based on the six chemical features, namely, hydrogen bond donor (D), hydrogen bond acceptor (A), hydrophobic group (H), aromatic ring (R), negative ionizable group (N), and positive ionizable group (P). The resultant hypotheses were ranked according to their phaseHypoScores. Following the generation of the hypotheses, the top hits were screened against the high-ranking hypothesis and they were required to match all four pharmacophore features of the model to be selected for MD simulations study.

### 2.6 Quantum computational calculations

DFT method in the Jaguar module of Schrödinger suite was used to perform quantum chemical calculations of the top three hits 1-Hydroxy-D-788-7, Erythrin, and Pyrindolol K2 electronic molecular properties such as Electron density, MESP and energies of both HOMO and LUMO molecular orbitals (Khan and Singh, 2023). These energies were then utilized to compute various quantum chemical parameters, including the energy gap (HLG), chemical hardness, chemical softness, electronegativity, and the global electrophilicity index, following equations reported in the literature (Srivastava and Misra, 2021; Guezane-Lakoud et al., 2023). The electron-deficient surfaces are marked by the blue color, whereas the electron-rich ones are indicated by the red color.

### 2.7 MD simulations

MD simulation study was performed using Desmond for the best ligand-target complexes of the co-crystal ligand and the top three hits 1-Hydroxy-D-788-7, Erythrin, and Pyrindolol K2 taken from the docking experiments. In the first step, SPC solvation model was employed and the system was placed within orthorhombic water box of 10 Å × 10 Å × 10 Å coordinates to ensure complete coverage of the surface of each complex with solvent model (Alghamdi et al., 2023). Next, Na^+^ counter ions were added to the built systems to balance the net charges, and 0.15 M NaCl was further included to neutralize the systems. Finally, minimization and pre-equilibration of these systems were done before the simulation run using the default relaxation protocol. The system’s temperature was fixed at 300 K, and the atmospheric pressure at 1 bar, using the isothermal-isobaric (NPT) ensemble (Lazim et al., 2020). A 100 ns simulation was carried out to strike a balance between computational cost and the ability to observe important dynamic behaviors. Data from 1,000 frames were generated, with a recording interval of 100 ps. The average RMSD and RMSF were calculated for each protein region as well as for the entire protein. This time frame effectively captures key stability metrics such as RMSD, hydrogen bond stability, the maintenance of critical interactions, and the overall structural integrity of the system (De Vivo et al., 2016). Further, multiple studies have used MD simulations lasting 50–200 ns to investigate protein-ligand interactions with MBT targets, including DNA gyrase B (Amorim et al., 2022; Arevalo and Amorim, 2022; Pakamwong et al., 2024).

## 3 Results and discussion

**FIGURE 2:**
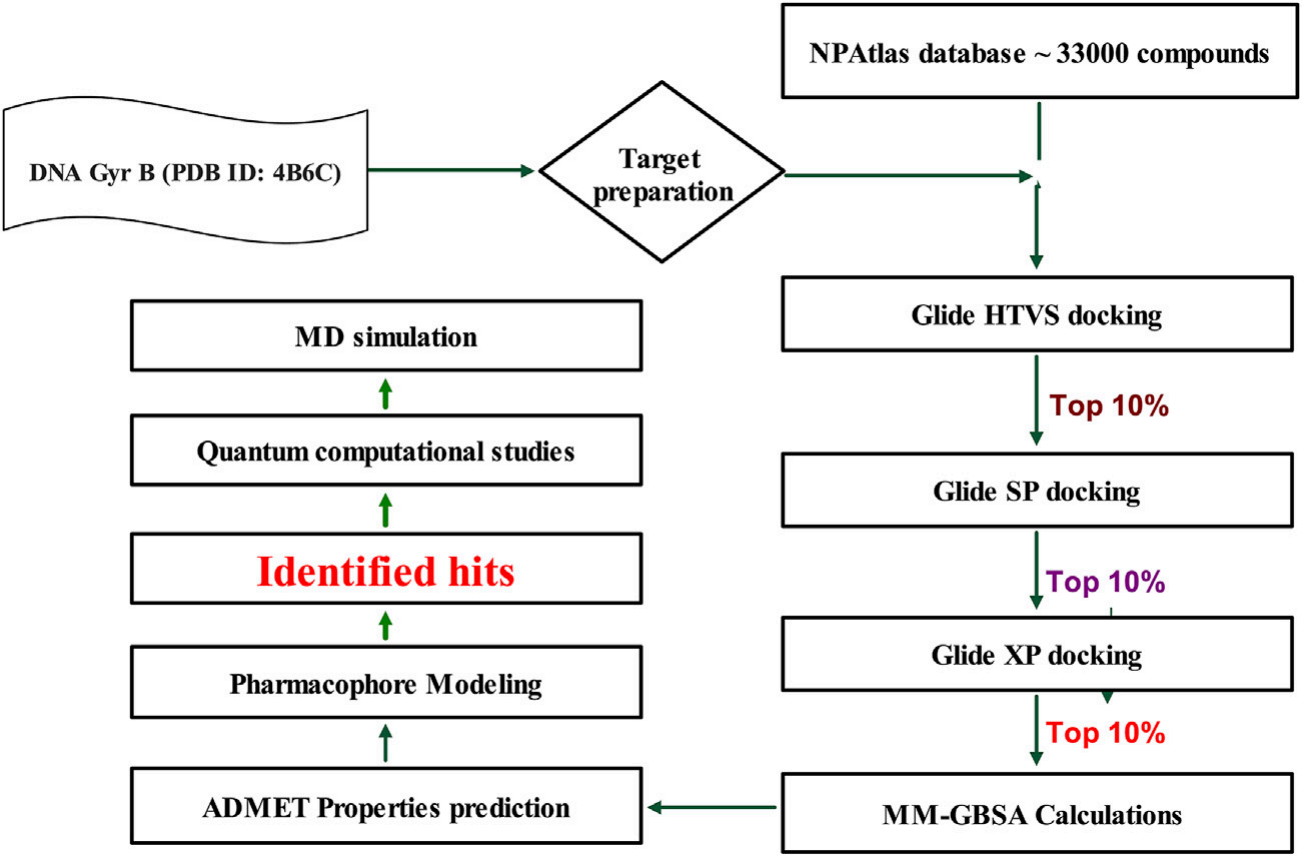
Work Flow for Virtual Screening. Generated using Microsoft PowerPoint.

Mycobacterial DNA gyrase is crucial for processes such as DNA replication, transcription, and recombination (Aubry et al., 2004). Fluoroquinolones act on GyrA, a component of the functional gyrase complex. Resistance to fluoroquinolones commonly arises from point mutations in the *gyrA* gene, leading to class-wide resistance against fluoroquinolones. In contrast, targeting GyrB produces similar effects on bacterial viability, preserving the potential of DNA gyrase as a viable drug target (Aubry et al., 2004). In this context, a library of microbial-based natural products (33000 compounds) was virtually screened to identify potential inhibitors of DNA Gyrase B of MBT, following the work flow illustrated in Figure 2.

### 3.1 Target selection and validation

Choosing the appropriate protein structure is a crucial step in ensuring the success of drug discovery efforts that rely on structure-based design approaches. Since multiple crystal structures for the same protein are often available in the Protein Data Bank (PDB), selecting the most suitable structure is essential for achieving accurate and reliable results (Murumkar et al., 2023). The protein structures were refined using the Protein Preparation Wizard, and their reliability was assessed through reports generated before and after refinement (Supplementary Figure S2) using the protein reliability report in Schrödinger. Among the analyzed structures, **4B6C** (Shirude et al., 2013) from *Mycobacterium smegmatis* emerged as the most reliable due to its high resolution, relevance to the target, and superior experimental validation. In comparison, **4BAE** showed minor deviations and improvements, while **3ZKB** and **3ZM7** exhibited persistent issues, including low resolution and structural instability, reducing their suitability for accurate modeling. Based on these qualities, **4B6C** was selected for virtual screening of 33,000 microbial-based products, while the other structures require further optimization.

### 3.2 Receptor based virtual screening

**FIGURE 3:**
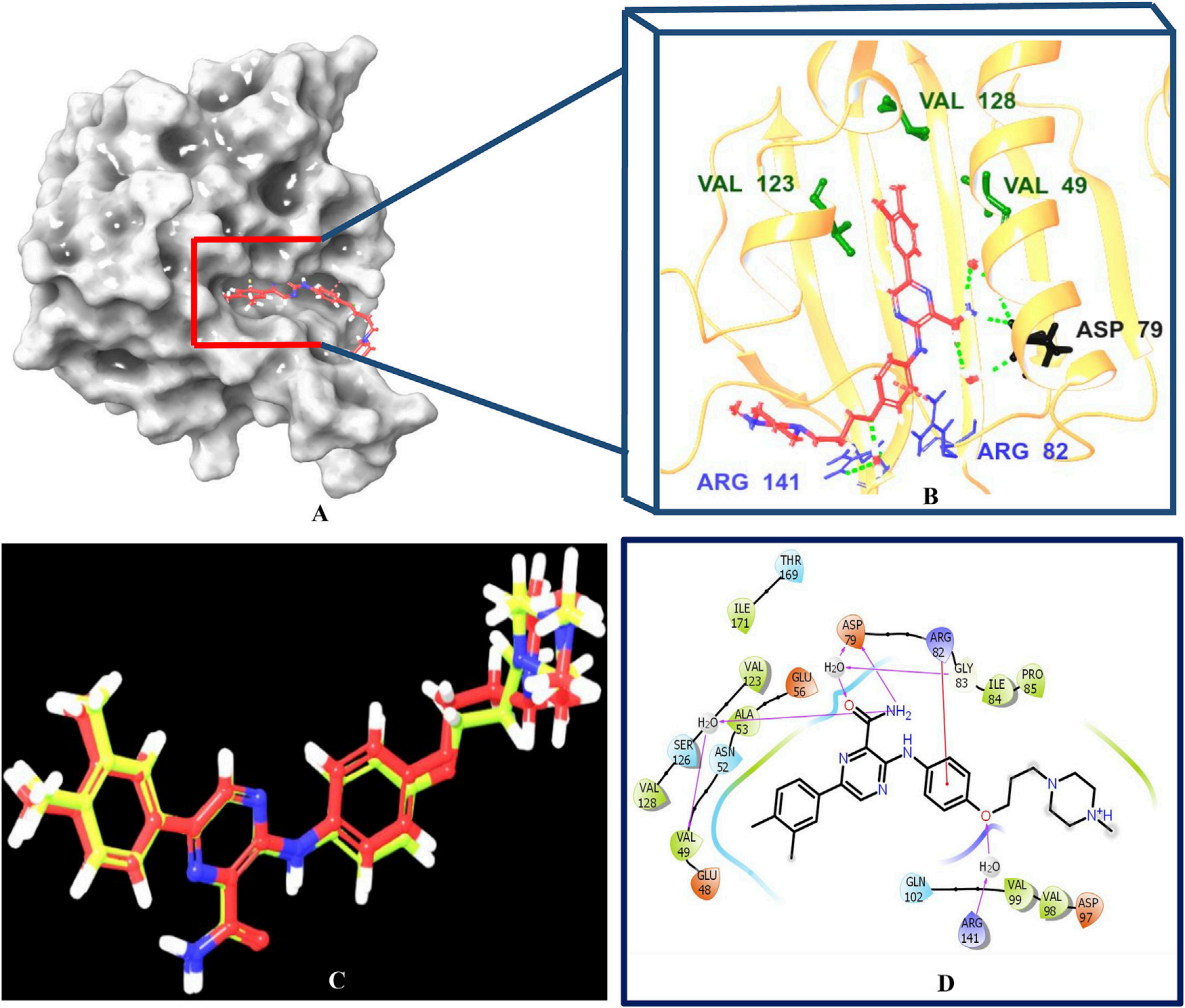
Post-docking analysis of the co-crystal ligand with the ATP-binding site of the GyrB subunit of MBT DNA gyrase. **(A, B)** representing 3D crystal structure of the enzyme bound to the co-crystal ligand. **(C)** represents the superposition of the docked ligand pose (yellow) into the original ligand pose (red) (RMSD < 2 Å. **(D)** represents 2D enzyme-ligand interaction of the co-crystal ligand. The interacting residues of the binding site are VAL49, ASP79, ARG82, VAL123, VAL128, ARG141 are represented by three-letter codes. In **(B)** the HB and the Pi‒cation interactions are shown by green and red dotted lines, respectively. In **(D)**, the HB and Pi-cation interactions are shown by magenta, and red lines, respectively. Created using Maestro interface of Schrödinger suite version 2023-1.

**FIGURE 4:**
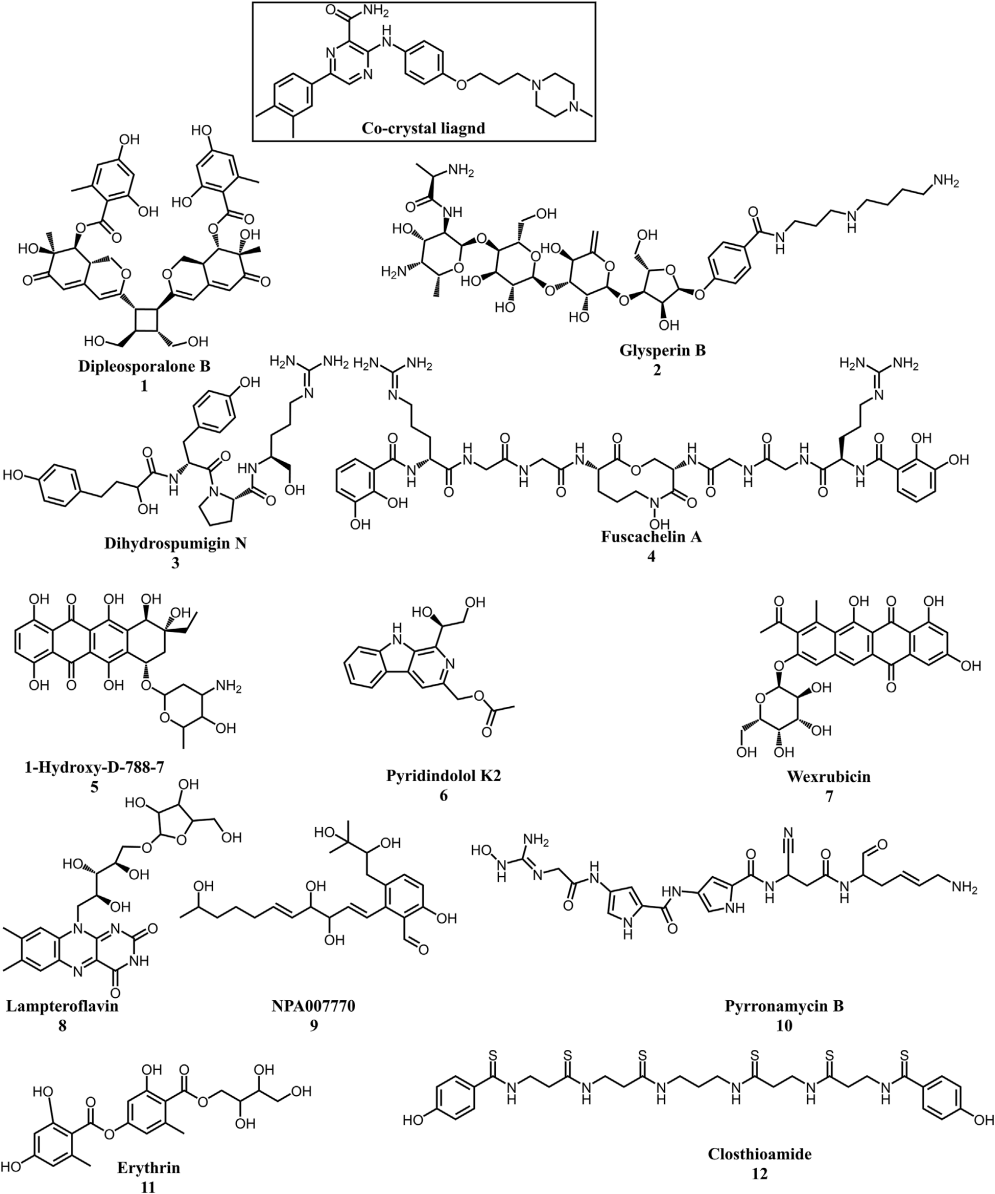
Chemical structures of the top 12 hits and the co-crystal ligand. Created using ChemDraw office version 20.1.1.

**FIGURE 5:**
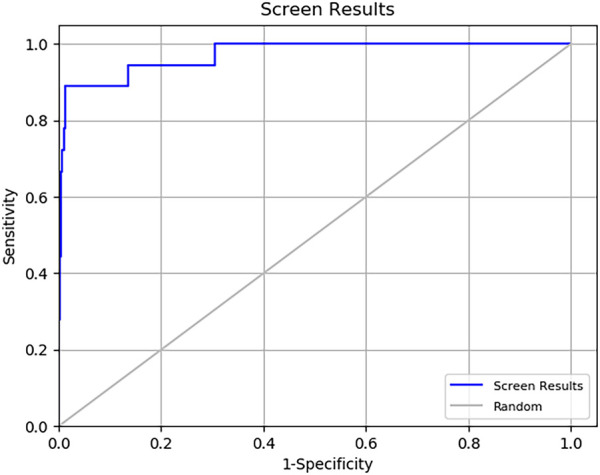
ROC Curve for the virtual screening of GyrB inhibitors showing a strong ability to distinguish actives from decoys (ROC = 0.97).

**FIGURE 6:**
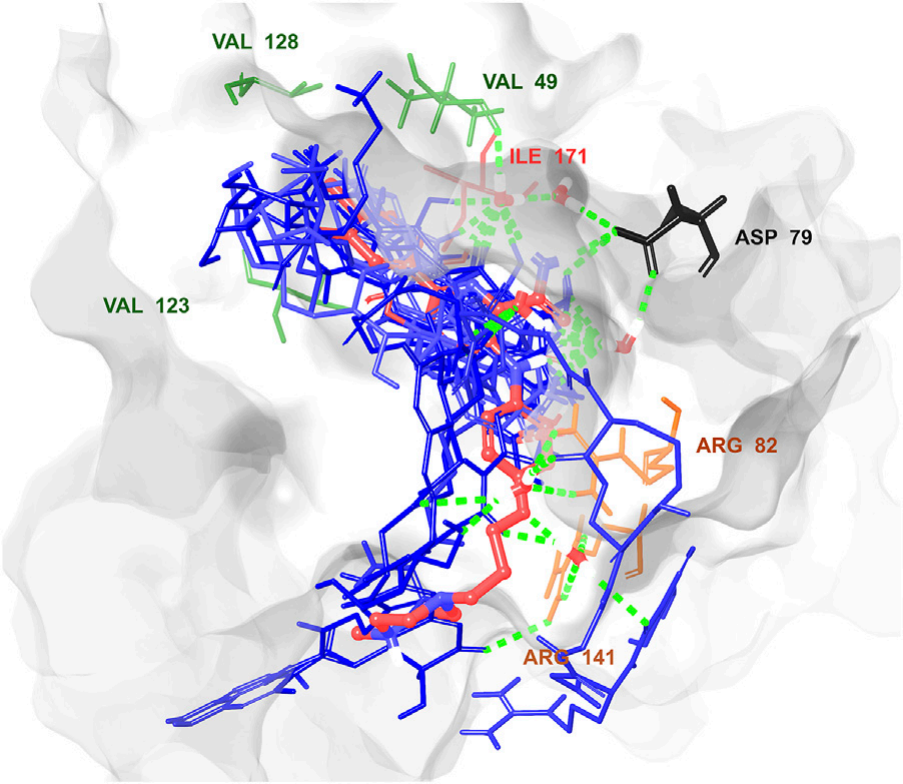
Superposition of the best poses of the top 12 hits relative to the original pose of the co-crystal ligand in the GyrB catalytic site showing the key interacting residues. The co-crystal ligand shown in red color, and the 12 hits in blue color. Created using Maestro interface of Schrödinger suite version 2023-1.

Multistep molecular docking study was performed using the Glide program as filtering protocol to investigate the binding modes of the tested library with the target protein. The crystal structure of the target protein, DNA gyrase B of MBT (PDB code: 4B6C) was downloaded from protein data bank (PDB) (https://www.rcsb.org). A microbial-based library of approximately 33000 compounds was docked into the ATP binding site of DNA gyrase B of MBT. Such compounds are expected to competitively inhibit ATP from binding to the enzyme’s catalytic region, thereby preventing its hydrolysis. Re-docking of the bound co-crystal ligand in the same binding site was performed in triplicates to validate the docking protocol (Figure 3). Root mean square deviation (RMSD) was calculated, and the obtained value of 1.757 ± 0.05 Å between the docked conformation and the original conformation validated the accuracy of the docking protocol. Initially, High Throughput Virtual Screening (HTVS) was adopted, and the top 10% hits were subsequently docked with Glide standard precision (SP). Finally, the top 10% from the previous step were subjected to Glide extra precision (XP) mode docking, which is associated with higher accuracy than the other docking modes. This filtering protocol ultimately afforded 12 hits, as shown in Table 1; Figure 4, with Glide XP scores ranging from −9.491 to −10.77 kcal/mol. Moreover, a step of enrichment calculations was undertaken to validate the docking results and assess the ability of the virtual screening process to effectively distinguish between active compounds and decoys for the potential inhibition of GyrB in MBT (Pandey et al., 2017). The virtual screening results demonstrated promising performance in identifying actives. A total of 18 active compounds were identified out of 1,466 ligands. The Boltzmann-Enhanced Discrimination of Relevance of Compounds (BEDROC values), with the highest at 0.890 (alpha = 8.0), indicated strong early enrichment of actives in the ranked list. The Receiver Operating Characteristic Curve (ROC) score of 0.97 further supported the efficacy of the screening in correctly discriminating actives from decoys, as shown in Figure 5. With Rank-Order Enrichment (RIE) of 14.92, the screening successfully outranked a significant number of decoys. The Area Under Accumulation Curve (AUAC) of 0.97 highlighted the ability to prioritize relevant compounds throughout the screening process. On average, 40 decoys were outranked by each active compound, suggesting good compound ranking. These metrics confirmed the validity of the docking procedure and its effectiveness in identifying GyrB inhibitors. The multistep docking protocol commenced with the screening of 33,000 compounds using High-Throughput Virtual Screening (HTVS), narrowing down the selection to 2,434 compounds. These were then subjected to Standard Precision (SP) docking, resulting in 440 compounds. Finally, Extra Precision (XP) docking was performed, identifying 50 top-ranking compounds. It is worth mentioning that, constraints were applied during the docking protocol based on interactions with key residue ASP79, through H-bonds (Shirude et al., 2013; Amorim et al., 2022). These interactions were critical in refining the selection, ensuring that the compounds aligned properly with the enzyme’s active site to maximize binding affinity and potential for inhibition. The identified compounds exhibited docking scores ranging from −9.27 to −12.61 kcal/mole, with 18 showed scores better than that of the co-crystal ligand (−10.523 kcal/mole). Following XP docking, MM-GBSA calculations were performed, and 12 compounds (Table 1) were selected based on their high binding affinity as well as their interactions with key residues in the enzyme binding pocket. These compounds demonstrated strong potential for further investigation due to their favorable binding free energies and critical residue interactions. The docking analysis of the selected 12 compounds (Table 1) revealed that Dipleosporalone B (−10.77 kcal/mol) and Glysperin B (−10.690 kcal/mol) had the best score, exceeding that of the co-crystal ligand (−10.523 kcal/mol). Dihydrospumigin N (−10.497 kcal/mol), and Fuscachelin A (−10.476 kcal/mol) closely approached the co-crystal ligand’s binding affinity. The remaining compounds exhibited relatively lower scores, ranging from −9.553 to −9.491 kcal/mol. The comparative analysis of binding free energies (Table 1) indicated that the co-crystal ligand had the strongest binding affinity (−81.05 kcal/mol), followed by Fuscachelin A (−73.21 kcal/mol). Other notable hits include Closthioamide (−66.26 kcal/mol), Erythrin (−65.68 kcal/mol), Dipleosporalone B (−64.93) and Glysperin B (−64.80 kcal/mol). The top-ranked hit Fuscachelin A is a cyclic peptide iron-sequestering siderophore synthesized by filamentous fungus (Dimise et al., 2008; Miethke and Marahiel, 2007). Siderophores have been described as having the potential for treatment of microbial infection. The second-ranked hit, Closthioamide is a unique polythioamide, isolated from the bacterium *Ruminiclostridium cellulolyticum*. Significant antibacterial activity has been reported for this hit specifically against multi-drug resistant strains such as MRSA (Methicillin-resistant *Staphylococcus aureus*) and VRE (Vancomycin-resistant enterococci) (Barth et al., 2024). This potent activity is believed to be mediated via the inhibition of ATPase function of DNA gyrase. Studies revealed its potential to allosterically modulate the ATPase activity of bacterial DNA gyrase, however, in our study it showed high affinity towards mycobacterial DNA gyrase B, suggesting potential direct inhibition mechanism (Chiriac et al., 2015). Such finding suggests Closthioamide’s potential as a promising new agent for both bacterial and mycobacterial infections. Further, the phenolic metabolite Erythrin derived from different plants belong to the genus Erythrina, displayed high affinity towards the enzyme binding pocket. Studies have reported the potent antimycobacterial activity of compounds isolated from these plants with structural similarity to Erythrin (Rukachaisirikul et al., 2007). This combination of evidence points to GyrB inhibition as a potential mode of action for compounds like Erythrin. Dipleosporalone B is a dimeric azaphilone compound marine-derived *Pleosporales* sp. fungus. Dimeric natural products are known for their potent biodynamic activities (Cao et al., 2020; Wang et al., 2023). Glysperin B which exhibited the highest docking score among the investigated compounds is a glycosylated benzamide derivative, part of a class of molecules recognized for their potent antibacterial activity (Kawaguchi et al., 1977). Furthermore, the virtual screening process identified two anthracycline derivatives, namely 1-Hydroxy-D-788-7 and Wexrubicin, which displayed similar binding affinity towards the enzyme. Interestingly, those hits are structurally related to the previously reported mycobacterial Gyr B inhibitor Epirubicin (Pakamwong et al., 2022). Further, several anthracyclines have demonstrated potent antibacterial activity against both Gram-positive and Gram-negative organisms, as well as significant antimycobacterial activity (Trenado-Uribe et al., 2018; Qun et al., 2023). Pyridindolol K2 is a β-carboline alkaloid, isolated from the culture broth of *Streptomyces* sp (Kim et al., 1997). Literature revealed several β-carboline derivatives as antimicrobial drug candidates (Szabo et al., 2021). Further, carbazole derivatives have been reported as potent inhibitors of MBT gyrase B, offering significant promise for the development of novel anti-TB agents (Pakamwong et al., 2024). Pyrronamycin B is an antibiotic that comprises a pyrrole-amide repeating unit in its structure, contributing to its potent antibacterial activity against both Gram-positive and Gram-negative bacteria. Research has shown that its antimicrobial properties are primarily mediated through the inhibition of DNA gyrase (Asai et al., 2000). Lampteroflavin is a flavin derivative, structurally related to Riboflavin, commonly known as vitamin B2. It is isolated from the luminous mushroom, *Lampteromyces japomcus* (Takahashi et al., 1991). Research reveals that bacteria are likely to develop resistance to antimicrobial flavins at a significantly lower rate (Pedrolli et al., 2014). Dihydrospumigin N is a polycyclic natural product isolated from Cyanobacteria. It is a member of the spumigin family of secondary metabolites, known for their bioactive properties (Sanz et al., 2017). Overall, most of the hits we identified have been previously reported to exhibit antimicrobial properties, with some also demonstrating gyrase inhibitory activity against both Gram-positive and Gram-negative bacteria. Nevertheless, this study is the first to identify these compounds as potential candidates targeting the ATPase activity of MBT DNA gyrase. Furthermore, the retrieved hits in the present study demonstrate gyrase inhibitory properties, further validating our docking protocol. Moreover, to the best of our knowledge, this study is the first to utilize the high-resolution crystal structure of mycobacterial GyrB to screen natural products library, further strengthening the reliability of the findings (Shirude et al., 2013). Additionally, as explained in the following paragraph, the identified hits demonstrated superior binding affinity compared to most previously reported natural products or repurposed drugs, exhibiting enhanced interaction with the enzyme. As a result, they are expected to represent novel candidates with higher efficacy and lower resistance potential. Notably, The DNA gyrase enzyme pocket, particularly the GyrB subunit, is highly conserved among *Mycobacterium* species. This conservation implies that the identified hits could potentially inhibit GyrB across different *Mycobacterium* species, including MBT making them valuable candidates for developing broad-spectrum anti-mycobacterial agents (Fu et al., 2009). All 12 hits were properly docked into the ATP-binding site of the GyrB subunit of MBT DNA gyrase and exhibited binding interactions similar to those of the co-crystal ligand (Table 1; Supplementary Figure S3). A variety of molecular interactions were identified as characterizing the binding of the hits to the enzyme’s active site. These interactions included ionic bonds, direct and water-mediated hydrogen bonding, Pi-cation interactions, and Van der Waals (VdW) forces. As reported by several studies (Shirude et al., 2013; Hameed et al., 2014; Tomasic et al., 2021), two key sites in the enzyme pocket are essential for high-potency inhibitors, complemented by hydrophobic interactions within the hydrophobic loop. Site 1 involves interactions with ASP79, which may occur via direct hydrogen bonds or water-mediated hydrogen bonds. Site 2 includes interactions with GLY83 alongside ARG141 (via direct or water-mediated hydrogen bonds) and ARG82 (via pi-cation interactions). Additionally, hydrophobic interactions with residues in the hydrophobic pocket—VAL149, VAL123, VAL125, VAL128, and ILE171 are critical for enhancing inhibitor potency. These interactions collectively contribute to binding affinity and specificity. Importantly, the identified hits, similar to the co-crystal ligand (Figure 6; Supplementary Figure S3), engaged in multiple interactions with residues located in the two key sites (ASP79, ARG82, and ARG141). Additionally, they form interactions with nearby residues such as GLU48, GLU56, ASN52, and HIS89, which considered essential for enhanced binding, as reported by Tambe et al. (2020). These supplementary interactions further enhanced their binding stability and strengthened the overall interaction network within the enzyme pocket. Overall, the nature of the interactions consistently contributed to ligand stability across the identified hits, emphasizing their critical role in achieving high binding affinity. Taken together, these interactions provided a robust foundation for ligand binding and represent crucial targets for the future optimization of the hits identified in this study. Considering the higher docking scores and binding affinity of Fuscachelin A, it was selected as representative example for further analysis to gain more insight into its binding characteristics. The best docking pose of Fuscachelin A obtained at Glide XP-docking level was displayed in Supplementary Figure S3. Fuscachelin A formed crucial interactions within the enzyme pocket. It engaged with ASP79 and ARG141 through water-mediated H-bonds and formed an H-bond with ARG82 instead of a pi-cation interaction observed for the co-crystal ligand. Additionally, it established a salt bridge with ASP142 in site 1 and formed four hydrogen bonds, significantly stabilizing the complex. The decacyclic ring protruded to the solvent exposed area. The top 12 ranked hits positioned perfectly and similarly to the co-crystal ligand (Figure 6), with larger compounds showing their polar parts exposed to the solvent. This orientation suggests that the polar regions of the ligands interact with the surrounding aqueous environment, potentially enhancing solubility and binding efficiency.

**TABLE 1:** Docking scores and the ligand-target interactions forces of the top 12 hits.

No	NPAtlas ID	Name	Binding free energy (kcal/mol)	Docking score (kcal/mol)	Key interactions
Co-crystal ligand			**‒81.05**	**‒10.523**	1. Pi-cation with ARG82 (3.46 Å) 2. Water mediated H-bonds with the following residues: i. VAL49 (2.09 Å and 1.90 Å) ii. ASP79 (1.83 Å and 1.92 Å) iii. GLY83 (1.83 Å and 1.98 Å) iv. ARG141 (1.6 Å and 2.33 Å) 3. Direct H-bonds i. ASP79 (2.33 Å)
1	NPA029673	Dipleosporalone B	‒64.93	‒10.77	1. Water mediated H-bonds with the following residues: i. ASP79 (2.05 Å and 1.92 Å) ii. ARG82 (1.94 Å and 2.2 Å) iii. GLY83 (2.05 Å and 1.98 Å) 2. Direct H-bonds i. ARG141 (2.5 Å) ii. ASP79 (2.69 Å)
2	NPA021035	Glysperin B	‒64.80	‒10.690	1. Pi-cation with ARG82 (3.67 Å) 2. Water mediated H-bonds with the following residues: i. ASP79 (2.05 Å and 1.92 Å) ii. GLY83 (2.05 Å and 1.98 Å) iii. ARG141 (2.15 Å and 2.33 Å)
3	NPA028815	Dihydrospumigin N	‒62.10	‒10.497	1. Water mediated H-bonds with the following residues: i. ASP79 (2.12 Å and 1.99 Å) ii. GLY83 (2.12 Å and 1.98 Å) 2. Direct H-bonds GLY83 (1.89 Å)
4	NPA020353	Fuscachelin A	‒73.21	‒10.476	1. Water mediated H-bonds with the following residues: i. ASP79 (1.92 Å and 1.99 Å) ii. ARG82 (1.98 Å and 2.20 Å) iii. ARG141 (2.69 Å and 2.33 Å) iv. GLY83 (1.99 Å and 1.98 Å)
5	NPA019546	1-Hydroxy-D-788-7	‒60.37	‒10.253	1. Water mediated H-bonds with the following residues: i. VAL49 (1.8 Å and 1.90 Å) ii. ASP79 (2.27 Å and 1.92 Å) iii. GLY83 (2.27 Å and 1.98 Å)
6	NPA002954	Pyridindolol K2	‒64.51	‒9.590	1. Water mediated H-bonds with the following residues: i. VAL49 (1.8 Å, 1.86 Å and 1.85 Å) ii. ASP79 (1.77 Å and 1.99 Å) iii. GLY83 (1.77 Å and 1.98 Å)
7	NPA032921	Wexrubicin	‒60.41	‒9.553	1. Water mediated H-bonds with the following residues: i. VAL49 (1.91 Å and 1.90 Å)
8	NPA005438	Lampteroflavin	‒63.44	‒9.544	1. Water mediated H-bonds with the following residues: i. ASP79 (1.63 Å and 1.99 Å) ii. GLY83 (1.63 Å and 1.98 Å)
9	NPA007770	Not named	‒62.67	‒9.544	1. Water mediated H-bonds with the following residues: iii. ASP79 (1.70 Å and 1.99 Å) iv. GLY83 (1.70 Å and 1.98 Å) 2. Direct H-bonds i. GLY83 (1.89 Å)
10	NPA007224	Pyrronamycin B	‒60.54	‒9.525	1. Pi-cation with ARG82 (3.64 Å) 2. Water mediated H-bonds with the following residues: i. VAL49 (1.72 Å and 1.90 Å) ii. ASP79 (2.31 Å and 1.92 Å) iii. GLY83 (2.18 Å and 1.98 Å) iv. ARG141 (1.96 Å and 2.33 Å)
11	NPA000223	Erythrin	‒65.68	‒9.492	1. Water mediated H-bonds with the following residues: i. VAL49 (1.64 Å and 1.90 Å) ii. ASP79 (1.70 Å and 1.99 Å) iii. GLY83 (1.70 Å and 1.98 Å) 2. Direct H-bonds i. GLY83 (1.89 Å)
12	NPA016415	Closthioamide	‒66.26	‒9.491	1. Water mediated H-bonds with the following residues: i. ASP79 (1.85 Å and 1.99 Å) ii. ARG82 (2.79 Å and 2.33 Å) iii. GLY83 (1.85 Å and 1.98 Å)

Bold values represent the binding free energy and docking score of the co-crystal ligand, serving as a reference for comparison with the test compounds.

**FIGURE 7:**
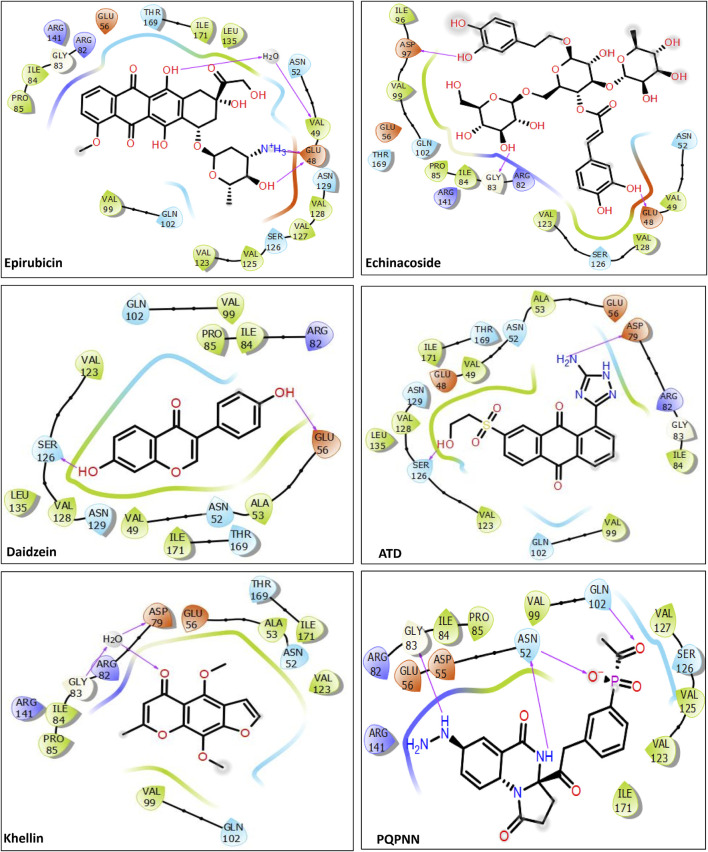
2D interactions of the previously reported inhibitors with MBT Gyr B (PDB ID: 4B6C).

To contextualize our findings, a comparative assessment was performed focusing on the identified microbial natural products in relation to previously reported natural product inhibitors and repurposed drugs (Balasubramani et al., 2020; Amorim et al., 2022; Arevalo and Amorim, 2022; Jagatap et al., 2023). As depicted in Table 2, all reported inhibitors (except Epirubicin) had higher docking scores compared to our identified hits **1–12**. Moreover, except for Epirubicin (binding free energy −71.97 kcal/mol), the binding affinities of our identified hits (−60.37 to −73.21 kcal/mol) were superior to those of the reported inhibitors (−20.74 to −46.43 kcal/mol). In addition, hits **3**, Fuscachelin A, the top hit in our study, displayed binding affinity exceeded that of Epirubicin. Epirubicin, is a known intercalating agent, demonstrated a high binding affinity (−71.97 kcal/mol) and docking score (−10.5 kcal/mol) with mycobacterial Gyr B. Epirubicin features planar aromatic structures allowing it to establish hydrophobic as well as Pi-Pi stacking interactions with amino acid residues residing in the ATP binding site such as VAL123, VAL125, VAL128, and ILE171 of Gyr B. Moreover, other interactions including water-mediated H-bond with VAL49 and two direct H-bonds and one ionic bond with GLU48 further strengthening its binding affinity. However, Epirubicin is not suitable for DNA gyrase inhibition or tuberculosis treatment due to its potential for immunosuppression and cardiotoxic effects (Banke et al., 2018). Additionally, unlike our identified hits, most reported natural product inhibitors failed to establish significant interaction forces with key residues in the enzyme binding pocket (VAL49, ASP79, ARG82, GLY83, and ARG141) (Figure 7), suggesting a potentially lower inhibitory effect compared to our identified compounds (Shirude et al., 2013). Furthermore, virtual screening conducted by Amorim et al. (2022) identified N-(2-hydroxyethyl)-9,10-dioxoanthracene-2-sulfonamide as the best-scored hit. However, this compound failed to establish meaningful interactions with crucial residues in the binding pocket, such as ASP79, VAL49, and ARG82, which are essential for effective enzyme inhibition. To overcome this limitation, they designed the Aminotriazole Anthraquinone Derivative (ATD) (Figure 7), incorporating structural modifications to improve interactions within the binding site. As a result, ATD successfully formed an interaction with ASP79, but the overall binding affinity and binding free energy were suboptimal compared to our identified compounds. Similarly, Arevalo and Amorim (2022) identified a pyrrolo [1,2-a]quinazoline derivative (PQN) via virtual screening of a natural product database. However, PQN also lacked interactions with key residues, such as ASP79 and ARG141. To enhance its binding properties, they synthesized the PQPNN derivative (Figure 7), specifically designed to optimize interactions with the critical residues. Although PQPNN established interaction with ASP79, its binding free energy and affinity were inferior, remaining two-fold less effective than the hits identified in our study (binding free energy >‒35 kcal/mol). In contrast, our study identified naturally occurring compounds that inherently interact with multiple key residues in the enzyme’s binding pocket, including ASP79, VAL49, ARG82, GLY83, and ARG141. These strong and meaningful interactions suggest a higher inhibitory potential, surpassing the modified derivatives reported by Amorim et al. (2022), Arevalo and Amorim (2022). Notably, our compounds did not require any structural modifications to achieve these superior binding affinities, making them more viable as lead compounds for further development. Additionally, the computational expense and time required for designing and testing derivatives, as seen in ATD and PQPNN, highlight another advantage of our hits. The natural origin of our compounds also aligns with the broader trend of utilizing microbial natural products for their inherent diversity, potency, and lower resistance potential. This further substantiate the significance of our findings and their potential impact on developing novel inhibitors targeting *M. tuberculosis* DNA gyrase B.

**TABLE 2:** Docking scores and binding free energies in kcal/mol of the reported mycobacterial DNA gyrase B inhibitors.

No.	Name of the reported inhibitor	Docking score (kcal/mol)	Binding energy (kcal/mol)	References
1	Epirubicin	‒10.500	‒71.97	Balasubramani et al. (2020)
2	Echinacoside	‒7.849	‒46.43	Balasubramani et al. (2020)
3	Daidzein	‒5.571	‒32.49	Jagatap et al. (2023)
4	N-(2-hydroxyethyl)-9,10 dioxoanthracene-2-sulfonamide	‒4.782	‒47.91	Amorim et al. (2022)
5	Aminotriazole Anthraquinone Derivative (ATD)	‒5.123	‒35.19	Amorim et al. (2022)
6	Khelline	‒4.770	‒37.57	Jagatap et al. (2023)
7	PQPNN	‒3.891	‒20.74	Arevalo and Amorim (2022)

### 3.3 MM-GBSA calculations

**FIGURE 8:**
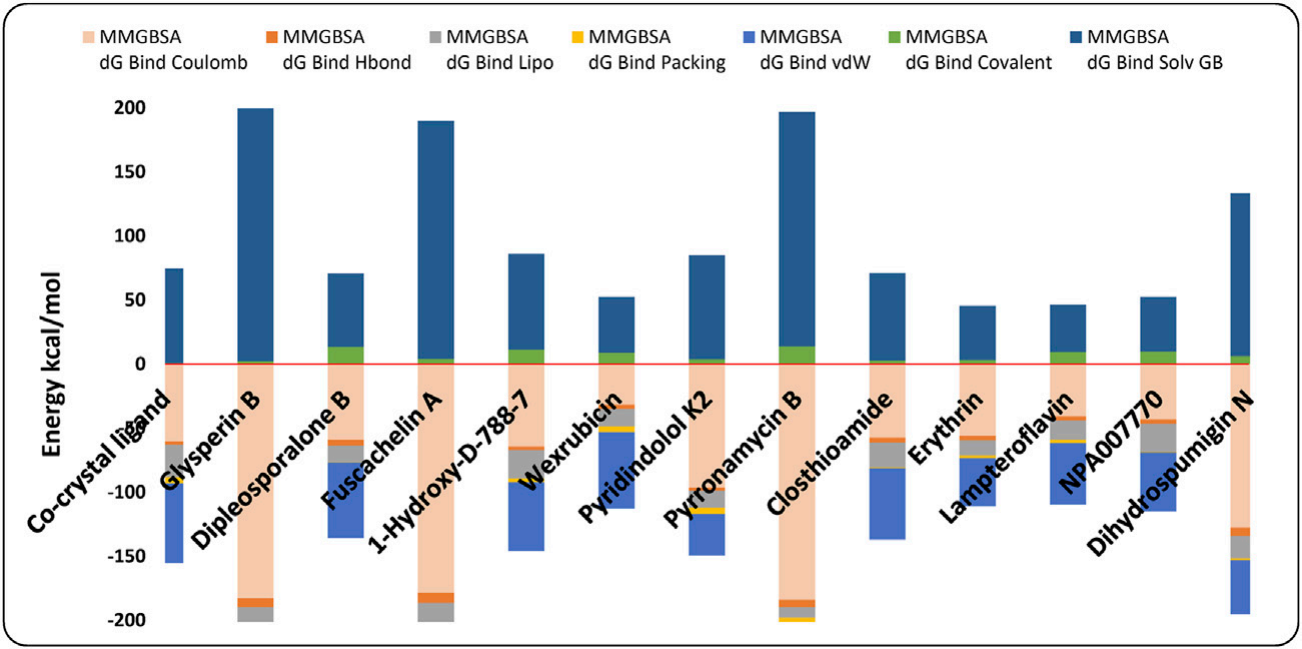
Contribution of various energy terms to the binding free energy of the co-crystal ligand and the top 12 hits. The analysis highlights how different energy components, such as van der Waals, electrostatic, and solvation energies, influence the overall binding affinity.

Following the multistage docking simulations, the docked poses of the top 12 hits and the co-crystal ligand were rescored by MM-GBSA method. In Prime to support the docking study and obtain more accurate predictive binding free energy (ΔG_bind)_. The energy was calculated and the more negative value indicates a stronger binding affinity. It has been observed that the identified hits demonstrated binding free energies ranging from −60.37 to −73.21 kcal/mole (Table 1; Figure 8) signifying their high affinities towards the GyrB active site. Fuscachelin A displayed the highest affinities among the top 12 hits with binding free energies of −73.21 kcal/mole. An analysis of the energy terms contributing to the overall binding free energy of the top 12 hits is provided. As shown in Figure 8, the energy breakdown highlights the interaction profile of each compound compared to the co-crystal ligand, emphasizing specific binding contributions. The co-crystal ligand showed moderate MMGBSA dG Bind Coulomb (−60.35) and dG Bind Lipo (−25.19), indicating balanced electrostatic and hydrophobic interactions. As illustrated in Figure 8, hit compounds such as Pyrromycin B and Glyperin B outperformed the co-crystal ligand in Coulombic (−184.2 and −182.86, respectively), suggesting stronger electrostatic contributions. However, these hits had weaker dG Bind Lipo values, hinting at a potential gap in hydrophobic interactions. Comparatively, Fusachelin A had favorable Coulomb (−178.64) and dG Bind Covalent (−56.84) terms, indicating strong polar and covalent contributions. In contrast, weaker hits like Lampteroflavin had lower energy values across all categories, indicating overall reduced binding affinity. The van der Waals (vdW) and covalent terms vary significantly. Hits such as Glyperin B and Pyrromycin B excel in vdW (−59.37 and −55.63, respectively) (Figure 8) but showed limitations in dG Bind Solv GB, suggesting room for optimization of solvation effects. To improve binding affinities, enhancing hydrophobic interactions (dG Bind Lipo) and fine-tuning solvation energies (dG Solv GB) could provide more balanced binding profiles. For hits with strong electrostatics, such as Pyrromycin B, improving lipophilic interactions may further strengthen the binding. Optimization of hydrogen bonding networks could also enhance specific contributions seen in weaker terms like dG Bind Hbond (Figure 8). In addition, detailed analysis was performed to evaluate the energy terms of each compound based on its structure, with the goal of guiding potential optimization strategies. The energy breakdowns of the compounds analyzed (Figure 8) showed strong Coulombic interactions driven by hydroxyl, amide, and amino groups, which supported extensive electrostatic and hydrogen-bonding interactions. These interactions were significant in Glyperin B, Dipleosporalone B, Fuscachelin A, and several others, with large sugar frameworks or polar groups contributing to van der Waals interactions. However, the high polarity in many of these compounds, such as in Glyperin B and Wexrubicin, led to considerable solvation penalties. Lipophilic contributions were moderate, with hydrophobic regions like the aromatic cores or methyl groups balancing polarity. The co-crystal ligand exhibited similar Coulombic interactions but also demonstrated notable hydrophobic and basicity contributions from the 4-methylpiperazine group, enhancing binding stability. Further, Figure 8 indicated that, compared to the other compounds, the co-crystal ligand exhibited a better balance between hydrophobicity and polarity, which could potentially result in improved binding efficiency if optimized. Some compounds, like NPA007770 and Closthioamide, exhibited higher polarity and solvation penalties, suggesting the need for further structural refinement to optimize their binding properties. Dihydrospumigin N exhibited strong Coulombic (−127.88 kcal/mol) and van der Waals (−42.24 kcal/mol) contributions (Figure 8), driven by polar groups and hydrophobic regions, but faces high solvation penalties (127.42 kcal/mol). Its moderate hydrophobicity and weak packing suggest room for optimization. Enhancing hydrophobic interactions and reducing desolvation costs could improve binding affinity. Overall, optimizing both polar and nonpolar interactions while mitigating solvation penalties is crucial for enhancing hit potential in the development of antimycobacterial drugs targeting the ATPase activity of Gyr B. By refining structural features to balance electrostatic, van der Waals, and lipophilic contributions, we can improve binding efficiency. These efforts are essential for advancing the design of effective anti-TB hits targeting MBT.

### 3.4 *In silico* ADMET profiling

In the past five decades, the evaluation of the physicochemical and pharmacokinetics profiles of drug candidates in the early phases has become an integral component of the drug design and development processes (Caldwell et al., 2003). It involves initial assessments of Absorption, Distribution, Metabolism, Excretion, and Toxicity (ADMET) parameters in order to exclude hits associated with suboptimal or inadequate ADMET profiles from further consideration as potential drug candidates and are thus represent an ideal filtration approach. In the present work, computer-based methods were utilized to predict the ADMET and physicochemical profiles of the top 12 hits identified for their potential to inhibit MBT DNA Gyrase B. The QikProp analysis revealed the physicochemical and pharmacokinetic properties of the compounds (Supplementary Table S1), offering insights into their drug-likeness and ADME profiles. In this context, an overall ADME-compliance score (drug likeness parameter #stars) was used to calculate the number of descriptors for the top 12 hits that fall outside the permissible range of values for 95% of known drugs (Mohamed et al., 2024). Glysperin B and Fuscachelin A had the highest #stars values (18 each), suggesting significant deviations from drug-likeness criteria. In contrast, compounds like Wexrubicin (3), Erythrin (1), and Lampteroflavin (6) showed values within or near the acceptable range, suggesting fewer property violations. Molecular weight (mol_MW), an important factor influencing bioavailability, was recommended to be within 130–725. Several compounds, such as Glysperin B (919) and Fuscachelin A (1,030), exceeded this range, indicating potential absorption challenges due to their large size. Similarly, the solvent-accessible surface area (SASA), ideally between 300 and 1,000, was significantly higher for Fuscachelin A (1,547), suggesting potential issues with solubility and permeability. The SASA components—FOSA (hydrophobic surface area), FISA (polar surface area), and PISA (pi surface area)—varied widely across the compounds. Fuscachelin A, in particular, exhibited an exceptionally high FISA value (799.9), reflecting excessive polarity, which likely hindered its membrane permeability. Hydrogen bond donor (DonorHB) and acceptor (AccptHB) values, critical for solubility and target binding, revealed that Glysperin B had excessively high values (DonorHB: 17, AccptHB: 34), which may have increased its hydrophilicity and reduced its permeability. LogP (QPlogPo/w) and solubility (CIQPlogS) values, crucial for absorption and distribution, indicated limitations for several compounds. For example, Glysperin B displayed excessive hydrophilicity with a QPlogPo/w of −7.01, while Dipleosporalone B exhibited very poor solubility with a CIQPlogS of −10.0. Permeability metrics such as QPPCaco and QPPMDCK, which assessed intestinal permeability and drug transport potential, showed poor results for most compounds. However, Pyridindolol K2 and Closthioamide demonstrated relatively better permeability profiles. Evaluating drug bioavailability is critical for analyzing how drugs are absorbed, distributed, metabolized, and excreted, as well as for establishing suitable therapeutic dosages. This assessment is also fundamental in drug development processes and in comparing the effectiveness of different delivery methods (Stielow et al., 2023). The human oral absorption metrics (%Oral Absorption and Human Oral Absorption categories) highlighted additional challenges. Only Pyridindolol K2 achieved high oral absorption (81%), while the other hits were classified as having low or poor absorption potential. The therapeutic efficacy of any drug is greatly affected by its binding to the plasma proteins, as this determines the amount of the free drug that can traverse the cellular membranes (Ntie-Kang, 2013). In this regard, the logKHSA parameter was utilized to assess the potential of the screened hits to bind to human albumin. The logKHSA parameter assesses plasma protein binding, which influences drug distribution and pharmacokinetics. Compounds like Dipleosporalone B, Wexrubicin, Pyridindolol K2, and Closthioamide demonstrated optimal binding (logKHSA within −1.5 to 1.5), ensuring balanced bioavailability and retention. In contrast, Glysperin B (−3.265) and Fuscachelin A (−2.8) exhibited very weak binding, potentially leading to rapid clearance or toxicity due to high free drug concentrations. While most top 12 hits fell within the permissible range, weak-binding ones may require structural optimization. In drug development, blocking the human ether-a-go-go-related gene (hERG) channel by small molecules is a significant concern. Inhibition or disruption of hERG channel activity by drug compounds can prolong the QT interval, potentially leading to severe cardiotoxicity (Lamothe et al., 2016). Toxicity concerns were apparent in QPlogHERG values, where compounds such as Glysperin B and Closthioamide (both −8.2) raised significant cardiotoxicity risks by exceeding the threshold of −5. Assessment of on drug metabolism at early stages of drug discovery is a crucial step in optimizing lead compounds to achieve ideal pharmacokinetic and pharmacodynamic profiles (Zhang and Tang, 2018). The #metab parameter, which predicts the number of metabolic reactions a compound is likely to undergo, was another critical factor. The acceptable range is 1–8, and most compounds exceeded this limit, with Fuscachelin A (16) and Glysperin B (15) showing particularly high values. These results indicated that these compounds were highly prone to metabolism, potentially leading to rapid clearance or the production of undesirable metabolites. Conversely, compounds such as Pyridindolol K2 (3) and Closthioamide (6) had values closer to the acceptable range, indicating better metabolic stability. Most compounds adhered to the Rule of Five (maximum four violations) and the Rule of Three (maximum three violations), demonstrating general drug-likeness. Among the tested compounds, Pyridindolol K2 stood out with favorable absorption, permeability, and metabolic stability, making it a promising hit. However, most compounds, particularly Glysperin B and Fuscachelin A, required structural modifications to address solubility, permeability, metabolism, and toxicity issues identified in the analysis. This assessment emphasized the importance of optimizing these parameters to improve the pharmacokinetic profiles of the identified hits. Further, parameters verified by ADMETlab webserver were evaluated for the top 12 hits, and the results are tabulated on Supplementary Table S2. These parameters comprise, human hepatotoxicity, drug-induced liver injury, AMES toxicity, rat oral acute toxicity, FDA maximum daily dose, carcinogenicity, mutagenicity, skin sensitization, eye corrosion, eye irritation, and respiratory toxicity. The toxicity profiles of the compounds revealed significant variations in their safety and potential risks. Glysperin B and Fuscachelin A consistently showed high toxicity across multiple parameters, including hepatotoxicity (0.97 and 0.95), mutagenicity (0.99), and skin sensitization (1), indicating a high risk for both human liver damage and genetic mutations. In contrast, NPA007770 and Dihydrospumigin N exhibited lower toxicity values, particularly for drug-induced liver injury (0.01) and carcinogenicity (0.02), making them relatively safer. Wexrubicin and Pyridindolol K2 had moderate toxicity risks in liver injury (0.99 and 0.53) and mutagenicity (0.99 and 0.92), but they also showed higher carcinogenic potential. Closthioamide and Pyrronamycin B showed a mixed toxicity profile with moderate skin sensitization and irritation risks, with Closthioamide also having a notable potential for eye irritation (0.65). Overall, NPA007770 and Dihydrospumigin N appeared to be safer, while Glysperin B and Fuscachelin A presented significant toxicity challenges that required careful consideration in drug development. Pyridindolol K2 exhibited moderate toxicity risks, particularly in liver injury (0.53) and mutagenicity (0.92), with a higher potential for carcinogenicity (0.92). It also showed moderate skin sensitization (0.67) and eye irritation (0.17). While not as toxic as Glysperin B or Fuscachelin A, it still posed notable concerns, particularly in terms of carcinogenicity and mutagenicity, which would require careful assessment during further development. In conclusion, while NPA007770 and Dihydrospumigin N demonstrated relatively safer toxicity profiles, compounds like Glysperin B and Fuscachelin A posed significant risks, highlighting the need for further optimization and careful evaluation in the drug development process. Pyridindolol K2 on the other hand demonstrated a more balanced toxicity profile compared to the highest-risk compounds but still warranted attention in safety evaluations. In conclusion, among the 12 hits, Pyridindolol K2 demonstrated the best drug-likeness, exhibiting no ADME deviations, which indicated optimal pharmacokinetic properties. Erythrin and NPA007770 displayed minimal ADME issues, suggesting they also have strong potential, though some minor refinements were necessary. In contrast, Wexrubicin and 1-Hydroxy-D-788-7 showed moderate drug-likeness, with noticeable deviations in ADME properties that would require optimization to improve their pharmacokinetic profiles. Regarding toxicity, Pyridindolol K2 and Erythrin exhibited favorable toxicity profiles, with low risks for hepatotoxicity and mutagenicity, making them safer hit for development. NPA007770 also showed promising toxicity data, while Wexrubicin and 1-Hydroxy-D-788-7 exhibited moderate toxicity concerns, particularly regarding liver injury and mutagenicity, indicating a need for further optimization to reduce potential harmful effects. To obtain meaningful insights, we conducted a comprehensive comparison of the ADMET properties of our identified hits with those of SPR720 (Supplementary Table S1). This approach was necessitated by the fact that, to date, no approved drug existed for the treatment of tuberculosis targeting MBT gyrase B, with SPR720, a benzimidazole derivative, being the sole candidate under clinical development in this context (Talley et al., 2021). Pyridindolol K2 demonstrated the most favorable properties compared to SPR720 in QikProp analysis, emerging as the leading hit. It exhibited high human oral absorption (81%), excellent compliance with the Rule of Five (0 violations), and favorable solubility and lipophilicity (QPlogPo/w: 1.5). Its blood-brain barrier penetration (QPlogBB: −1.3) further supported its potential. SPR720 served as the reference with reasonable drug-likeness and low HERG inhibition risk, but its lower oral absorption (24.9%) and higher molecular weight limited its efficiency. In contrast, 1-Hydroxy-D-788-7 and Erythrin displayed significant limitations, such as poor absorption and excessive rotatable bonds, which impacted bioavailability. Erythrin also showed a high risk of HERG inhibition, raising safety concerns. Furthermore, we also made a comparative analysis of the ADMETLab results, which are presented in Supplementary Table S2, to evaluate the safety profiles of the hits alongside SPR720. Pyridindolol K2 demonstrated a balanced safety profile with moderate risks in hepatotoxicity (0.48) and mutagenicity (0.92), and concerns in carcinogenicity (0.92) and skin sensitization (0.67), making it a promising hit compared to SPR720. Erythrin showed low hepatotoxicity (0.09) and excellent respiratory safety (0.07), but posed risks in eye irritation (0.65) and moderate mutagenicity (0.22), making it safer than SPR720 in liver and respiratory safety but requiring refinement. In contrast, 1-Hydroxy-D-788-7 exhibited significant risks, with high hepatotoxicity (0.93), mutagenicity (0.98), and respiratory toxicity (0.93), making it less favorable than SPR720. Overall, Pyridindolol K2 emerged as the most promising hit, while the others demonstrated potential but required further refinement for both pharmacokinetic optimization and toxicity mitigation.

### 3.5 Pharmacophore modeling

**FIGURE 9:**
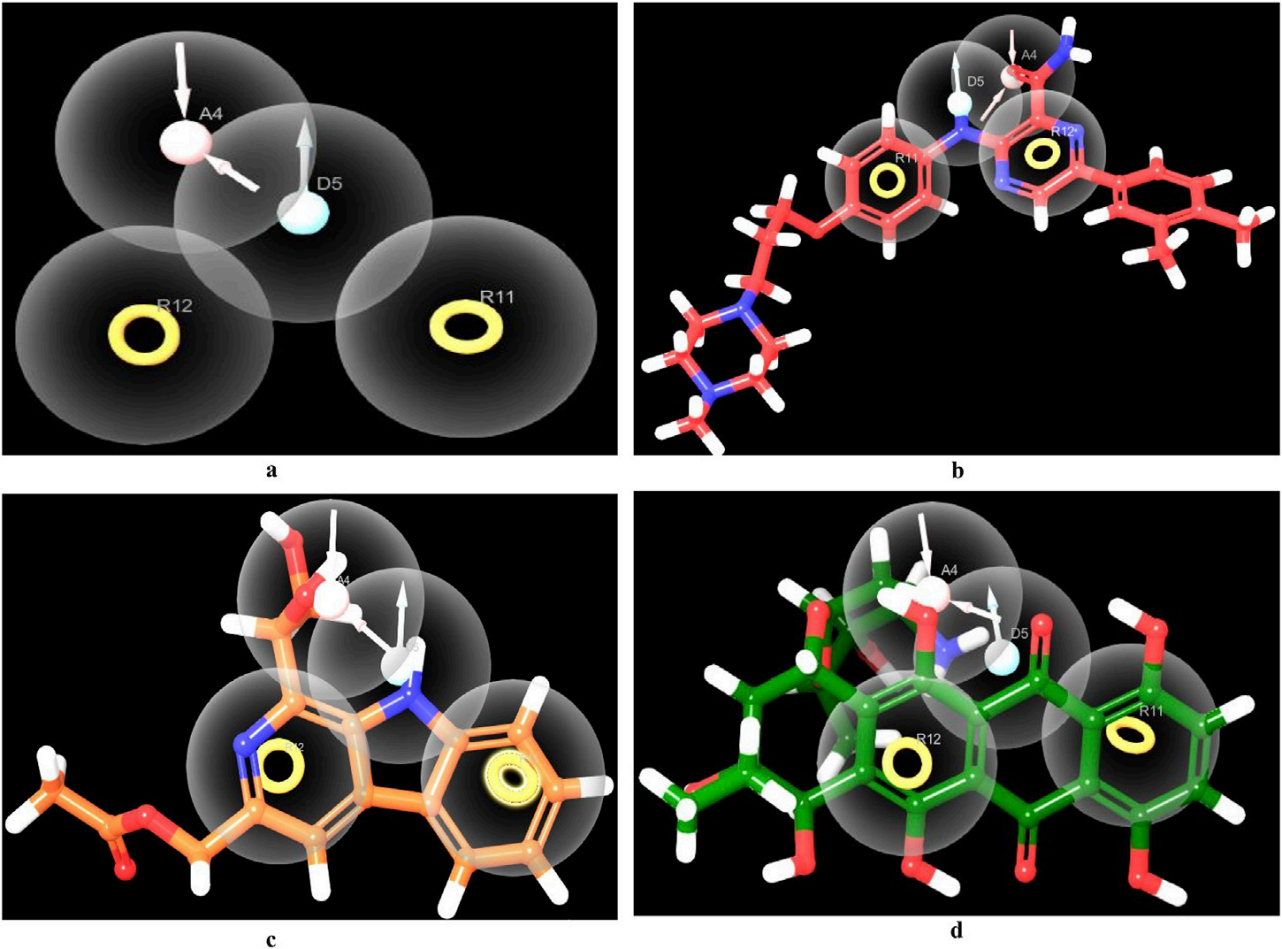
Pharmacophore screening. Created using the Maestro interface of Schrödinger Suite version 2023-1. **(A)** The best-fitted four-point pharmacophore model generated using known actives. The pharmacophoric features are represented as spheres, including two aromatic ring systems (R), one hydrogen bond donor (D), and two hydrogen bond acceptors (A). **(B)** 3D feature alignment of the co-crystal ligand (red), showing the alignment of the compound to the pharmacophore model. **(C)** 3D feature alignment of Pyridindolol K2 (Orange), showing the alignment of the compound to the pharmacophore model. **(D)** 3D feature alignment of 1-Hydroxy-D-788-7 (green), showing the alignment of the compound to the pharmacophore model.

Amongst the diverse computer-aided drug design approaches, pharmacophore-based drug design is considered an efficient approach for the rational design of novel bioactive molecules. Pharmacophore modeling is most commonly applied to virtually screen small molecule libraries for potential modulators of specific biological effects (Voet et al., 2014). Herein, we conducted pharmacophore modeling to identify hits that match the essential features required for potent MBT DNA Gyrase B inhibitors. The Phase software in the Schrödinger Drug Discovery Suite was used to generate the 3D pharmacophore model. For this purpose, a training set composed of 67 previously reported inhibitors of DNA gyrase B were selected based on their known inhibitory activity of MBT DNA Gyrase B (Jeankumar et al., 2013; Kale et al., 2013; Shirude et al., 2013; Jeankumar et al., 2014; Kale et al., 2014; Reddy et al., 2014; Renuka et al., 2014; Jeankumar et al., 2015; Locher et al., 2015; Medapi et al., 2015a; Medapi et al., 2015b; Saxena et al., 2015) (Supplementary Table S3). They were employed to generate several pharmacophore hypotheses (Table 3) with diverse combinations of chemical features. Then, Phase HypoScore was used to rank them using an internal validation method. As illustrated in Figure 9, the best-fitted four-point pharmacophore hypothesis (ADRR_1) was elected as the best from the generated hypotheses to perform virtual screening. It consists of two aromatic rings features (R), one hydrogen bond acceptor feature (A) and one hydrogen bond donor feature (D). Subsequently, the 12 hits were screened against the best-fitted hypothesis, and the results (Table 4; Figure 9) led to the identification of 6 hits as the best (PhaseScreenScores ranged from 0.94 to 2.03), matching all four chemical features of the pharmacophore model. The co-crystal ligand, as shown in Figure 9, aligned well with the pharmacophoric features, achieving a PhaseScreenScore of 2.3, reflecting strong compatibility with the binding site. Key interactions observed in the ligand binding included the donor feature at Site-1, which likely formed a water-mediated hydrogen bond with ASP79, and the aryl or heteroaryl groups at Site-2, which engaged in Pi-cation interactions with ARG82. Hydrophobic contacts with residues VAL123, VAL125, VAL128, and ILE171 further stabilized the ligand. Among the identified hits, Pyridindolol K2 emerged as the top candidate, with the highest PhaseScreenScore and Fitness Scores (2.03), although its moderate Align Score (0.562) suggested room for spatial optimization. Pyrronamycin B exhibited excellent spatial alignment (Align Score: 0.949) despite moderate PhaseScreenScore and Fitness Scores (1.32), suggesting its potential as a promising hit. 1-Hydroxy-D-788-7 achieved the highest Align Score (1.12) but recorded the lowest PhaseScreenScore and Fitness Scores (0.95), warranting further investigation for potential activity. Erythrin displayed balanced scores (PhaseScreen/Fitness: 1.42, Align: 0.783), indicating good feature matching and spatial alignment. Closthioamide demonstrated strong spatial alignment (Align Score: 0.915) but lower PhaseScreenScore and Fitness Scores (0.94), while Lampteroflavin showed moderate performance across all metrics, with a low Align Score (0.540), indicating weaker spatial compatibility. These results reinforce the importance of balancing spatial alignment and pharmacophoric feature matching for optimal ligand binding. The pharmacophoric features align closely with the binding pockets described by Shirude et al. (2013). The co-crystal ligand and identified hits reflect critical interactions at Site-1, Site-2, and the hydrophobic pocket, confirming the validity of the pharmacophore model and the strength of the screening approach. The docking study revealed that replacing the ethyl group on 1-Hydroxy-D-788-7 with a propyl group significantly improved the docking score from −10.2 kcal/mol to −11.25 kcal/mol, due to enhanced hydrophobic interactions with residues VAL128, VAL125, and VAL127. R-group enumeration using monocyclic, aromatic, and alkyl groups indicated that only small hydrophobic groups, such as an additional methyl, were tolerated within the hydrophobic pocket. The propyl analog maximized van der Waals contacts while avoiding steric clashes, highlighting the strict spatial constraints of the pocket. These findings suggested that future optimization could explore small alkyl or compact aromatic groups to further enhance binding affinity. Refer to Supplementary Figure S4 for a visual comparison of the parent compound and the designed analog, showing the R-group modifications.

**TABLE 3:** Parameters scores of the generated hypotheses.

No.	Hypothesis	PhaseHypoScore	Num matched	Selectivity score	Volume score	Vector score	Site score	Survival score
1	ADRR_1	0.956472	51	1.136750	0.561684	0.947172	0.898032	5.251209
2	ADRR_2	0.955290	51	1.136941	0.564364	0.945307	0.887346	5.241529
3	ADDRR_3	0.934114	44	1.506127	0.515424	0.985061	0.761866	5.411932
4	ADDDRR_6	0.932594	44	2.003748	0.480918	0.902195	0.599589	5.629902
5	ADDDRR_1	0.931054	37	1.987496	0.579493	0.918213	0.752505	5.805910

**TABLE 4:** Phase screen parameters for screening the 6 hits that fit the ADRR_1 hypothesis out of the 12 identified hits.

Name	PhaseScreenScore	Fitness score	Align score
Co-crystal ligand	2.30	2.31	0.25
Pyridindolol K2	2.03	2.03	0.56
Erythrin	1.42	1.42	0.78
Pyrronamycin B	1.32	1.32	0.94
Lampteroflavin	1.20	1.20	0.53
1-Hydroxy-D-788-7	0.95	0.95	1.11
Closthioamide	0.94	0.94	0.91

### 3.6 Quantum computational calculations

**FIGURE 10:**
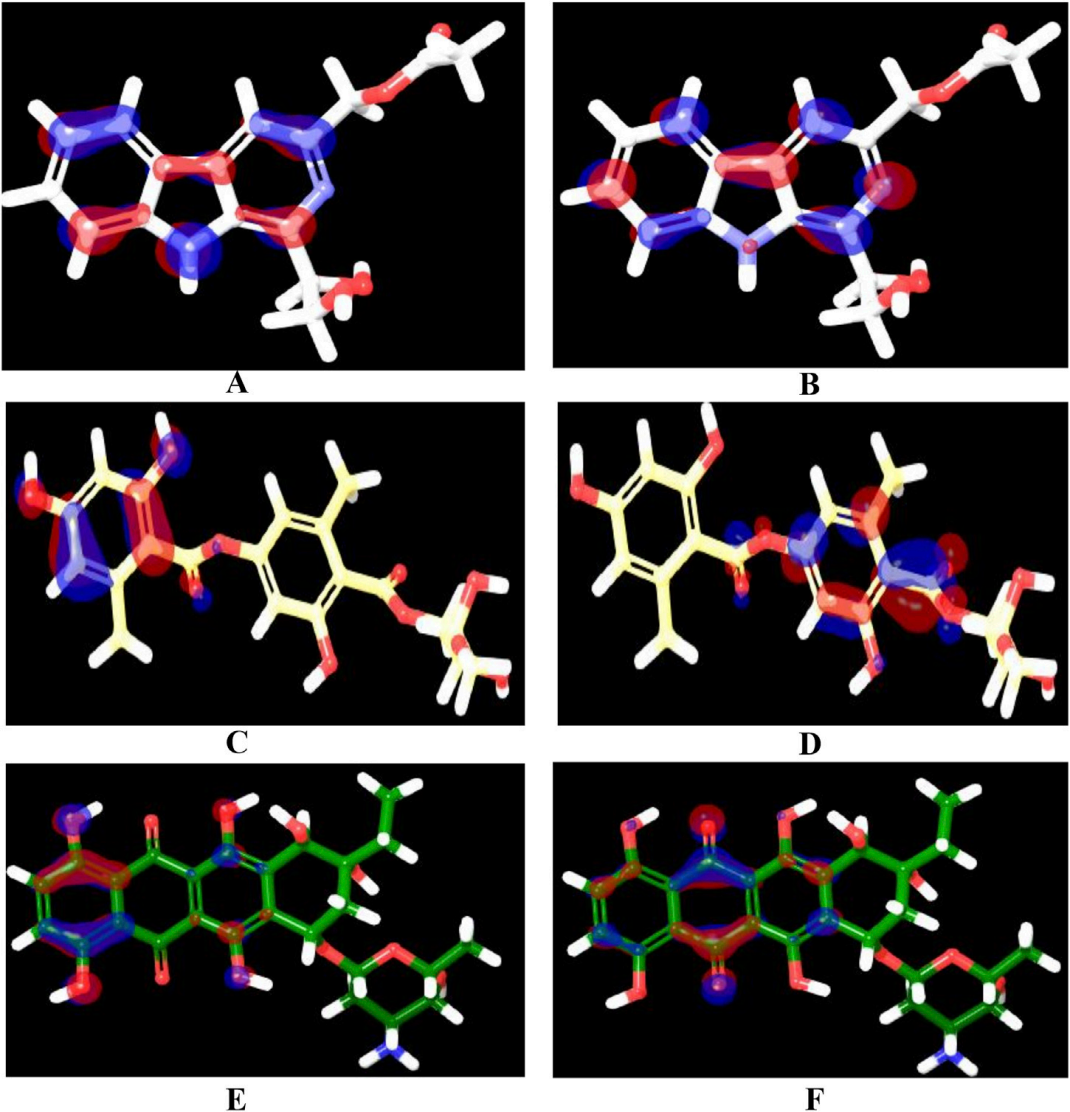
HOMO and LUMO orbitals of the three identified hits: Pyridindolol K2 [**(A)**: HOMO, **(B)** LUMO], Erythrin [**(C)**: HOMO, **(D)** LUMO], and 1-Hydroxy-D-788-7 [**(E)**: HOMO, **(F)** LUMO]. The orbitals were calculated using the B3LYP/6-31G**++ basis set with hybrid DFT. The red regions represent electron-dense areas, while the blue regions indicate electron-deficient areas. Created using the Maestro interface of Schrödinger Suite version 2023-1.

Over the last decade, Quantum mechanics (QM)-based methods have gathered an immense attention in the field of drug discovery as powerful and highly accurate tools for describing ligand-target interactions (Cavasotto et al., 2018). Among the most commonly used methods is the Density Functional Theory (DFT) method which has been proven to be useful, efficient, and sufficiently rigorous in various branches of Computer-aided Drug Design (CADD) (LaPointe and Weaver, 2007; Manathunga et al., 2022). In this study, we performed DFT at the B3LYP level to correlate the predicted affinity with the structural features, focusing mainly on the top three hits, which demonstrated high affinity, good ADMET properties, and matching to all chemical features of the generated pharmacophore model. The studied structural features included localization energies of lowest unoccupied molecular orbital (LUMO) and highest occupied molecular orbital (HOMO), along with the molecular electrostatic potential (MESP). These localization energies are known as frontier molecular orbitals (FMOs), and they play an important role in chemical stability, serving as an efficient tool for studying donor-acceptor interactions (Yele et al., 2021). The HOMO energy can determine the molecule’s tendency to contribute electrons to electrophilic centers, whereas LUMO can determine the capacity of a molecule to accept electrons from nucleophilic centers (Guezane-Lakoud et al., 2023). As shown in Table 5, the electronic properties of the three compounds, Pyridindolol K2, Erythrin, and 1-Hydroxy-D-788-7, revealed distinct differences in their reactivity and stability. 1-Hydroxy-D-788-7 features a complex framework, including a tetrahydrotetracene backbone with multiple hydroxyl groups and an ether linkage. The presence of multiple hydroxyl groups suggests the compound has a high potential for hydrogen bonding, which likely contributes to its high electronegativity (4.30 eV) and global electrophilicity index (2.80 eV). The smaller HOMO-LUMO gap (3.19 eV) and chemical softness (0.30 eV⁻^1^) indicate it is highly reactive, making it prone to electron donation or acceptance (Bouback et al., 2021; Ahmad et al., 2023). Its electron affinity (2.71 eV) aligns with its electrophilic nature, suggesting that 1-Hydroxy-D-788-7 is the most chemically reactive and capable of forming strong interactions with biological targets, especially through hydrogen bonds and electron interactions. On the other hand, Pyridindolol K2 contains an indole ring system, which is substituted with an amino group and a methoxy group. The relatively simple structure, with fewer hydroxyl groups compared to 1-Hydroxy-D-788-7, correlates with its more moderate HOMO-LUMO gap (4.4 eV) and chemical hardness (2.2 eV). This suggests that Pyridindolol K2 is more stable and less reactive than 1-Hydroxy-D-788-7, which is consistent with its moderate chemical softness (0.23 eV⁻^1^). The compound’s ability to accept electrons may be somewhat limited due to the absence of highly reactive functional groups like hydroxyl groups, but its electronegativity (3.5 eV) still indicates a potential for electron attraction in binding interactions. Further, Erythrin features a phenolic core with a trihydroxybutoxy side chain. The molecule’s high HOMO-LUMO gap (5.12 eV) and moderate chemical hardness (2.56 eV) suggest that it is relatively stable with limited reactivity, though it has the potential for moderate electron acceptance or donation. The compound’s electronegativity (3.64 eV) and chemical softness (0.20 eV) further indicate a moderate ability to interact with biological targets through hydrogen bonding or hydrophobic interactions. The structure’s hydroxyl groups suggest that Erythrin may interact with biological molecules through hydrogen bonding, but its stability and lower reactivity make it less prone to undergo significant electron transfer reactions compared to 1-Hydroxy-D-788-7. The distributions of HOMO and LUMO plots for three hits are shown in Figures 10A–F. It was observed that Pyridindolol K2 demonstrated a compact and rigid Pyridoindole core, which served as the primary structural motif for both its HOMO and LUMO orbitals (Figures 10A, B, respectively). The HOMO was distributed across the core, highlighting its electron-donating potential, while the LUMO was similarly localized, emphasizing the core’s role in electron acceptance. This dual functionality, combined with its ability to form H-bonds, makes Pyridindolol K2 a highly stable and strong binder to DNA gyrase B. Erythrin exhibited distinct separation in the structural motifs for its orbitals. The HOMO (Figure 10C) was localized on the resorcinol ring, reflecting its role as an electron donor due to its hydroxyl substituents, while the LUMO (Figure 10D) was concentrated on the phenolic ester ring, indicating its electron-accepting functionality. This separation of orbital distribution enabled Erythrin to effectively engage in donor-acceptor interactions and H-bonding with DNA gyrase B residues, enhancing its binding efficiency. Regarding 1-Hydroxy-D-788-7 presented a more complex structural motif for its orbitals. The HOMO (Figure 10E) was distributed across the dihydroxybenzene ring and dihydroxyhexane chain, highlighting its strong electron-donating capacity. In contrast, the LUMO (Figure 10F) was concentrated on the dioxocyclohexane ring, particularly around the two carbonyl groups, showcasing its electron-accepting role. This spatial separation between electron-dense and electron-deficient regions allowed 1-Hydroxy-D-788-7 to form multiple interaction types, including H-bonding, making it highly reactive and adaptable within the binding pocket.

**TABLE 5:** Quantum chemical reactivity descriptors of the top three hits.

Property	HOMO	LUMO	HLG	Electron affinity	Ionization potential	Chemical hardness	Chemical softness	Electronegativity	Global electrophilicity index
Pyridindolol K2	‒5.7	‒1.30	4.4	1.30	5.7	2.2	0.23	3.5	2.78
Erythrin	‒6.2	‒1.08	5.12	1.08	6.2	2.56	0.20	3.64	2.58
1-Hydroxy-D-788-7	‒5.9	‒2.71	3.19	2.71	5.9	1.65	0.30	4.30	2.80

### 3.7 MD simulations

**FIGURE 11:**
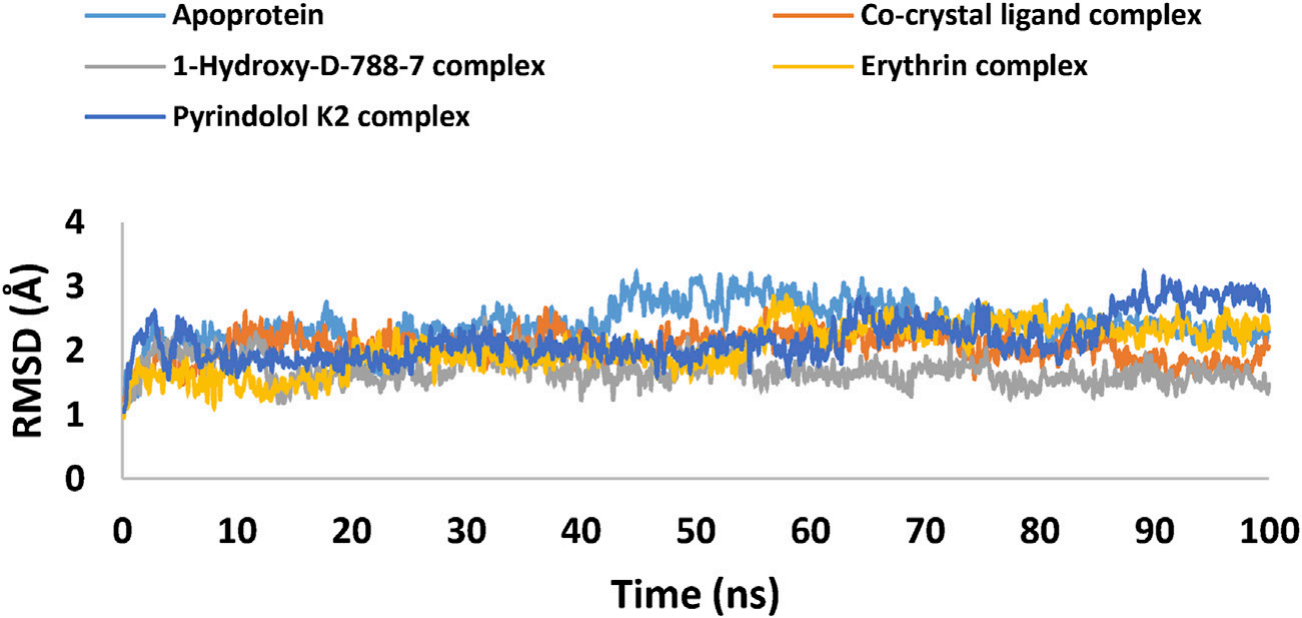
RMSD plot of the apoprotein, co-crystal ligand-protein complex, and the top three hits-protein complexes (1-Hydroxy-D-788-7, Erythrin, and Pyrindolol K2) over the 100 ns molecular dynamics simulation. Generated using Microsoft Excel.

**FIGURE 12:**
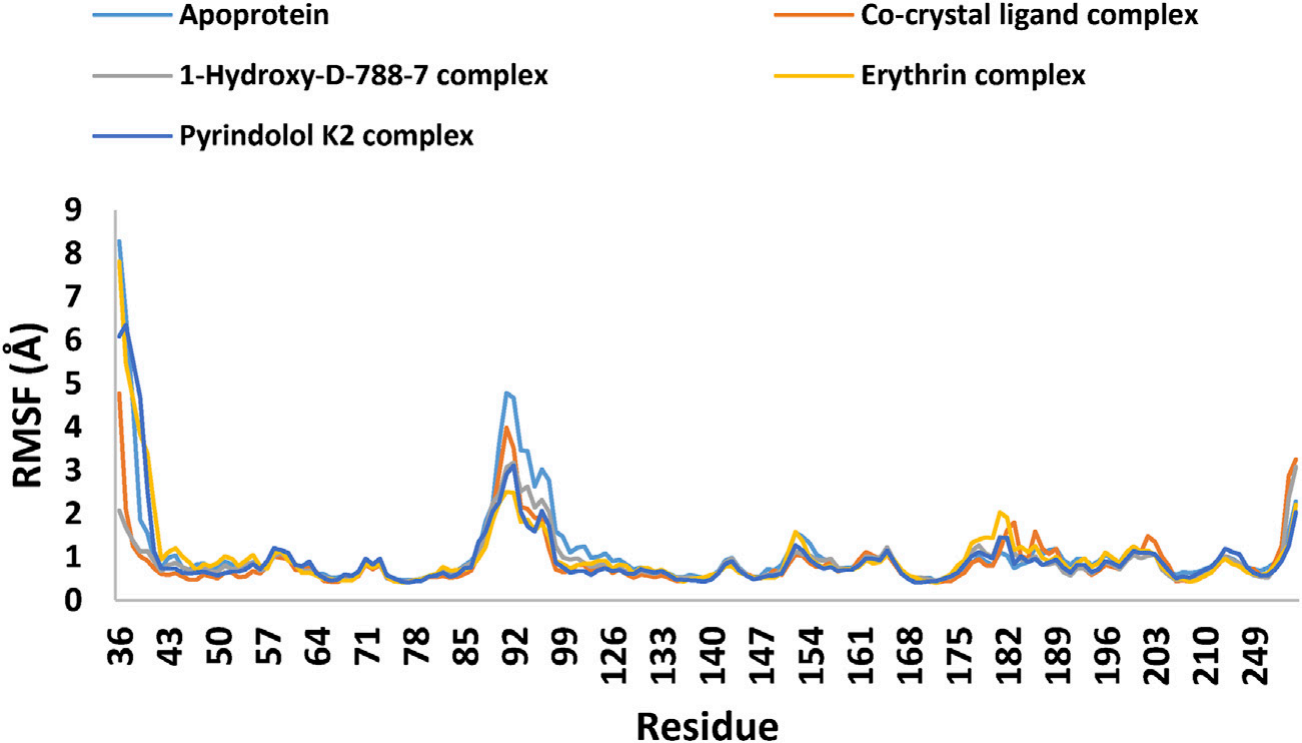
RMSF Plot generated through MDS trajectories, showing the Root Mean Square Fluctuation (RMSF) of the apoprotein, co-crystal ligand-protein complex, and the top three hits-protein complexes (1-Hydroxy-D-788-7, Erythrin, and Pyrindolol K2) over the 100 ns molecular dynamics simulation. Generated using Microsoft Excel.

**FIGURE 13:**
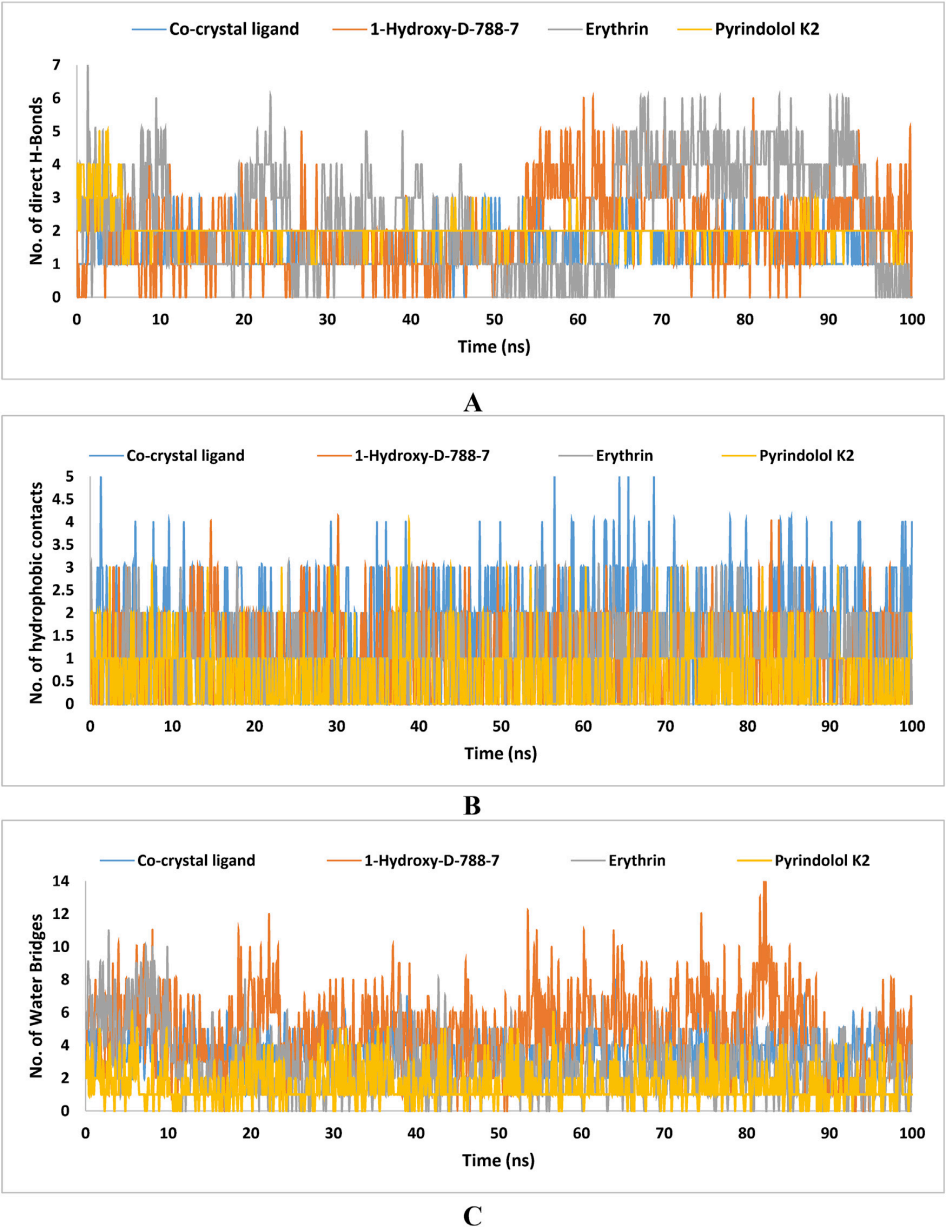
Number of **(A)** hydrogen bonds, **(B)** hydrophobic interactions, and **(C)** water-bridge interactions formed during the 100 ns MD simulation of the co-crystal ligand and the top three hits (1-Hydroxy-D-788-7, Erythrin, and Pyrindolol K2) with Mycobacterial DNA Gyrase B (PDB: 4B6C). The data were generated using Microsoft Excel.

With increased computer power and the development of novel force fields, MD simulations have proven increasingly valuable in providing vital insights into real-life molecular interactions events. In this regard, MD simulations can be a valuable tool in facilitating the early stages of modern drug discovery and development processes (Salo-Ahen et al., 2020). In the present study, MD simulations were performed to evaluate the stability and intermolecular interactions of Pyridindolol K2, Erythrin, and 1-Hydroxy-D-788-7, which demonstrated favorable binding affinity and a druggable ADMET profile. The enzyme-ligand complexes of those hits, the co-crystal ligand complex, alongside the unbound protein, were simulated over 100 ns using the Desmond package with the SPC solvation model. Certain parameters such as RMSD, root mean square fluctuation (RMSF), and intermolecular interactions were utilized to reveal the molecular details of ligand-protein simulations (Mohankumar et al., 2020). RMSD is a key measure used to evaluate the overall stability of the ligand-protein complex during the simulation. As shown in Table 6; Figure 11, the RMSD trajectories for each system revealed key insights into the stability of the ligand binding throughout the 100 ns simulation. The RMSD of the co-crystal ligand remained steady at approximately 2.1 Å after an initial stabilization phase within the first 10 ns, demonstrating its strong and stable interaction with the protein. This suggests that the co-crystal ligand maintained consistent binding throughout the simulation, confirming that it forms a stable complex with the protein. The co-crystal ligand’s RMSD remained unchanged for the remainder of the simulation, indicating no significant conformational changes or instability. Among the hits, 1-Hydroxy-D-788-7 displayed the most stable binding, with an RMSD of 1.7 Å, which quickly stabilized after the first 10 ns. The RMSD profile for 1-Hydroxy-D-788-7 remained consistent over the entire simulation, with only minor fluctuations in the initial phase. Importantly, after 30 ns, this ligand showed superior stability compared to the co-crystal ligand, maintaining a more stable RMSD trajectory, indicating more reliable binding interactions with the protein. The low RMSD values indicate that 1-Hydroxy-D-788-7 interacts with the protein in a highly stable manner, providing strong evidence of its potential as a promising hit. The stability of 1-Hydroxy-D-788-7, indicated by its low RMSD (1.7 Å), strongly correlated with its consistent intermolecular interactions with the protein. The low RMSD value suggested that the ligand maintained a stable binding conformation, with minimal structural deviations throughout the simulation. This stability was further supported by the ligand’s favorable hydrogen bonding and water-bridge contacts, which likely contributed to the rigid binding at the active site. The consistent interactions, especially in the later stages of the simulation, indicated that 1-Hydroxy-D-788-7 formed strong, reliable interactions with key residues, reducing protein flexibility and enhancing its stability. This stable binding profile reinforced its potential as a promising drug candidate. The strong binding interactions, coupled with minimal fluctuation, implied that 1-Hydroxy-D-788-7 could effectively inhibit the target protein. Both Pyrindolol K2 and Erythrin exhibited slightly higher RMSD values (around 2.0 Å) compared to 1-Hydroxy-D-788-7. Although these ligands remained relatively stable throughout the simulation, their higher RMSD values indicated that they may experience slightly more conformational flexibility or weaker interactions with the protein, suggesting they are somewhat less stable than 1-Hydroxy-D-788-7. However, both ligands still displayed acceptable stability and could be considered as secondary candidates for further evaluation. In comparison to Amorim et al. (2022) and Arevalo and Amorim (2022), who reported that the Gyr B-ATD complex, involving an anthraquinone-like ligand similar to 1-Hydroxy-D-788-7, showed lower RMSD values (around 2 Å) than the apo form, the results in this study demonstrated that 1-Hydroxy-D-788-7 formed a more stable complex with the protein, with an RMSD value of 1.7 Å, outperforming the anthraquinone-like ligand in Amorim et al.’s study and PQNN in Amorim and Arévalo study. On the other hand, Erythrin and Pyrindolol K2 exhibited similar RMSD values (around 2.1 Å and 2.2 Å, respectively), indicating comparable stability but with slightly more fluctuation than 1-Hydroxy-D-788-7. Furthermore, the RMSD values for the top three hits are comparable to or better than those reported in the Islam and Pillay study (Islam and Pillay, 2017), which also used the same PDB ID **(4B6C)** for DNA Gyrase B. With RMSD values ranging from 1.70 to 2.1 Å, the identified hits in this study demonstrate slightly more stable binding compared to the study’s range of 2.02–3.27 Å, suggesting an enhanced binding affinity for our selected compounds. The apoprotein demonstrated significant instability throughout the simulation, with RMSD values fluctuating widely and averaging 2.4 Å, underscoring the dynamic and flexible nature of the protein in the absence of a stabilizing ligand. The higher RMSD value for the apoprotein further highlighted the critical role of ligand binding in maintaining protein structure stability, with the absence of a ligand leading to greater conformational variability. Thus, the top three hits from the virtual screening, as shown in Table 7, demonstrated superior performance metrics compared to literature-reported compounds. Specifically, 1-Hydroxy-D-788-7, Erythrin, and Pyrindolol K2 achieved docking scores ranging from −9.492 to −10.253, reflecting stronger binding affinities to the target protein when compared to literature compounds, which exhibited significantly weaker scores (−3.891 to −5.123). Their binding free energies, calculated between −60.37 and −65.68 kcal/mol, suggested greater thermodynamic stability, highlighting their ability to form energetically favorable interactions with the binding pocket. Furthermore, the top hits showed lower RMSD values (1.7–2.2 Å), indicating better alignment of their conformations with the reference structure during molecular dynamics simulations. In addition, the lower RMSF values (0.9–1.0 Å) demonstrated reduced atomic fluctuations, suggesting enhanced stability of these compounds within the dynamic protein-ligand complex. In contrast, literature-reported compounds displayed higher RMSD and RMSF values, signifying weaker structural stability and flexibility during simulation. These findings collectively underscore the superior binding affinity, stability, and structural alignment of the identified top hits, making them highly promising candidates for further optimization and experimental validation in the context of targeting *M. tuberculosis* gyrase B. Overall, the RMSD data revealed that 1-Hydroxy-D-788-7 provided the most stable binding interactions with the protein, outperforming the co-crystal ligand and the anthraquinone-like ligand from Amorim et al. (2022). Erythrin and Pyrindolol K2 demonstrated relatively less stability compared to 1-Hydroxy-D-788-7, but still showed consistent binding interactions, suggesting that both ligands may require further optimization for more robust interactions. The RMSD graph of the unbound protein, the co-crystallized ligand, and the top three hits (Table 6; Figure 11) demonstrated that the 1-Hydroxy-D-788-7 complex achieved greater stability and equilibration compared to the co-crystal ligand complex throughout the simulation period. The C-alpha atoms of the protein-1-Hydroxy-D-788-7 complex exhibited fluctuations ranging from 1.1 Å to 2.5 Å during the entire simulation, whereas the protein-co-crystal ligand complex fluctuated between 1.1 Å and 2.7 Å over the 100 ns run. In contrast, the unbound protein displayed more pronounced fluctuations, ranging from 1.1 Å to 3.2 Å throughout the simulation. Figure 11 highlighted that the top three hits binding to the mycobacterial DNA gyrase B conferred dynamic equilibration and maintained a steady interaction profile with the protein over the 100 ns simulation. For gain more insights into the dynamic behaviors of each amino acid residue during the simulation run, the RMSF was monitored. It is defined as the displacement of a specific atom or group of atoms relative to the reference structure, averaged across the number of atoms (Ravikumar et al., 2023). As depicted in Table 6 and The RMSF graph (Figure 12), the co-crystal ligand and 1-Hydroxy-D-788-7 induced the least residue flexibility, with average P-RMSF values of 0.9 Å, indicating strong interactions that minimized protein flexibility. Erythrin and Pyrindolol K2 caused slightly higher residue fluctuations (1.0 Å), suggesting comparable binding strength. The apoprotein displayed relatively higher flexibility, with an average RMSF of 1.1 Å and peaks reaching 8.3 Å, reflecting substantial conformational changes without ligand stabilization. Interestingly, the loop containing the residues responsible for ligand interactions showed less flexibility (below 1 Å) for the 3 hits and the co-crystal ligand, as indicated by the RMSF data. This suggested that these ligands stabilized the protein and reduced the flexibility of the binding site residues, in contrast to the apo form, which showed greater flexibility. This further emphasized the stabilizing role of ligand binding in maintaining a more rigid protein conformation. This is further substantiated by the fact that the RMSF values for the top three hits ranged from 0.9 to 1.0 Å, indicating significantly lower fluctuations in the protein backbone compared to the study by Islam and Pillay (Islam and Pillay, 2017), where the RMSF values ranged from 2.3 to 3.8 Å. Further, the individual RMSF values for residues in the active site were examined, with a particular focus on the critical residues Val49, Glu56, Asp79, Arg82, Gly83, Val123, Val125, Val128, and Arg141. These residues demonstrated reduced flexibility when bound to the top three hits, showcasing stability that was either comparable to or better than that observed for the co-crystallized ligand. Notably, the most critical residue, Asp79, was stabilized by Pyrindolol K2 with an RMSF value of 0.45 Å, which was superior to the stabilization observed for the co-crystallized ligand (0.47 Å) and the unbound protein (0.49 Å). This finding underscores the significant role of the top three hits in conferring enhanced stability to the active site residues, thereby highlighting their potential effectiveness in binding and stabilizing the protein’s active site. These results suggested that the top three hits in the present study induced more stable conformations of DNA Gyrase B, with reduced flexibility, thereby further supporting their enhanced binding affinity and potential as promising inhibitors. The RMSF analysis of 1-Hydroxy-D-788-7 revealed a significant reduction in flexibility, particularly at the active site, where the RMSF was lower than the apo-protein. This suggested that the ligand binding effectively stabilized the protein, limiting fluctuations in key residues involved in catalysis. Compared to the unbound protein, the ligand-bound system exhibited a more rigid structure, with minimal changes in the active site residues, indicating stronger interactions. The RMSF values for the ligand-protein complex were consistent, showing that 1-Hydroxy-D-788-7 maintained stable interactions throughout the simulation. Furthermore, Table 8 presents a comprehensive analysis of the average protein RMSF (P-RMSF) values across different regions of the GyrB complex, evaluated in both Apo and ligand-bound forms. Three primary regions were analyzed: the Active Site Region, the ATPase Domain, and the Toprim Domain. Each of these regions is essential for the protein’s functionality. The Active Site Region is primarily responsible for DNA binding and catalysis, the ATPase Domain plays a key role in energy hydrolysis, and the Toprim Domain aids in the interaction between the protein and DNA. Together, these regions ensure the proper function and stability of the GyrB complex (Papillon et al., 2013). In the Active Site region, the Apo form exhibited the highest flexibility (P-RMSF = 0.99 Å), indicating greater structural mobility in the unbound state. However, upon ligand binding, particularly with the co-crystal ligand, this flexibility was notably reduced, with the lowest P-RMSF value observed (0.67 Å), suggesting enhanced stability in the region when the ligand is bound. In contrast, the ATPase domain exhibited consistent flexibility between the Apo form and the co-crystal ligand (P-RMSF = 1.42 Å), reflecting a relatively stable structural conformation in both conditions. Ligands such as 1-Hydroxy-D-788-7, Erythrin, and Pyrindolol K2 led to a reduction in flexibility within this domain, with 1-Hydroxy-D-788-7 providing the most substantial stabilization (P-RMSF = 0.96 Å). This decrease in flexibility underscores the potential for these ligands to induce more rigid, functionally relevant conformations. The Toprim domain displayed consistent stability across all conditions, with P-RMSF values ranging from 0.78 Å to 0.85 Å, highlighting its inherent stability regardless of the presence of ligands. Notably, the Apo form exhibited the highest flexibility in this region, whereas Pyrindolol K2 induced the greatest stabilization (P-RMSF = 0.78 Å), demonstrating its ability to stabilize this domain effectively. Overall, ligand binding was shown to generally stabilize the protein, particularly within the Active Site region, where the co-crystal ligand had the most pronounced effect. These results indicate that 1-Hydroxy-D-788-7 and Pyrindolol K2 played critical roles in stabilizing the ATPase and Toprim domains, respectively. These findings offer valuable insights into the molecular interactions of ligands with the GyrB complex and provide a foundation for prioritizing potential ligands for future hits identification, particularly those with the capacity to reduce flexibility and enhance protein stability. To gain deeper insights into the binding interactions of the top three compounds with mycobacterial DNA Gyrase B during the simulation, an extensive analysis of ligand-residue interactions was performed. The results are illustrated in Figure 13. As summarized in Table 6; Figure 13A, hydrogen bond analysis highlighted that 1-Hydroxy-D-788-7 exhibited the strongest interactions, averaging 2.5 hydrogen bonds and reaching a maximum of 7, outperforming the co-crystal ligand (average: 2.0; maximum: 6). Erythrin demonstrated comparable average hydrogen bonding to the co-crystal ligand (2.0) but showed fewer maxima (5). In contrast, Pyrindolol K2 displayed weaker and less consistent interactions (average: 1.4; maximum: 3). Notably, Pyrindolol K2 retained a hydrogen bond with the critical residue ASP79 for 98% of the simulation duration, closely mirroring the behavior of the co-crystal ligand. Hydrophobic contact analysis (Table 6; Figure 13B) revealed that the co-crystal ligand had the strongest interactions (average: 1.7), followed by 1-Hydroxy-D-788-7 (1.1) and Erythrin (1.0), while Pyrindolol K2 contributed minimally (0.6). Regarding Pi-cation interactions (Table 6), the co-crystal ligand exhibited the most significant contacts (average: 0.6), marginally exceeding Erythrin (average: 0.5). 1-Hydroxy-D-788-7 showed moderate interactions (average: 0.3; maximum: 1.0), whereas Pyrindolol K2 lacked notable Pi-cation interactions (average: 0.0). Analysis of water-bridge contacts (Table 6; Figure 13C) indicated that 1-Hydroxy-D-788-7 exhibited the highest levels of interaction, with an average of 4.8 and a peak of 14, surpassing the co-crystal ligand (average: 3.7; maximum: 8.0). Erythrin and Pyrindolol K2 displayed lower water-bridge interactions, reflecting reduced stability in their binding modes. Supplementary Figure S5 detailed protein-ligand contact histograms for a comprehensive view of the interactions. Overall, 1-Hydroxy-D-788-7 emerged as the most promising candidate, demonstrating superior or comparable performance to the co-crystal ligand across key metrics, including binding interactions, stability, and flexibility, thus establishing itself as a robust hit. Erythrin also showed significant promise as an alternative candidate, with consistent binding interactions and stability throughout the simulation. While Pyrindolol K2 exhibited relatively weaker interactions, its sustained binding to the critical residue ASP79 underscores its potential for further exploration. Importantly, all three compounds require chemical optimization to enhance pharmacokinetic properties and strengthen interactions within the enzyme’s hydrophobic pocket, a crucial step to improving their binding efficacy and therapeutic potential. Such advancements would address current limitations and foster the development of highly potent enzyme inhibitors.

**TABLE 6:** RMSD, RMSF, and ligand-protein contacts (H-bonds, water bridges, hydrophobic, and Pi-cation interactions) for apo form, Co-crystal ligand, and Top 3 hits (1-Hydroxy-D-788-7, Erythrin, and Pyrindolol K2) over a 100 ns MD simulation.

Gyr B complex	Apo	Co-crystal ligand	1-Hydroxy-D-788-7	Erythrin	Pyrindolol K2
PL-RMSD (Å)					
Average	2.4	2.1	1.7	2.1	2.2
Maximum	3.2	2.7	2.5	2.9	3.2
Minimum	1.1	1.1	1.1	1.0	1.0
P-RMSF (Å)					
Average	1.1	0.9	0.9	1.0	1.0
Maximum	8.3	4.8	3.2	7.8	6.4
Minimum	0.4	0.4	0.4	0.4	0.4
H-bond contacts					
Average	-	1.4	2.0	2.5	2.0
Maximum	-	3.0	6.0	7.0	5.0
Minimum	-	0.0	0.0	0.0	1.0
Hydrophobic contacts					
Average	-	1.7	1.1	1.0	0.6
Maximum	-	5.0	4.0	3.0	4.0
Minimum	-	0.0	0.0	0.0	0.0
Pi-cation contacts					
Average	-	0.6	0.3	0.5	0.0
Maximum	-	1.0	1.0	1.0	0.0
Minimum	-	0.0	0.0	0.0	0.0
Water-bridge contacts					
Average	-	3.7	4.8	2.7	1.6
Maximum	-	8.0	14.0	11.0	6.0
Minimum	-	1.0	0.0	0.0	0.0

**TABLE 7:** Comparative analysis of study compounds vs literature-reported compounds.

Hits	Virtual screening parameters		Average MD parameters		References
	Docking score	Binding free energy	RMSD (Å)	RMSF (Å)	
1-Hydroxy-D-788-7	‒10.253	‒60.37	1.7	0.9	Hits identified from the present study
Erythrin	‒9.492	‒65.68	2.1	1.0	
Pyrindolol K2	‒9.590	‒64.51	2.2	1.0	
Literature reported compounds					
ATD	‒5.123	‒35.19	2	4	Amorim et al. (2022)
PQPNN	‒3.891	‒20.74	3.1	>1	Arevalo and Amorim (2022)
D5	‒4.225	‒44.59	2.02 to 3.27	3.8	Islam and Pillay (2017)

**TABLE 8:** Protein flexibility analysis of GyrB complex in Apo and ligand-bound states across active site, ATPase, and Toprim regions.

Gyr B complex	Average P-RMSF (Å) per protein region		
	Active site region	ATPase domain	Toprim domain
Apo	0.99	1.42	0.85
Co-crystal ligand	0.67	1.42	0.82
1-Hydroxy-D-788-7	0.82	0.96	0.80
Erythrin	0.79	1.02	0.84
Pyrindolol K2	0.72	1.2	0.78

### 3.8 Study limitations and future perspective

In the present study an innovative approach was explored to identify new mycobacterial DNA gyrase B inhibitors from microbial-derived natural products database. These products have long been known for their potential bioactivity against infectious diseases. Implementation of multiple computational tools such as, target-based virtual screening, XP molecular docking, binding free energy calculations, ADMET profiling, pharmacophore modeling, quantum mechanical calculations and MD simulations, the identified hits (1-Hydroxy-D-788-7, Erythrin, and Pyrindolol K2) demonstrated properties that were better than those of the existing natural inhibitors of the catalytic activity of mycobacterial DNA gyrase. Despite these promising computational findings, significant limitations need to be addressed. Of particular importance is the accuracy of the computational methods which heavily dependent on the availability of the structural data which may not fully reflect the intricacies of the biological systems. For instance, computer-based simulations could not predict the potential *in vivo* metabolic reactions and off-target effects resulting in uncertainty regarding the therapeutic efficacy and safety of the investigated hits. Further, molecular docking simulations involves assumptions about the flexibility of both proteins and ligands. Instead, it assumes a static active site, which may not precisely capture the dynamic behavior of proteins that can adopt several conformations, potentially affecting the reliability of the results. Accordingly, the calculated docking scores which predict the binding potential of the investigated hits do not consistently correlate with the experimental binding affinity values. To tackle this issue, binding free energy calculations were employed to enhance the accuracy of estimating the affinity of our investigated hits. In this regard, two main factors were incorporated, the solvent effects and the structural flexibility of both protein and the investigated hits. Furthermore, MD simulations, which provide a dynamic view, were conducted to assess the stability of the identified hits complexes as potent mycobacterial DNA gyrase inhibitors. Nevertheless, MD studies are also constrained by the relatively short time scales commonly simulated. To accurately reproduce thermodynamic properties and fully elucidate all binding site configurations important for drug design, it is necessary to explore all potential conformational states of the protein within the simulation. Unfortunately, several biological processes, such as receptor conformational changes relevant to drug binding, occur over much longer time scales exceeding those suitable for simulation. Given the above-mentioned shortcomings, wet-lab validation is essential to confirm the potential of our identified hits, as effective inhibitors targeting mycobacterial DNA gyrase B. Experimental validation in the process of drug discovery comprises both *in vitro* and *in vivo* assays. In this context, *in vitro* testing involving enzyme inhibition assays and cell-based tests, are highly recommended to validate the potential of the identified hits as effective inhibitors of mycobacterial DNA gyrase B. Further, *in vivo* studies will be crucial for evaluating its ADMET properties as well as its therapeutic potential. Overall, the computational approach used in this study provides valuable insights, but its limitations include the absence of experimental validation for the identified hits. Future research should focus on conducting thorough *in vitro* and *in vivo* evaluations of these compounds to improve their practical application and translational potential.

## 4 Conclusion

This study identified 1-Hydroxy-D-788-7, an anthracycline derivative, as a promising hit compound for targeting Mycobacterial DNA gyrase, a key enzyme in *M. tuberculosis*. Using computational tools like docking, ADME-T profiling, and molecular dynamics simulations, 12 potential inhibitors were screened, with 1-Hydroxy-D-788-7, Erythrin, and Pyrindolol K2 emerging as top candidates. 1-Hydroxy-D-788-7 showed the strongest binding affinity and stable interactions with DNA gyrase B. The results highlight its potential as an anti-tuberculosis agent, particularly against drug-resistant strains. Further experimental validation is needed to confirm its efficacy as a novel treatment for MDR-TB and XDR-TB.

## Data Availability

The original contributions presented in the study are included in the article/Supplementary Material, further inquiries can be directed to the corresponding authors.

## References

[B1] Agrawal A. Roué M. Spitzfaden C. Petrella S. Aubry A. Hann M. (2013). *Mycobacterium tuberculosis* DNA gyrase ATPase domain structures suggest a dissociative mechanism that explains how ATP hydrolysis is coupled to domain motion. Biochem. J. 456 (2), 263–273. 10.1042/bj20130538 24015710

[B2] Ahmad S. Gupta D. Ahmed T. Islam A. (2023). Designing of new tetrahydro-β-carboline-based ABCG2 inhibitors using 3D-QSAR, molecular docking, and DFT tools. J. Biomol. Struct. Dyn. 41, 14016–14027. 10.1080/07391102.2023.2176361 36752362

[B3] Alghamdi A. Abouzied A. S. Alamri A. Anwar S. Ansari M. Khadra I. (2023). Synthesis, molecular docking, and dynamic simulation targeting main protease (mpro) of new, thiazole clubbed pyridine scaffolds as potential COVID-19 inhibitors. Curr. Issues Mol. Biol. 45 (2), 1422–1442. 10.3390/cimb45020093 36826038 PMC9955078

[B4] Allue-Guardia A. Garcia J. I. Torrelles J. B. (2021). Evolution of drug-resistant *Mycobacterium tuberculosis* strains and their adaptation to the human lung environment. Front. Microbiol. 12, 612675. 10.3389/fmicb.2021.612675 33613483 PMC7889510

[B5] Almansour N. M. Allemailem K. S. Abd El Aty A. A. Ismail E. I. F. Ibrahim M. A. A. (2023). *In silico* mining of natural products Atlas (NPAtlas) database for identifying effective bcl-2 inhibitors: molecular docking, molecular dynamics, and pharmacokinetics characteristics. Molecules 28 (2), 783. 10.3390/molecules28020783 36677841 PMC9864825

[B6] Amorim J. C. Cabrera Bermeo A. E. Vasquez Urgiles V. E. Martinez Leon M. R. Carpio Arevalo J. M. (2022). An in-silico evaluation of anthraquinones as potential inhibitors of DNA gyrase B of *Mycobacterium tuberculosis* . Microorganisms 10 (12), 2434. 10.3390/microorganisms10122434 36557686 PMC9783175

[B7] Arevalo J. M. C. Amorim J. C. (2022). Virtual screening, optimization and molecular dynamics analyses highlighting a pyrrolo[1,2-a]quinazoline derivative as a potential inhibitor of DNA gyrase B of *Mycobacterium tuberculosis* . Sci. Rep. 12 (1), 4742. 10.1038/s41598-022-08359-x 35304513 PMC8933452

[B8] Asai A. Sakai Y. Ogawa H. Yamashita Y. Kakita S. Ochiai K. (2000). Pyrronamycin A and B, novel antitumor antibiotics containing pyrrole-amide repeating unit, produced by Streptomyces sp. J. Antibiotics 53 (1), 66–69. 10.7164/antibiotics.53.66 10724011

[B9] Atanasov A. G. Zotchev S. B. Dirsch V. M. International Natural Product Sciences T. Supuran C. T. Rollinger J. M. (2021). Natural products in drug discovery: advances and opportunities. Nat. Rev. Drug Discov. 20 (3), 200–216. 10.1038/s41573-020-00114-z 33510482 PMC7841765

[B10] Aubry A. Pan X. S. Fisher L. M. Jarlier V. Cambau E. (2004). *Mycobacterium tuberculosis* DNA gyrase: interaction with quinolones and correlation with antimycobacterial drug activity. Antimicrob. Agents Chemother. 48 (4), 1281–1288. 10.1128/AAC.48.4.1281-1288.2004 15047530 PMC375300

[B11] Avalos E. Catanzaro D. Catanzaro A. Ganiats T. Brodine S. Alcaraz J. (2015). Frequency and geographic distribution of gyrA and gyrB mutations associated with fluoroquinolone resistance in clinical *Mycobacterium tuberculosis* isolates: a systematic review. PLoS One 10 (3), e0120470. 10.1371/journal.pone.0120470 25816236 PMC4376704

[B12] Azam F. Eid E. E. Almutairi A. (2021). Targeting SARS-CoV-2 main protease by teicoplanin: a mechanistic insight by docking, MM/GBSA and molecular dynamics simulation. J. Mol. Struct. 1246, 131124. 10.1016/j.molstruc.2021.131124 34305175 PMC8286173

[B13] Bagcchi S. (2023). WHO's global tuberculosis report 2022. Lancet Microbe 4 (1), e20. 10.1016/S2666-5247(22)00359-7 36521512

[B14] Balasubramani G. L. Rajput R. Gupta M. Dahiya P. Thakur J. K. Bhatnagar R. (2020). Structure-based drug repurposing to inhibit the DNA gyrase of *Mycobacterium tuberculosis* . Biochem. J. 477 (21), 4167–4190. 10.1042/bcj20200462 33030198

[B15] Banke A. Fosbol E. L. Moller J. E. Gislason G. H. Andersen M. Bernsdorf M. (2018). Long-term effect of epirubicin on incidence of heart failure in women with breast cancer: insight from a randomized clinical trial. Eur. J. Heart Fail 20 (10), 1447–1453. 10.1002/ejhf.1168 29493047

[B16] Barth S. A. Preussger D. Pietschmann J. Fessler A. T. Heller M. Herbst W. (2024). *In vitro* antibacterial activity of microbial natural products against bacterial pathogens of veterinary and zoonotic relevance. Antibiot. (Basel) 13 (2), 135. 10.3390/antibiotics13020135 PMC10886079 38391521

[B17] Batool M. Ahmad B. Choi S. (2019). A structure-based drug discovery paradigm. Int. J. Mol. Sci. 20 (11), 2783. 10.3390/ijms20112783 31174387 PMC6601033

[B18] Bochevarov A. D. Harder E. Hughes T. F. Greenwood J. R. Braden D. A. Philipp D. M. (2013). Jaguar: a high‐performance quantum chemistry software program with strengths in life and materials sciences. Int. J. Quantum Chem. 113 (18), 2110–2142. 10.1002/qua.24481

[B19] Bouback T. A. Pokhrel S. Albeshri A. Aljohani A. M. Samad A. Alam R. (2021). Pharmacophore-based virtual screening, quantum mechanics calculations, and molecular dynamics simulation approaches identified potential natural antiviral drug candidates against MERS-CoV S1-NTD. Molecules 26 (16), 4961. 10.3390/molecules26164961 34443556 PMC8401589

[B20] Caldwell G. W. Yan Z. Masucci J. A. Hageman W. Leo G. Ritchie D. M. (2003). Applied pharmacokinetics in drug development: an overview of drug discovery. Pharm. Dev. Regul. 1, 117–132. 10.1007/bf03257371

[B21] Cao F. Meng Z. H. Wang P. Luo D. Q. Zhu H. J. (2020). Dipleosporalones A and B, dimeric azaphilones from a marine-derived Pleosporales sp. fungus. J. Nat. Prod. 83 (4), 1283–1287. 10.1021/acs.jnatprod.0c00132 32243144

[B22] Cavasotto C. N. Adler N. S. Aucar M. G. (2018). Quantum chemical approaches in structure-based virtual screening and lead optimization. Front. Chem. 6, 188. 10.3389/fchem.2018.00188 29896472 PMC5986912

[B23] Chang Y. Hawkins B. A. Du J. J. Groundwater P. W. Hibbs D. E. Lai F. (2022). A guide to *in silico* drug design. Pharmaceutics 15 (1), 49. 10.3390/pharmaceutics15010049 36678678 PMC9867171

[B24] Chiriac A. I. Kloss F. Kramer J. Vuong C. Hertweck C. Sahl H. G. (2015). Mode of action of closthioamide: the first member of the polythioamide class of bacterial DNA gyrase inhibitors. J. Antimicrob. Chemother. 70 (9), 2576–2588. 10.1093/jac/dkv161 26174721

[B25] Chopra S. Matsuyama K. Tran T. Malerich J. P. Wan B. Franzblau S. G. (2012). Evaluation of gyrase B as a drug target in *Mycobacterium tuberculosis* . J. Antimicrob. Chemother. 67 (2), 415–421. 10.1093/jac/dkr449 22052686 PMC3254195

[B26] De Vivo M. Masetti M. Bottegoni G. Cavalli A. (2016). Role of molecular dynamics and related methods in drug discovery. J. Med. Chem. 59 (9), 4035–4061. 10.1021/acs.jmedchem.5b01684 26807648

[B27] Dheda K. Gumbo T. Maartens G. Dooley K. E. McNerney R. Murray M. (2017). The epidemiology, pathogenesis, transmission, diagnosis, and management of multidrug-resistant, extensively drug-resistant, and incurable tuberculosis. Lancet Respir. Med. 5, 291–360. 10.1016/s2213-2600(17)30079-6 28344011

[B28] Dimise E. J. Widboom P. F. Bruner S. D. (2008). Structure elucidation and biosynthesis of fuscachelins, peptide siderophores from the moderate thermophile Thermobifida fusca. Proc. Natl. Acad. Sci. U. S. A. 105 (40), 15311–15316. 10.1073/pnas.0805451105 18832174 PMC2563069

[B29] Divyashri G. Krishna Murthy T. P. Sundareshan S. Kamath P. Murahari M. Saraswathy G. R. (2020). *In silico* approach towards the identification of potential inhibitors from Curcuma amada Roxb against *H. pylori*: ADMET screening and molecular docking studies. BioImpacts 11 (2), 119–127. 10.34172/bi.2021.19 33842282 PMC8022237

[B30] Dixon S. L. Smondyrev A. M. Knoll E. H. Rao S. N. Shaw D. E. Friesner R. A. (2006a). PHASE: a new engine for pharmacophore perception, 3D QSAR model development, and 3D database screening: 1. Methodology and preliminary results. J. computer-aided Mol. Des. 20, 647–671. 10.1007/s10822-006-9087-6 17124629

[B31] Dixon S. L. Smondyrev A. M. Rao S. N. (2006b). PHASE: a novel approach to pharmacophore modeling and 3D database searching. Chem. Biol. and drug Des. 67 (5), 370–372. 10.1111/j.1747-0285.2006.00384.x 16784462

[B32] Dookie N. Rambaran S. Padayatchi N. Mahomed S. Naidoo K. (2018). Evolution of drug resistance in *Mycobacterium tuberculosis*: a review on the molecular determinants of resistance and implications for personalized care. J. Antimicrob. Chemother. 73 (5), 1138–1151. 10.1093/jac/dkx506 29360989 PMC5909630

[B33] Friesner R. A. Banks J. L. Murphy R. B. Halgren T. A. Klicic J. J. Mainz D. T. (2004). Glide: a new approach for rapid, accurate docking and scoring. 1. Method and assessment of docking accuracy. J. Med. Chem. 47 (7), 1739–1749. 10.1021/jm0306430 15027865

[B34] Friesner R. A. Murphy R. B. Repasky M. P. Frye L. L. Greenwood J. R. Halgren T. A. (2006). Extra precision glide: docking and scoring incorporating a model of hydrophobic enclosure for protein− ligand complexes. J. Med. Chem. 49 (21), 6177–6196. 10.1021/jm051256o 17034125

[B35] Fu G. Wu J. Liu W. Zhu D. Hu Y. Deng J. (2009). Crystal structure of DNA gyrase B' domain sheds lights on the mechanism for T-segment navigation. Nucleic Acids Res. 37 (17), 5908–5916. 10.1093/nar/gkp586 19596812 PMC2761264

[B36] Germe T. R. Bush N. G. Baskerville V. M. Saman D. Benesch J. L. Maxwell A. (2024). Rapid, DNA-induced interface swapping by DNA gyrase. Elife 12, RP86722. 10.7554/eLife.86722 38856655 PMC11164529

[B37] Guex N. Peitsch M. C. Schwede T. (2009). Automated comparative protein structure modeling with SWISS-MODEL and Swiss-PdbViewer: a historical perspective. Electrophoresis 30 (Suppl. 1), S162–S173. 10.1002/elps.200900140 19517507

[B38] Guezane-Lakoud S. Ferrah M. Merabet-Khelassi M. Touil N. Toffano M. Aribi-Zouioueche L. (2023). 2-Hydroxymethyl-18-crown-6 as an efficient organocatalyst for alpha-aminophosphonates synthesized under eco-friendly conditions, DFT, molecular docking and ADME/T studies. J. Biomol. Struct. Dyn., 1–17. 10.1080/07391102.2023.2213336 37184142

[B39] Halgren T. A. Murphy R. B. Friesner R. A. Beard H. S. Frye L. L. Pollard W. T. (2004). Glide: a new approach for rapid, accurate docking and scoring. 2. Enrichment factors in database screening. J. Med. Chem. 47 (7), 1750–1759. 10.1021/jm030644s 15027866

[B40] Hameed P. S. Raichurkar A. Madhavapeddi P. Menasinakai S. Sharma S. Kaur P. (2014). Benzimidazoles: novel mycobacterial gyrase inhibitors from scaffold morphing. ACS Med. Chem. Lett. 5 (7), 820–825. 10.1021/ml5001728 25050172 PMC4094266

[B41] Han J. Liu X. Zhang L. Quinn R. J. Feng Y. (2022). Anti-mycobacterial natural products and mechanisms of action. Nat. Prod. Rep. 39 (1), 77–89. 10.1039/d1np00011j 34226909

[B42] Islam M. A. Pillay T. S. (2017). Identification of promising DNA GyrB inhibitors for Tuberculosis using pharmacophore-based virtual screening, molecular docking and molecular dynamics studies. Chem. Biol. Drug Des. 90 (2), 282–296. 10.1111/cbdd.12949 28109130

[B43] Ivanczi M. Balogh B. Kis L. Mandity I. (2023). Molecular dynamics simulations of drug-conjugated cell-penetrating peptides. Pharm. (Basel) 16 (9), 1251. 10.3390/ph16091251 PMC10535489 37765059

[B44] Jacobson M. P. Pincus D. L. Rapp C. S. Day T. J. Honig B. Shaw D. E. (2004). A hierarchical approach to all‐atom protein loop prediction. Proteins Struct. Funct. Bioinforma. 55 (2), 351–367. 10.1002/prot.10613 15048827

[B45] Jagatap V. R. Ahmad I. Sriram D. Kumari J. Adu D. K. Ike B. W. (2023). Isoflavonoid and furanochromone natural products as potential DNA gyrase inhibitors: computational, spectral, and antimycobacterial studies. ACS omega 8 (18), 16228–16240. 10.1021/acsomega.3c00684 37179626 PMC10173323

[B46] Jeankumar V. U. Kotagiri S. Janupally R. Suryadevara P. Sridevi J. P. Medishetti R. (2015). Exploring the gyrase ATPase domain for tailoring newer anti-tubercular drugs: hit to lead optimization of a novel class of thiazole inhibitors. Bioorg Med. Chem. 23 (3), 588–601. 10.1016/j.bmc.2014.12.001 25541204

[B47] Jeankumar V. U. Renuka J. Pulla V. K. Soni V. Sridevi J. P. Suryadevara P. (2014). Development of novel N-linked aminopiperidine-based mycobacterial DNA gyrase B inhibitors: scaffold hopping from known antibacterial leads. Int. J. Antimicrob. Agents 43 (3), 269–278. 10.1016/j.ijantimicag.2013.12.006 24434114

[B48] Jeankumar V. U. Renuka J. Santosh P. Soni V. Sridevi J. P. Suryadevara P. (2013). Thiazole-aminopiperidine hybrid analogues: design and synthesis of novel *Mycobacterium tuberculosis* GyrB inhibitors. Eur. J. Med. Chem. 70, 143–153. 10.1016/j.ejmech.2013.09.025 24148991

[B49] Kale M. G. Raichurkar A. P S. H. Waterson D. McKinney D. Manjunatha M. R. (2013). Thiazolopyridine ureas as novel antitubercular agents acting through inhibition of DNA Gyrase B. J. Med. Chem. 56 (21), 8834–8848. 10.1021/jm401268f 24088190

[B50] Kale R. R. Kale M. G. Waterson D. Raichurkar A. Hameed S. P. Manjunatha M. R. (2014). Thiazolopyridone ureas as DNA gyrase B inhibitors: optimization of antitubercular activity and efficacy. Bioorg Med. Chem. Lett. 24 (3), 870–879. 10.1016/j.bmcl.2013.12.080 24405701

[B51] Kashyap A. Singh P. K. Silakari O. (2018). Chemical classes targeting energy supplying GyrB domain of *Mycobacterium tuberculosis* . Tuberc. (Edinb) 113, 43–54. 10.1016/j.tube.2018.09.001 30514513

[B52] Kawaguchi H. Tsukiura H. Tomita K. Konishi M. Saito K. (1977). Tallysomycin, a new antitumor antibiotic complex related to bleomycin. I. Production, isolation and properties. J Antibiot (Tokyo). 30(10):779–88. 10.7164/antibiotics.30.779 591443

[B53] Khan M. A. Singh S. K. (2023). Atom-based 3D-QSAR and DFT analysis of 5-substituted 2-acylaminothiazole derivatives as HIV-1 latency-reversing agents. J. Biomol. Struct. Dyn. 41 (14), 6759–6774. 10.1080/07391102.2022.2112078 35971967

[B54] Kim Y.-P. Takamatsu S. Hayashi M. Komiyama K. Omura S. (1997). Pyridindolols K1 and K2, new alkaloids from Streptomyces sp. K93-0711. J. antibiotics 50 (3), 189–193. 10.7164/antibiotics.50.189 9439688

[B55] Klyshko E. Kim J. S. McGough L. Valeeva V. Lee E. Ranganathan R. (2024). Functional protein dynamics in a crystal. Nat. Commun. 15 (1), 3244. 10.1038/s41467-024-47473-4 38622111 PMC11018856

[B56] Kumar A. Prasun C. Rathi E. Nair M. S. Kini S. G. (2023). Identification of potential DNA gyrase inhibitors: virtual screening, extra-precision docking and molecular dynamics simulation study. Chem. Pap. 77 (11), 6717–6727. 10.1007/s11696-023-02971-5

[B57] Lamothe S. M. Guo J. Li W. Yang T. Zhang S. (2016). The human ether-a-go-go-related gene (hERG) potassium channel represents an unusual target for protease-mediated damage. J. Biol. Chem. 291 (39), 20387–20401. 10.1074/jbc.M116.743138 27502273 PMC5034037

[B58] LaPointe S. M. Weaver D. F. (2007). A review of density functional theory quantum mechanics as applied to pharmaceutically relevant systems. Curr. Computer-Aided Drug Des. 3 (4), 290–296. 10.2174/157340907782799390

[B59] Lazim R. Suh D. Choi S. (2020). Advances in molecular dynamics simulations and enhanced sampling methods for the study of protein systems. Int. J. Mol. Sci. 21 (17), 6339. 10.3390/ijms21176339 32882859 PMC7504087

[B60] Locher C. P. Jones S. M. Hanzelka B. L. Perola E. Shoen C. M. Cynamon M. H. (2015). A novel inhibitor of gyrase B is a potent drug candidate for treatment of tuberculosis and nontuberculosis mycobacterial infections. Antimicrob. Agents Chemother. 59 (3), 1455–1465. 10.1128/AAC.04347-14 25534737 PMC4325822

[B61] Madhavi Sastry G. Adzhigirey M. Day T. Annabhimoju R. Sherman W. (2013). Protein and ligand preparation: parameters, protocols, and influence on virtual screening enrichments. J. computer-aided Mol. Des. 27, 221–234. 10.1007/s10822-013-9644-8 23579614

[B62] Manathunga M. Götz A. W. Merz Jr K. M. (2022). Computer-aided drug design, quantum-mechanical methods for biological problems. Curr. Opin. Struct. Biol. 75, 102417. 10.1016/j.sbi.2022.102417 35779437

[B63] Medapi B. Renuka J. Saxena S. Sridevi J. P. Medishetti R. Kulkarni P. (2015a). Design and synthesis of novel quinoline-aminopiperidine hybrid analogues as *Mycobacterium tuberculosis* DNA gyraseB inhibitors. Bioorg Med. Chem. 23 (9), 2062–2078. 10.1016/j.bmc.2015.03.004 25801151

[B64] Medapi B. Suryadevara P. Renuka J. Sridevi J. P. Yogeeswari P. Sriram D. (2015b). 4-Aminoquinoline derivatives as novel *Mycobacterium tuberculosis* GyrB inhibitors: structural optimization, synthesis and biological evaluation. Eur. J. Med. Chem. 103, 1–16. 10.1016/j.ejmech.2015.06.032 26318054

[B65] Miethke M. Marahiel M. A. (2007). Siderophore-based iron acquisition and pathogen control. Microbiol. Mol. Biol. Rev. 71 (3), 413–451. 10.1128/MMBR.00012-07 17804665 PMC2168645

[B66] Miotto P. Zhang Y. Cirillo D. M. Yam W. C. (2018). Drug resistance mechanisms and drug susceptibility testing for tuberculosis. Respirology 23 (12), 1098–1113. 10.1111/resp.13393 30189463

[B67] Mohamed M. A. Elsaman T. Elderdery A. Y. Alsrhani A. Ghanem H. B. Alruwaili M. M. (2024). Unveiling the anticancer potential: computational exploration of nitrogenated derivatives of (+)-Pancratistatin as topoisomerase I inhibitors. Int. J. Mol. Sci. 25 (19), 10779. 10.3390/ijms251910779 39409108 PMC11476810

[B68] Mohankumar T. Chandramohan V. Lalithamba H. S. Jayaraj R. L. Kumaradhas P. Sivanandam M. (2020). Design and molecular dynamic investigations of 7,8-dihydroxyflavone derivatives as potential neuroprotective agents against alpha-synuclein. Sci. Rep. 10 (1), 599. 10.1038/s41598-020-57417-9 31953434 PMC6969171

[B69] Murumkar P. R. Sharma M. K. Gupta P. Patel N. M. Yadav M. R. (2023). Selection of suitable protein structure from protein Data Bank: an important step in structure-based drug design studies. Mini Rev. Med. Chem. 23 (3), 246–264. 10.2174/1389557522666220512151454 35549880

[B70] Mustyala K. K. Malkhed V. Chittireddy V. R. Vuruputuri U. (2015). Virtual screening studies to identify novel inhibitors for Sigma F protein of *Mycobacterium tuberculosis* . Int. J. Mycobacteriol 4 (4), 330–336. 10.1016/j.ijmyco.2015.05.013 26964817

[B71] Naz S. Farooq U. Ali S. Sarwar R. Khan S. Abagyan R. (2019). Identification of new benzamide inhibitor against α-subunit of tryptophan synthase from *Mycobacterium tuberculosis* through structure-based virtual screening, anti-tuberculosis activity and molecular dynamics simulations. J. Biomol. Struct. Dyn. 37 (4), 1043–1053. 10.1080/07391102.2018.1448303 29502488

[B72] Ntie-Kang F. (2013). An *in silico* evaluation of the ADMET profile of the StreptomeDB database. Springerplus 2, 353. 10.1186/2193-1801-2-353 23961417 PMC3736076

[B73] Owoloye A. J. Ligali F. C. Enejoh O. A. Musa A. Z. Aina O. Idowu E. T. (2022). Molecular docking, simulation and binding free energy analysis of small molecules as Pf HT1 inhibitors. PloS one 17 (8), e0268269. 10.1371/journal.pone.0268269 36026508 PMC9417013

[B74] Pakamwong B. Thongdee P. Kamsri B. Phusi N. Kamsri P. Punkvang A. (2022). Identification of potent DNA gyrase inhibitors active against *Mycobacterium tuberculosis* . J. Chem. Inf. Model 62 (7), 1680–1690. 10.1021/acs.jcim.1c01390 35347987

[B75] Pakamwong B. Thongdee P. Kamsri B. Phusi N. Taveepanich S. Chayajarus K. (2024). Ligand-based virtual screening for discovery of indole derivatives as potent DNA gyrase ATPase inhibitors active against *Mycobacterium tuberculosis* and hit validation by biological assays. J. Chem. Inf. Model 64 (15), 5991–6002. 10.1021/acs.jcim.4c00511 38993154 PMC11323271

[B76] Pandey R. K. Kumbhar B. V. Srivastava S. Malik R. Sundar S. Kunwar A. (2017). Febrifugine analogues as Leishmania donovani trypanothione reductase inhibitors: binding energy analysis assisted by molecular docking, ADMET and molecular dynamics simulation. J. Biomol. Struct. Dyn. 35 (1), 141–158. 10.1080/07391102.2015.1135298 27043972

[B77] Papillon J. Menetret J. F. Batisse C. Helye R. Schultz P. Potier N. (2013). Structural insight into negative DNA supercoiling by DNA gyrase, a bacterial type 2A DNA topoisomerase. Nucleic Acids Res. 41 (16), 7815–7827. 10.1093/nar/gkt560 23804759 PMC3763546

[B78] Pedrolli D. B. Jankowitsch F. Schwarz J. Langer S. Nakanishi S. Mack M. (2014). “Natural Riboflavin analogs,” in Flavins and flavoproteins: methods and protocols. Editors Weber S. Schleicher E. (New York, NY: Springer New York), 41–63. 10.1007/978-1-4939-0452-5_3 24764087

[B79] Perez-Regidor L. Guzman-Caldentey J. Oberhauser N. Punzon C. Balogh B. Pedro J. R. (2022). Small molecules as toll-like receptor 4 modulators drug and in-house computational repurposing. Biomedicines 10 (9), 2326. 10.3390/biomedicines10092326 36140427 PMC9496124

[B80] Qiu X. Zhang Q. Li Z. Zhang J. Liu H. (2024). Revealing the interaction mechanism between *Mycobacterium tuberculosis* GyrB and novobiocin, SPR719 through binding thermodynamics and dissociation kinetics analysis. Int. J. Mol. Sci. 25 (7), 3764. 10.3390/ijms25073764 38612573 PMC11011931

[B81] Qun T. Zhou T. Hao J. Wang C. Zhang K. Xu J. (2023). Antibacterial activities of anthraquinones: structure-activity relationships and action mechanisms. RSC Med. Chem. 14 (8), 1446–1471. 10.1039/d3md00116d 37593578 PMC10429894

[B82] Ravikumar Y. Koonyosying P. Srichairatanakool S. Ponpandian L. N. Kumaravelu J. Srichairatanakool S. (2023). *In silico* molecular docking and dynamics simulation analysis of potential histone lysine methyl transferase inhibitors for managing β-thalassemia. Molecules 28 (21), 7266. 10.3390/molecules28217266 37959685 PMC10650625

[B83] Reddy K. I. Srihari K. Renuka J. Sree K. S. Chuppala A. Jeankumar V. U. (2014). An efficient synthesis and biological screening of benzofuran and benzo[d]isothiazole derivatives for *Mycobacterium tuberculosis* DNA GyrB inhibition. Bioorg Med. Chem. 22 (23), 6552–6563. 10.1016/j.bmc.2014.10.016 25456076

[B86] Renuka J. Reddy K. I. Srihari K. Jeankumar V. U. Shravan M. Sridevi J. P. (2014). Design, synthesis, biological evaluation of substituted benzofurans as DNA gyraseB inhibitors of *Mycobacterium tuberculosis* . Bioorg Med. Chem. 22 (17), 4924–4934. 10.1016/j.bmc.2014.06.041 25129171

[B87] Rukachaisirikul T. Saekee A. Tharibun C. Watkuolham S. Watkuolham S. Suksamrarn A. (2007). Biological activities of the chemical constituents of Erythrina stricta and Erythrina subumbrans. Arch. Pharm. Res. 30 (11), 1398–1403. 10.1007/BF02977363 18087807

[B88] Salo-Ahen O. M. H. Alanko I. Bhadane R. Bonvin A. M. J. J. Honorato R. V. Hossain S. (2020). Molecular dynamics simulations in drug discovery and pharmaceutical development. Processes 9 (1), 71. 10.3390/pr9010071

[B89] Sanz M. Salinas R. K. Pinto E. (2017). Namalides B and C and spumigins K-N from the cultured freshwater cyanobacterium sphaerospermopsis torques-reginae. J. Nat. Prod. 80 (9), 2492–2501. 10.1021/acs.jnatprod.7b00370 28876933

[B90] Saxena S. Samala G. Renuka J. Sridevi J. P. Yogeeswari P. Sriram D. (2015). Development of 2-amino-5-phenylthiophene-3-carboxamide derivatives as novel inhibitors of *Mycobacterium tuberculosis* DNA GyrB domain. Bioorg Med. Chem. 23 (7), 1402–1412. 10.1016/j.bmc.2015.02.032 25766629

[B190] Schrödinger LLC. (2023a). LigPrep (Release 2023-1). New York, NY: Schrödinger, LLC.

[B191] Schrödinger LLC. (2023b). Maestro (Release 2023-1). New York, NY: Schrödinger, LLC.

[B91] Seung K. J. Keshavjee S. Rich M. L. (2015). Multidrug-resistant tuberculosis and extensively drug-resistant tuberculosis. Cold Spring Harb. Perspect. Med. 5 (9), a017863. 10.1101/cshperspect.a017863 25918181 PMC4561400

[B92] Shirude P. S. Madhavapeddi P. Tucker J. A. Murugan K. Patil V. Basavarajappa H. (2013). Aminopyrazinamides: novel and specific GyrB inhibitors that kill replicating and nonreplicating *Mycobacterium tuberculosis* . ACS Chem. Biol. 8 (3), 519–523. 10.1021/cb300510w 23268609

[B93] Srivastava A. K. Misra N. (2021). DFT-based studies on bioactive molecules. Sharjah, United Arab Emirates: Bentham Science Publishers.

[B94] Stielow M. Witczynska A. Kubryn N. Fijalkowski L. Nowaczyk J. Nowaczyk A. (2023). The bioavailability of drugs-the current state of knowledge. Molecules 28 (24), 8038. 10.3390/molecules28248038 38138529 PMC10745386

[B95] Sudarshan K. Yarlagadda S. Sengupta S. (2024). Recent advances in the synthesis of diarylheptanoids. Chem. Asian J. 19 (15), e202400380. 10.1002/asia.202400380 38744677

[B96] Szabo T. Volk B. Milen M. (2021). Recent advances in the synthesis of β-carboline alkaloids. Molecules 26 (3), 663. 10.3390/molecules26030663 33513936 PMC7866041

[B97] Takahashi H. Isobe M. Goto T. (1991). Chemical synthesis of lampteroflavin as light emitter in the luminous mushroom, Lampteromyces japonicus. Tetrahedron 47 (32), 6215–6222. 10.1016/s0040-4020(01)86553-4

[B98] Talley A. K. Thurston A. Moore G. Gupta V. K. Satterfield M. Manyak E. (2021). First-in-human evaluation of the safety, tolerability, and pharmacokinetics of SPR720, a novel oral bacterial DNA gyrase (GyrB) inhibitor for mycobacterial infections. Antimicrob. Agents Chemother. 65 (11), e0120821. 10.1128/aac.01208-21 34491803 PMC8525492

[B99] Tambe P. M. Bhowmick S. Chaudhary S. K. Khan M. R. Wabaidur S. M. Muddassir M. (2020). Structure-based screening of DNA GyraseB inhibitors for therapeutic applications in tuberculosis: a pharmacoinformatics study. Appl. Biochem. Biotechnol. 192 (4), 1107–1123. 10.1007/s12010-020-03374-y 32686004

[B100] Thuan N. H. Tatipamula V. B. Canh N. X. Van Giang N. (2022). Recent advances in microbial co-culture for production of value-added compounds. 3 Biotech. 12 (5), 115. 10.1007/s13205-022-03177-4 PMC9018925 35547018

[B101] Tomasic T. Zubriene A. Skok Z. Martini R. Pajk S. Sosic I. (2021). Selective DNA gyrase inhibitors: multi-target *in silico* profiling with 3D-pharmacophores. Pharm. (Basel) 14 (8), 789. 10.3390/ph14080789 PMC8400042 34451886

[B102] Trenado-Uribe M. Silva-Miranda M. Rivero-Cruz J. F. Rodríguez-Peña K. Espitia-Pinzón C. I. Rodríguez-Sanoja R. (2018). Antimycobacterial activity of an anthracycline produced by an endophyte isolated from Amphipterygium adstringens. Mol. Biol. Rep. 45 (6), 2563–2570. 10.1007/s11033-018-4424-0 30311126

[B103] Verma H. Nagar S. Vohra S. Pandey S. Lal D. Negi R. K. (2021). Genome analyses of 174 strains of *Mycobacterium tuberculosis* provide insight into the evolution of drug resistance and reveal potential drug targets. Microb. Genom 7 (3), mgen000542. 10.1099/mgen.0.000542 33750515 PMC8190606

[B104] Voet A. Qing X. Lee X. Y. De Raeymaecker J. Tame J. Zhang K. (2014). Pharmacophore modeling: advances, limitations, and current utility in drug discovery. J. Recept. Ligand Channel Res., 81. 10.2147/jrlcr.S46843

[B105] Wang J.-L. Sang C.-Y. Wang J. Li P.-L. Chai T. Naghavi M. R. (2023). Sesquiterpene coumarins from Ferula sinkiangensis and their anti-pancreatic cancer effects. Phytochemistry 214, 113824. 10.1016/j.phytochem.2023.113824 37597719

[B106] Wang X. Song K. Li L. Chen L. (2018). Structure-based drug design strategies and challenges. Curr. Top. Med. Chem. 18 (12), 998–1006. 10.2174/1568026618666180813152921 30101712

[B107] Xiong G. Wu Z. Yi J. Fu L. Yang Z. Hsieh C. (2021). ADMETlab 2.0: an integrated online platform for accurate and comprehensive predictions of ADMET properties. Nucleic Acids Res. 49 (W1), W5–W14. 10.1093/nar/gkab255 33893803 PMC8262709

[B108] Yele V. Sigalapalli D. K. Jupudi S. Mohammed A. A. (2021). DFT calculation, molecular docking, and molecular dynamics simulation study on substituted phenylacetamide and benzohydrazide derivatives. J. Mol. Model 27 (12), 359. 10.1007/s00894-021-04987-8 34816313

[B109] Yu W. MacKerell A. D. Jr. (2017). Computer-aided drug design methods. Methods Mol. Biol. 1520, 85–106. 10.1007/978-1-4939-6634-9_5 27873247 PMC5248982

[B110] Zhang Z. Tang W. (2018). Drug metabolism in drug discovery and development. Acta Pharm. Sin. B 8 (5), 721–732. 10.1016/j.apsb.2018.04.003 30245961 PMC6146880

[B111] Zhu Y. Ouyang Z. Du H. Wang M. Wang J. Sun H. (2022). New opportunities and challenges of natural products research: when target identification meets single-cell multiomics. Acta Pharm. Sin. B 12 (11), 4011–4039. 10.1016/j.apsb.2022.08.022 36386472 PMC9643300

