# Supplementary material for: Microbial-based natural products as potential inhibitors targeting DNA gyrase B of Mycobacterium tuberculosis: an in silico study
Source: Front Chem. 2025 Jan 23;13:1524607. doi: 10.3389/fchem.2025.1524607 (PMC11798933; doi:10.3389/fchem.2025.1524607)
Supplement: Supplementary file 1 [file DataSheet1.docx]

Supplementary Material

**Microbial-based natural products as potential inhibitors targeting DNA gyrase B of *Mycobacterium tuberculosis*: An *in silico* study**

**Tilal Elsaman*^1^, Magdi Awadalla Mohamed*^1^, Malik Suliman Mohamed^2^, Eyman Mohamed Eltayib^2^, Abualgasim Elgaili Abdalla^3^**

^1^Department of Pharmaceutical Chemistry, College of Pharmacy, Jouf University, Sakaka, Kingdom of Saudi Arabia

^2^Department of Pharmaceutics, College of Pharmacy, Jouf University, Sakaka, Kingdom of Saudi Arabia

^3^Department of Clinical Laboratory Sciences, College of Applied Medical Sciences, Jouf University, Sakaka, Saudi Arabia

*Author to whom correspondence should be addressed; telbashir@ju.edu.sa and maelhussein@ju.edu.sa

**Supplementary Table S1. Physicochemical and pharmacokinetics descriptors calculated with QikProp**

| **Property** | Permissible range | SPR720 | Glysperin B | Dipleosporalone B | Fuscachelin A | 1-Hydroxy-D-788-7 | Wexrubicin | Pyridindolol K2 | Pyrronamycin B | Closthioamide | Erythrin | Lampteroflavin | NPA007770 | Dihydrospumigin N |
| --- | --- | --- | --- | --- | --- | --- | --- | --- | --- | --- | --- | --- | --- | --- |
| #stars | (0–5) | 0 | 18 | 12 | 18 | 4 | 3 | 0 | 12 | 9 | 1 | 6 | 2 | 9 |
| #amide | (0–1) | 1 | 0 | 0 | 6 | 0 | 0 | 0 | 1 | 0 | 0 | 0 | 0 | 3 |
| #rotor | (0–15) | 8 | 32 | 14 | 31 | 11 | 11 | 6 | 19 | 20 | 13 | 14 | 18 | 20 |
| #rtvFG | (0–2) | 0 | 4 | 2 | 2 | 1 | 1 | 1 | 1 | 0 | 2 | 1 | 0 | 0 |
| mol_MW | (130–725) | 506.4 | 919 | 804.8 | 1030 | 531.5 | 540.4 | 300.3 | 555.5 | 695 | 422.3 | 508.4 | 422.5 | 598.6 |
| SASA | (300–1000) | 812.9 | 1239.4 | 1193.6 | 1547 | 7568 | 763.8 | 568.1 | 998.1 | 1169.3 | 692.6 | 777.1 | 768.1 | 921.8 |
| FOSA | (0–750) | 448.6 | 678.1 | 415.6 | 480.9 | 304 | 226.8 | 196.1 | 239.7 | 347.9 | 229.2 | 345.3 | 408.8 | 335.1 |
| FISA | (7–330) | 223.9 | 440.6 | 459.9 | 799.9 | 322.4 | 368.1 | 156.8 | 571.4 | 172.6 | 311.2 | 361 | 267 | 342.5 |
| PISA | (0–450) | 121.2 | 120.7 | 318.0 | 266 | 130.3 | 150.8 | 215 | 186.9 | 308.9 | 152.2 | 70.7 | 92.3 | 244.1 |
| WPSA | (0–175) | 19.0 | 0 | 0 | 0 | 0 | 0 | 0 | 0 | 339.8 | 0 | 0 | 0 | 0 |
| donorHB | (0–6) | 4 | 17 | 0 | 13 | 5 | 5 | 3 | 10.2 | 8 | 4 | 7 | 5 | 9.2 |
| accptHB | (2–20) | 11.2 | 34 | 15 | 25 | 11.5 | 15.5 | 6.4 | 16.9 | 13.5 | 9.8 | 19.1 | 9.3 | 14.1 |
| QPlogPo/w | (−2–6.5) | 2.5 | -7.01 | 3.3 | -4.3 | 0.3 | -0.9 | 1.5 | -3.2 | 5.8 | 0.6 | -2.4 | 1.7 | -0.5 |
| QPPCaco | (<25poor, >500 great) | 3.2 | 0.005 | 0.43 | 0 | 2.1 | 2.1 | 322.1 | 0 | 228.3 | 11 | 3.7. | 29 | 2.7 |
| CIQPlogS | –6.5 – 0.5 | -5.194 | 1.4 | -10.0 | -5.2 | -4.9 | -5 | -3.5 | -3.2 | -7.9 | -4.5 | -2.8 | -4.2 | -3.9 |
| QPlogBB | –3.0 – 1.2 | -2.4 | -6.2 | -6.5 | -14.5 | -3 | -4 | -1.3 | -8 | -2.3 | -3.4 | -4.2 | -3.4 | -4.4 |
| QPPMDCK | <25 poor >500 great | 3.15 | 0.003 | 0.1 | 0 | 0.7 | 0.6 | 145.4 | 0 | 7291.5 | 3.81 | 1.1 | 10.7 | 1.8 |
| #metab | (1 – 8) | 4 | 15 | 14 | 16 | 9 | 8 | 3 | 9 | 6 | 8 | 8 | 8 | 10 |
| QPlogKhsa | −1.5–1.5 | -0.64 | -3.265 | 0.72 | -2.8 | -0.26 | -0.8 | -0.2 | -1.5 | 0.1 | -0.5 | -1.4 | -0.3 | -1.4 |
| QPlogHERG | Concern below –5 | -0.872 | -8.2 | -7.947 | -0.4 | -6 | -5.4 | -5.3 | -6.6 | -8.2 | -5.4 | -5.4 | -5.5 | -2.2 |
| HumanOralAbsorption | 1, 2, or 3 for low, medium, or high. | 2 | 1 | 1 | 1 | 1 | 1 | 3 | 1 | 1 | 2 | 1 | 1 | 1 |
| %Absorption | >80% is high | 24.9 | 0 | 0.9 | 0 | 0 | 0 | 81 | 0 | 64.75 | 36.2 | 0 | 50.1 | 0 |
| PSA | 7–200 | 160.0 | 345.7 | 297.1 | 565.3 | 209.9 | 221.3 | 99.3 | 340.3 | 150.8 | 182.1 | 226.4 | 146.2 | 230.9 |
| Rule Of Five | maximum is 4 | 2 | 3 | 3 | 3 | 3 | 3 | 0 | 3 | 3 | 1 | 3 | 1 | 3 |
| Rule Of Three | maximum is 3 | 1 | 2 | 3 | 2 | 2 | 2 | 0 | 2 | 1 | 2 | 2 | 1 | 2 |

**Supplementary Table S2: Toxicity profile of the top 12 hits with ADMETLab 2.0**

| **Property** | **Description** | Empirical decision | SPR720 | Glysperin B | Dipleosporalone B | Fuscachelin A | 1-Hydroxy-D-788-7 | Wexrubicin | Pyridindolol K2 | Pyrronamycin B | Closthioamide | Erythrin | Lampteroflavin | NPA007770 | Dihydrospumigin N |
| --- | --- | --- | --- | --- | --- | --- | --- | --- | --- | --- | --- | --- | --- | --- | --- |
| H-HT | The human hepatotoxicity | (0-0.3: excellent; 0.3-0.7: medium; 0.7-1.0: poor) | 0.96 | 0.97 | 0.71 | 0.95 | 0.93 | 0.88 | 0.48 | 0.95 | 0.015 | 0.09 | 0.84 | 0.63 | 0.74 |
| DILI | Drug-induced liver injury | (0-0.3: excellent; 0.3-0.7: medium; 0.7-1.0: poor | 1 | 0.92 | 0.87 | 0.92 | 0.97 | 0.99 | 0.53 | 0.89 | 1 | 0.12 | 0.87 | 0.01 | 0.01 |
| AMES Toxicity | test for mutagenicity | (0-0.3: excellent; 0.3-0.7: medium; 0.7-1.0: poor | 0.023 | 0.99 | 0.75 | 0.99 | 0.98 | 0.99 | 0.92 | 0.78 | 0.63 | 0.22 | 0.82 | 0.2 | 0.2 |
| ROA | Determination of oral acute toxicity in rats | (0-0.3: excellent; 0.3-0.7: medium; 0.7-1.0: poor | 0.01 | 0.003 | 0.12 | 0.003 | 0.89 | 0.18 | 0.16 | 0.53 | 0.82 | 0.01 | 0.12 | 0.04 | 0.29 |
| FDAMDD | The maximum recommended daily dose provides an estimate of the toxic dose threshold of chemicals in humans | (0-0.3: excellent; 0.3-0.7: medium; 0.7-1.0: poor | 0.99 | 0.001 | 0.63 | 0.001 | 0.96 | 0.19 | 0.55 | 0.86 | 0.94 | 0.26 | 0.55 | 0.65 | 0.99 |
| SkinSen | Skin Sensitization | (0-0.3: excellent; 0.3-0.7: medium; 0.7-1.0: poor | 0.57 | 1 | 0.99 | 1 | 0.98 | 0.99 | 0.67 | 0.94 | 0.98 | 0.41 | 0.69 | 0.24 | 0.98 |
| Carcinogencity | The ability to damage the genome or disrupt cellular metabolic processes | (0-0.3: excellent; 0.3-0.7: medium; 0.7-1.0: poor | 0.90 | 0.001 | 0.79 | 0.001 | 0.85 | 0.59 | 0.92 | 0.29 | 0.74 | 0.28 | 0.34 | 0.09 | 0.02 |
| EC | Assessing the eye corrosion (EC) potential of a chemical as a necessary component of risk assessment | (0-0.3: excellent; 0.3-0.7: medium; 0.7-1.0: poor | 0 | 0 | 0 | 0 | 0 | 0 | 0 | 0 | 0 | 0 | 0 | 0 | 0 |
| EI | Assessing the eye Irritation (EI) potential of a chemical as a necessary component of risk assessment | (0-0.3: excellent; 0.3-0.7: medium; 0.7-1.0: poor | 0.07 | 0 | 0.02 | 0 | 0.02 | 0.16 | 0.17 | 0 | 0.3 | 0.65 | 0.09 | 0.05 | 0 |
| Respiratory Toxicity | Assessment of the potential of Drug-induced respiratory toxicity | (0-0.3: excellent; 0.3-0.7: medium; 0.7-1.0: poor | 0.97 | 0.005 | 0.103 | 0.005 | 0.93 | 0.27 | 0.07 | 0.24 | 0.12 | 0.07 | 0.2 | 0.4 | 0.12 |

**Supplementary Table S3 Chemical structures and IC_50_ of some reported compounds as *M. tuberculosis* GYR B inhibitors.**

| **NO** | **Chemical structure** | **IC_50_ (µM)** |
| --- | --- | --- |
|  | **** | **1.7** |
|  | **** | **0.002** |
|  | **** | **0.01** |
|  | **** | **0.006** |
|  | **** | **0.02** |
|  | **** | **0.002** |
|  | **** | **0.229** |
|  | **** | **0.28** |
|  | **** | **0.029** |
|  | **** | **0.009** |
|  | **** | **0.022** |
|  | 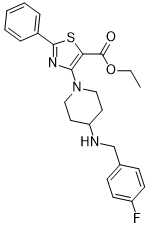 | **96% inhibition concentration 50 µM** |
|  | 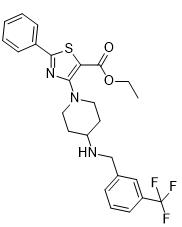 | **96% inhibition concentration 50 µM** |
|  | 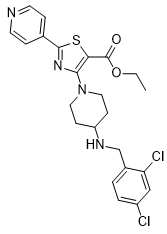 | **92% inhibition concentration 50 µM** |
|  | 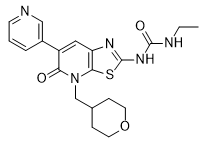 | **0.014** |
|  | 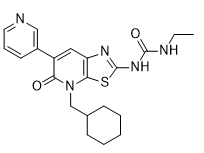 | **0.02** |
|  | 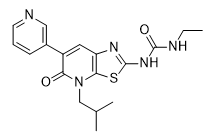 | **0.025** |
|  | 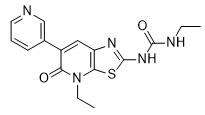 | **0.03** |
|  | 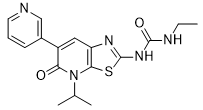 | **0.04** |
|  | 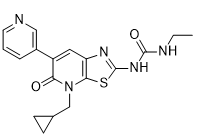 | **0.04** |
|  | 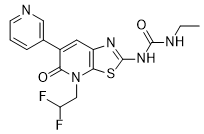 | **0.04** |
|  | 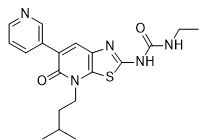 | **0.05** |
|  | 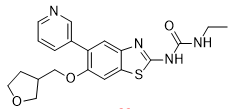 | **0.005** |
|  | 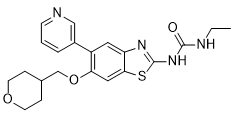 | **0.009** |
|  | 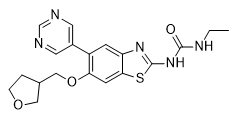 | **0.01** |
|  | 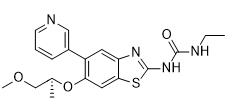 | **0.012** |
|  | 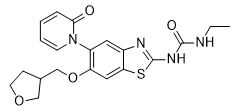 | **0.001** |
|  | 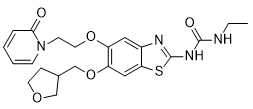 | **0.0005** |
|  | 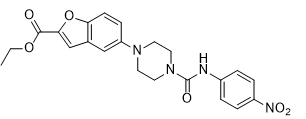 | **5** |
|  | 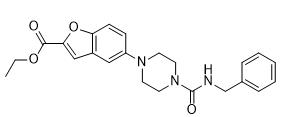 | **4.32** |
|  | 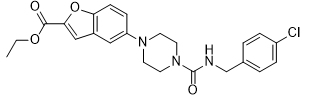 | **2.97** |
|  | 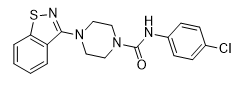 | **1.77** |
|  | 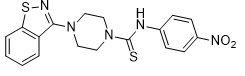 | **1.81** |
|  | 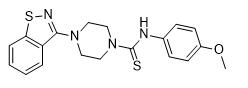 | **2.86** |
|  | 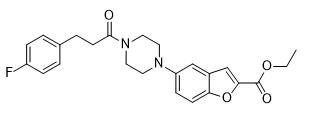 | **6.14** |
|  | 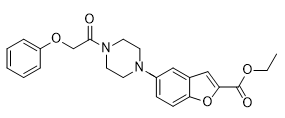 | **3.97** |
|  | 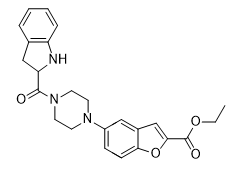 | **3.89** |
|  | 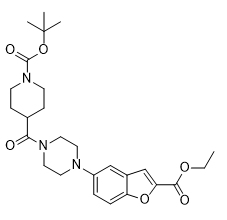 | **3.2** |
|  | 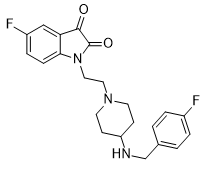 | **20.3** |
|  | 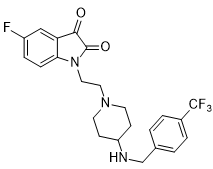 | **10.6** |
|  | 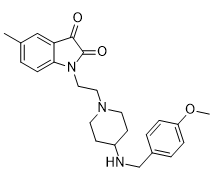 | **10.6** |
|  | 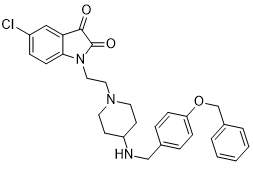 | **13.8** |
|  | 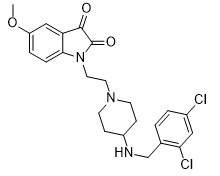 | **15.8** |
|  | 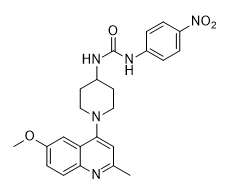 | **6.99** |
|  | 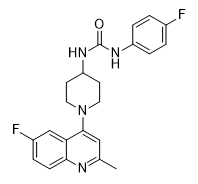 | **1.78** |
|  | 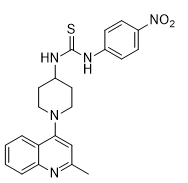 | **3.77** |
|  | 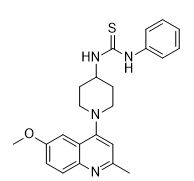 | **2.14** |
|  | 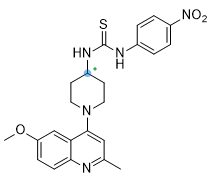 | **2.5** |
|  | 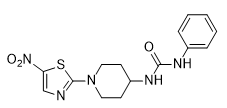 | **1.75** |
|  | 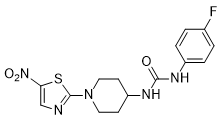 | **2.6** |
|  | 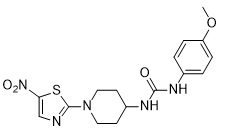 | **3.56** |
|  | 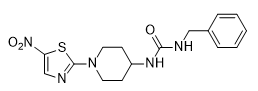 | **2.13** |
|  | 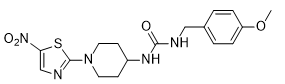 | **1.5** |
|  | 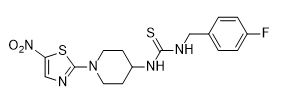 | **1.5** |
|  | 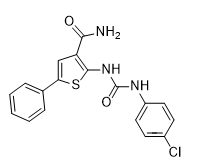 | **3.14** |
|  | 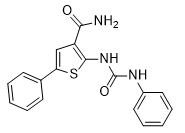 | **1.83** |
|  | 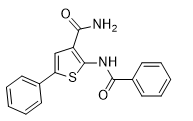 | **0.86** |
|  | 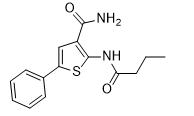 | **3.57** |
|  | 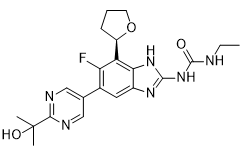 | **ND** |
|  | 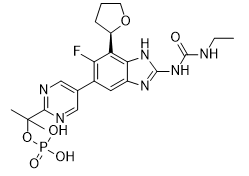 | **ND** |
|  | 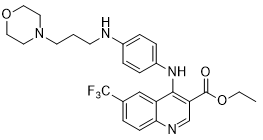 | **6.62** |
|  | 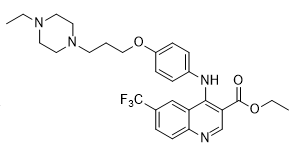 | **0.97** |
|  | 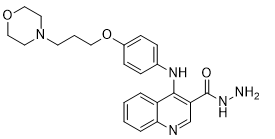 | **0.97** |
|  | 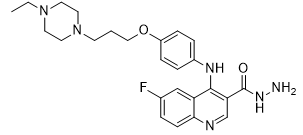 | **2.92** |
|  | 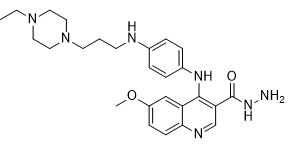 | **3.26** |
|  | 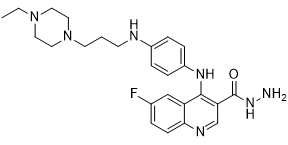 | **1.15** |
|  | 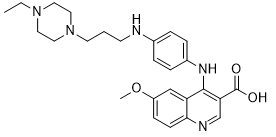 | **1.32** |


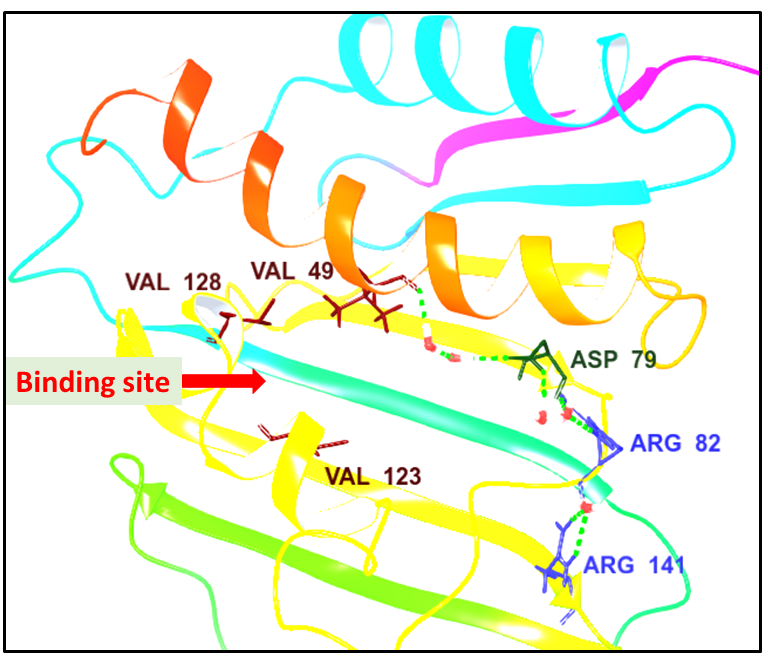
**Supplementary Figure S1. Key molecular interaction sites in mycobacterium tuberculosis DNA gyrase B (PDB ID: 4B6C) highlighting the most critical residues: ASP79, VAL49, VAL123, VAL128, ARG82, and ARG141.**


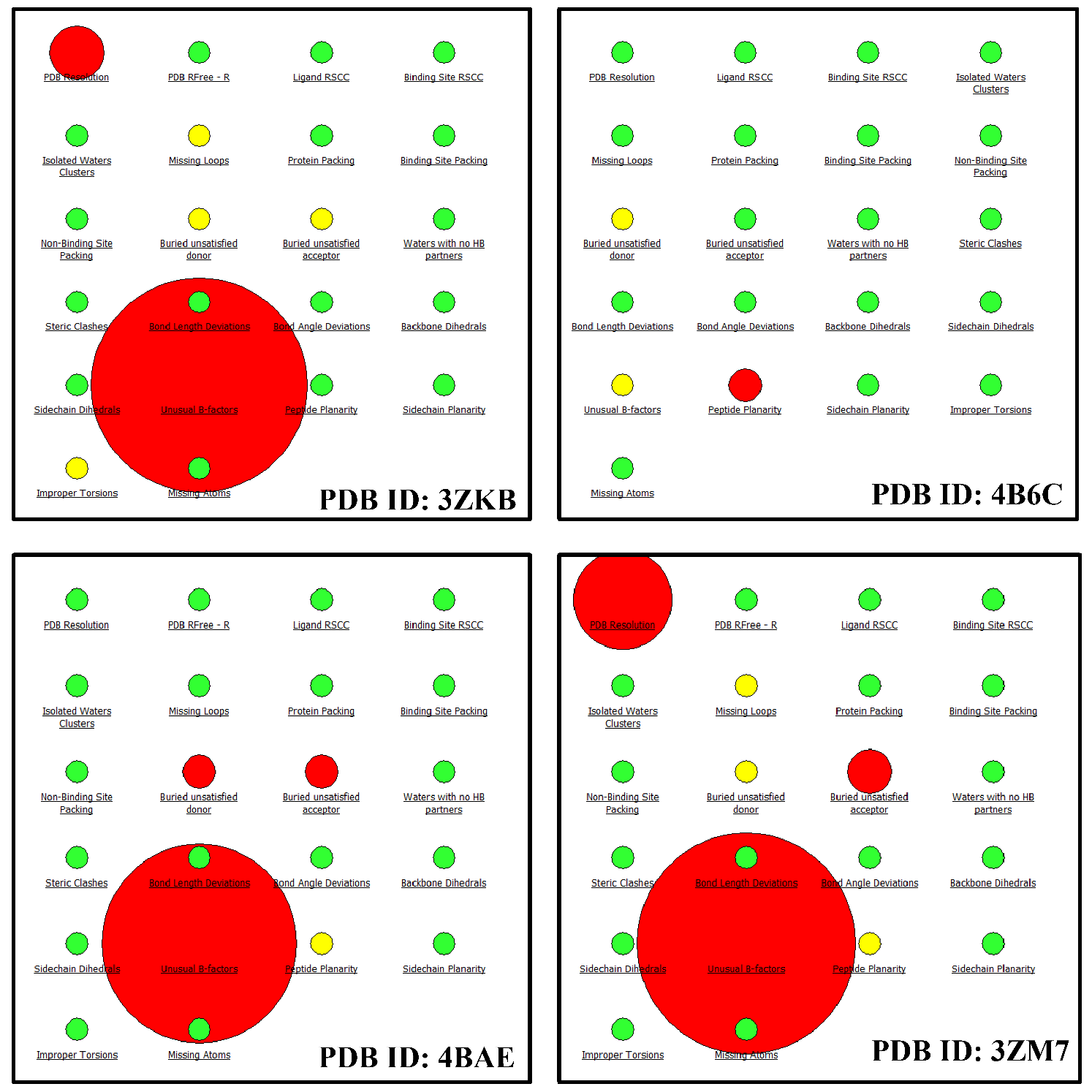


Supplementary Figure S2. Comparison of reliability metrics for the investigated GyrB crystal structures downloaded from Protein Data Bank (PDB).

| 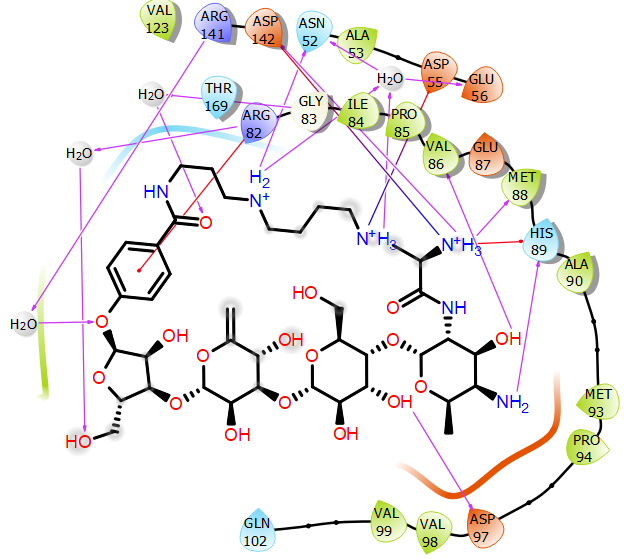 | 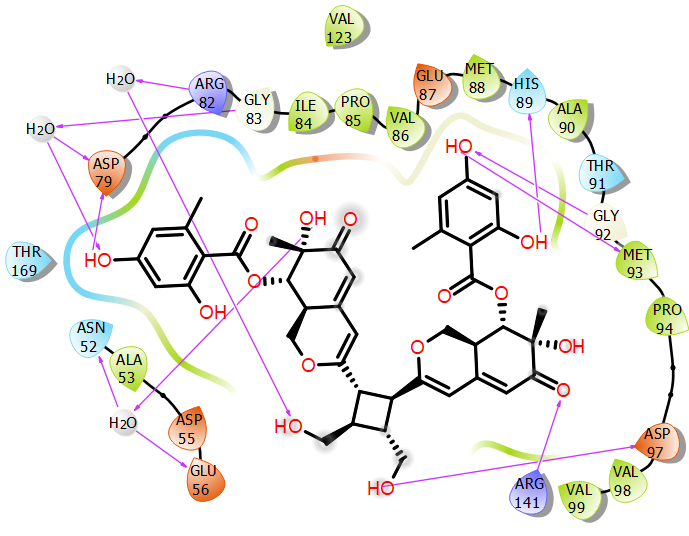 |
| --- | --- |
| **Glysperin B** | **Dipleosporalone B** |
| 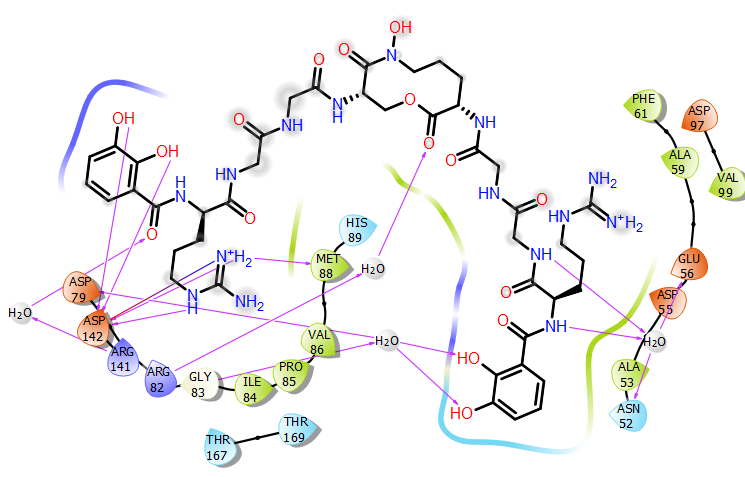 | 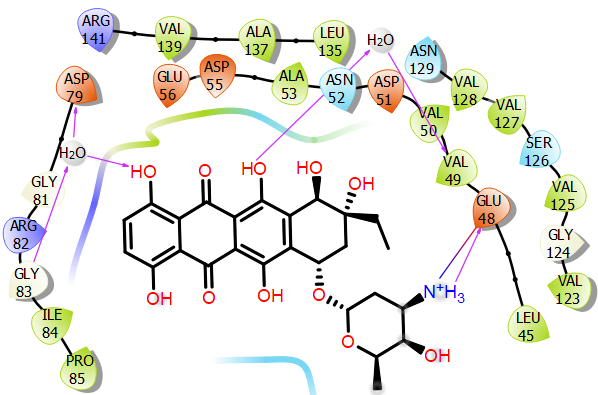 |
| **Fuscachelin A** | **1-Hydroxy-D-788-7** |
| 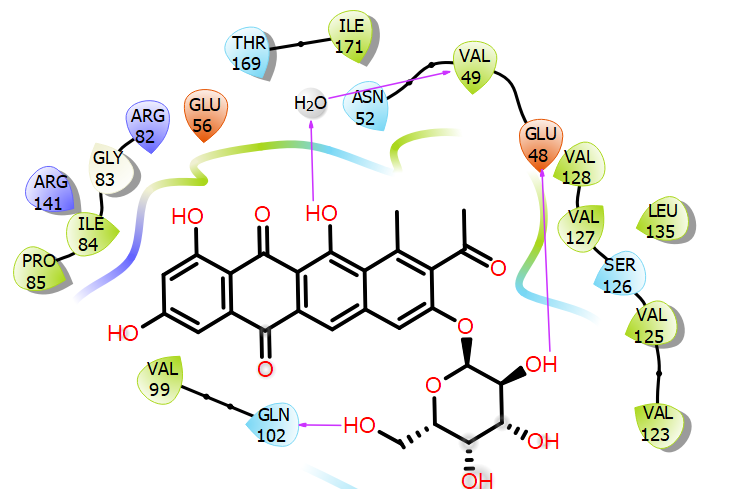 | 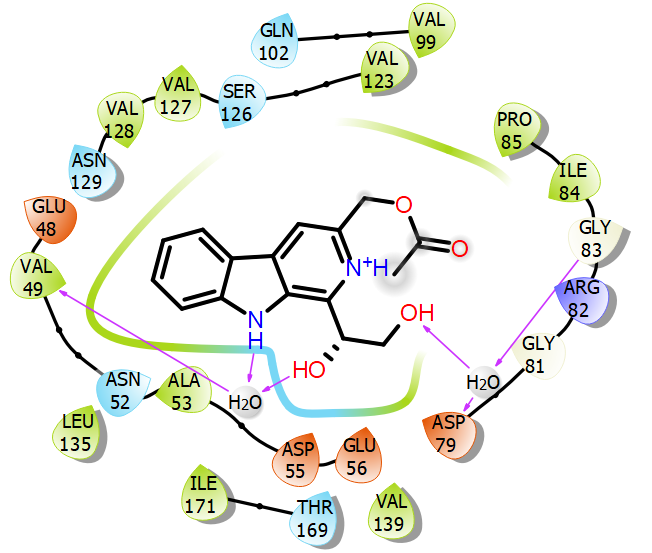 |
| **Wexrubicin** | **Pyridindolol K2** |
| 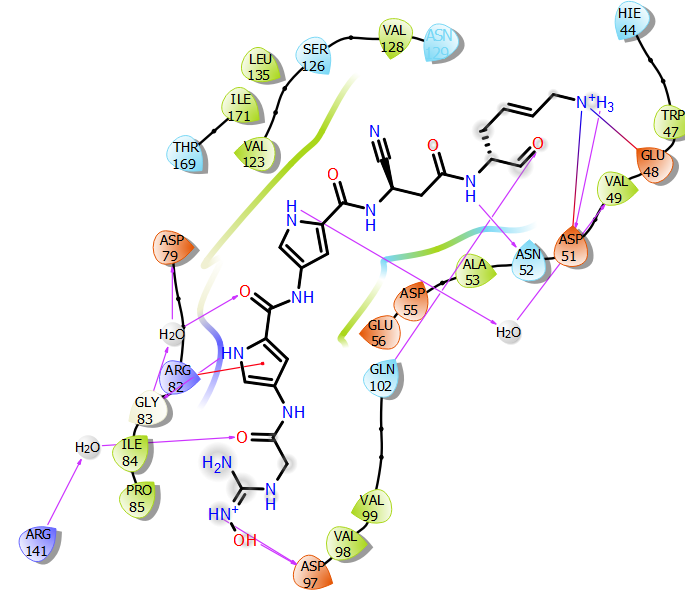 | 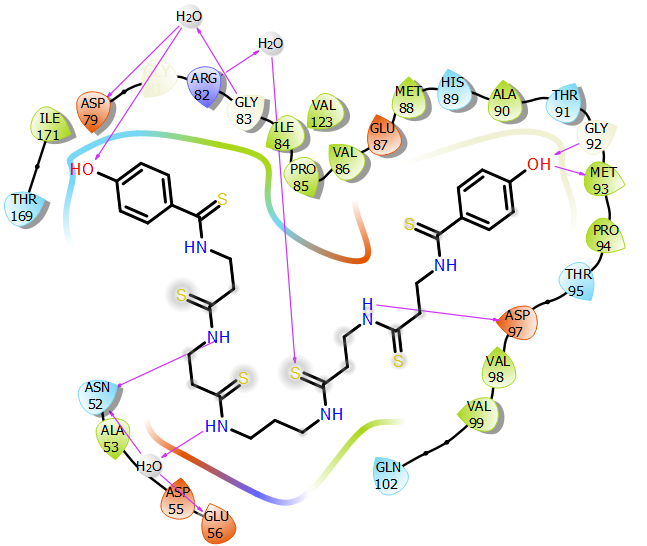 |
| **Pyrronamycin B** | **Closthioamide** |
| 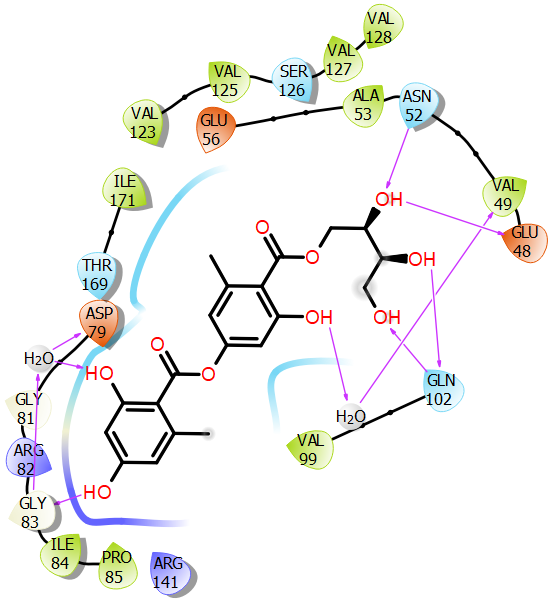 | 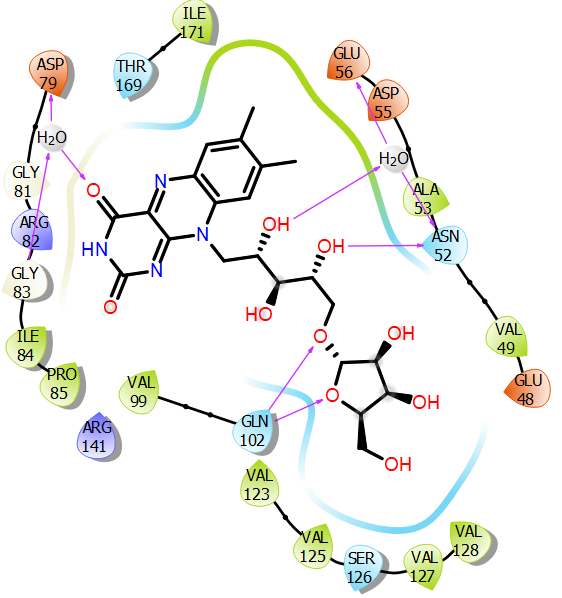 |
| **Erythrin** | **Lampteroflavin** |
| 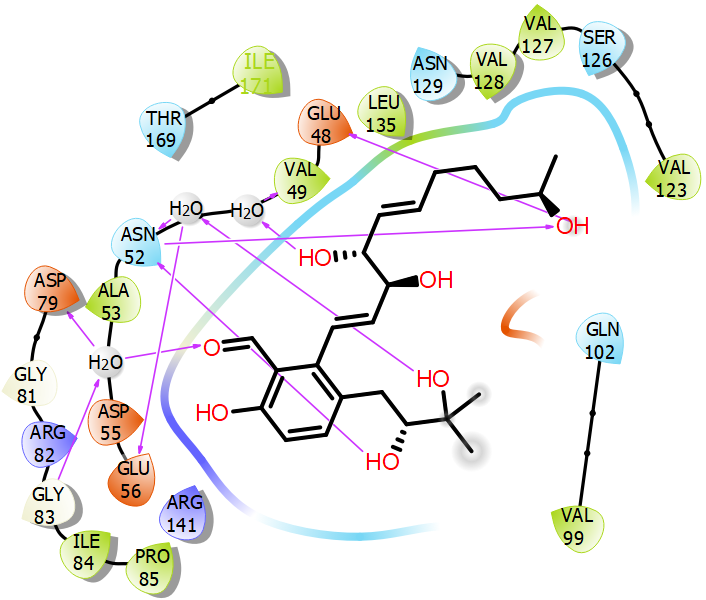 | 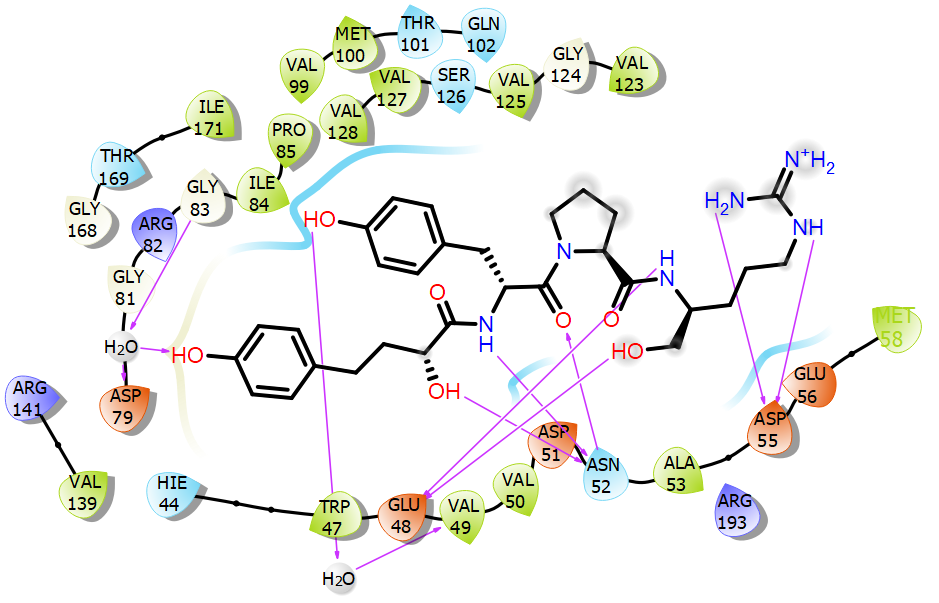 |
| **NPA007770** | **Dihydrospumigin N** |

**Supplementary Figure S3. 2D enzyme-ligand interactions of the top-ranked hits with MBT Gyr B binding site residues. The salt bridges, H-Bonds and Pi-cation interactions are shown by blue, magenta, and red lines, respectively. Created using Maestro interface of Schrödinger suite version 2023-1.**

**
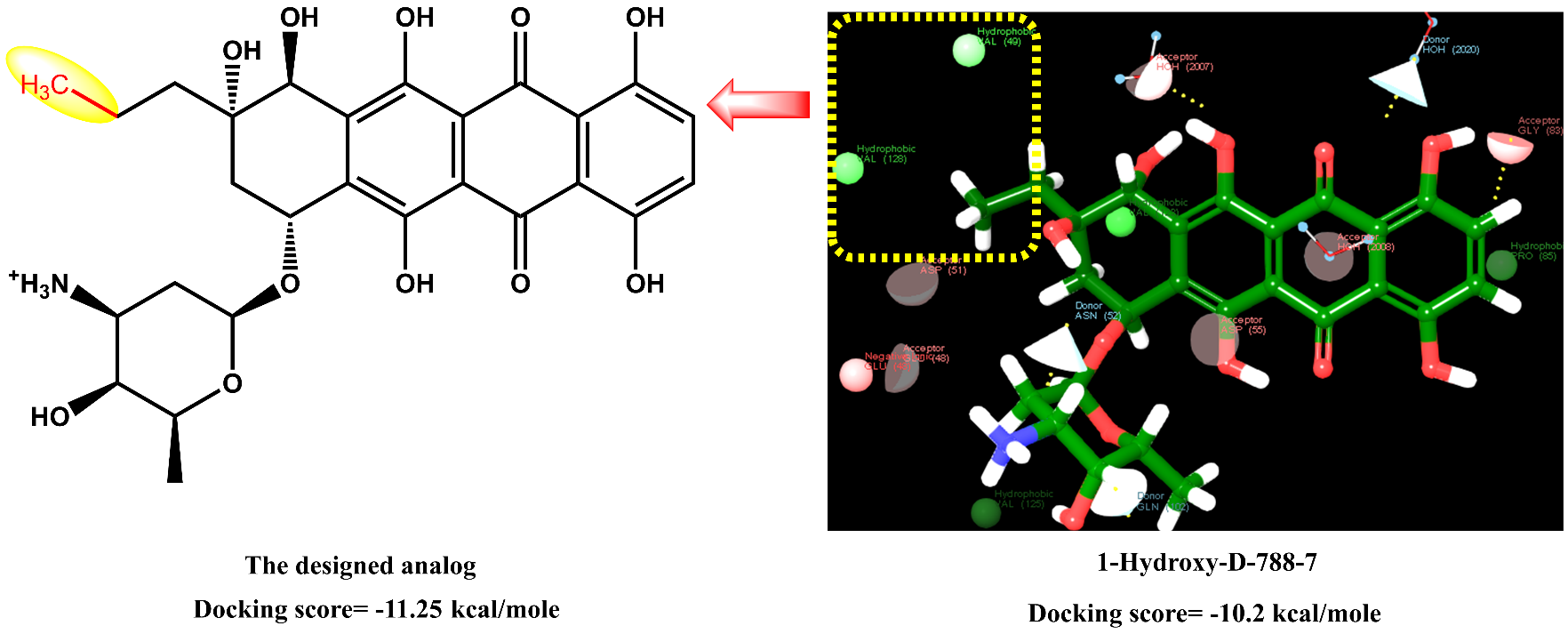
**

**Supplementary Figure S4. Structural comparison of 1-hydroxy-d-788-7 and its propyl-modified analog with highlighted R-group modifications. The extension of the alkyl chain is expected to enhance hydrophobic interactions with the hydrophobic residues Val123, Val125, and Val128, potentially improving binding affinity and specificity.**

| 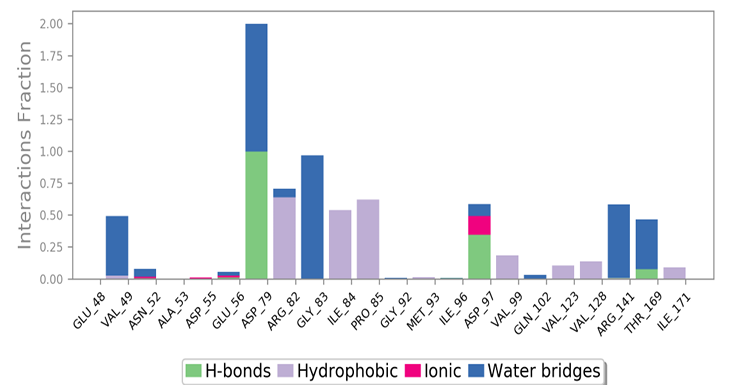 |
| --- |
| **Co-crystal ligand** |
| 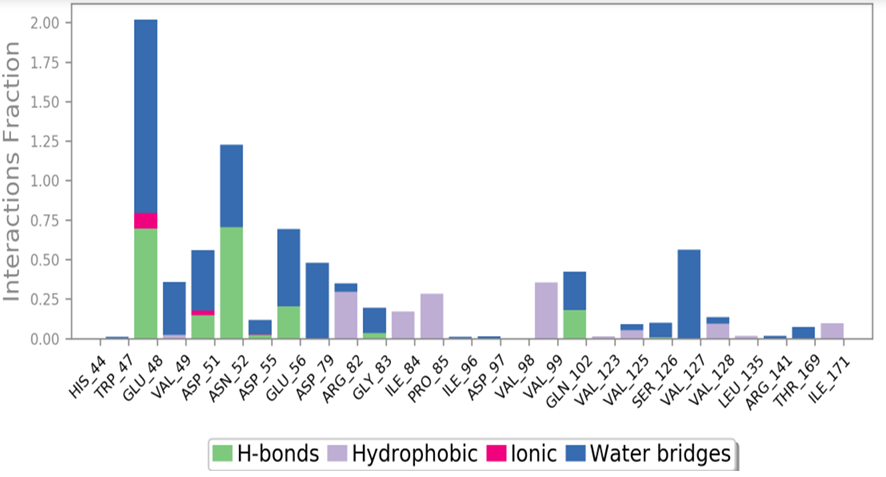 |
| **1-Hydroxy-D-788-7** |
| 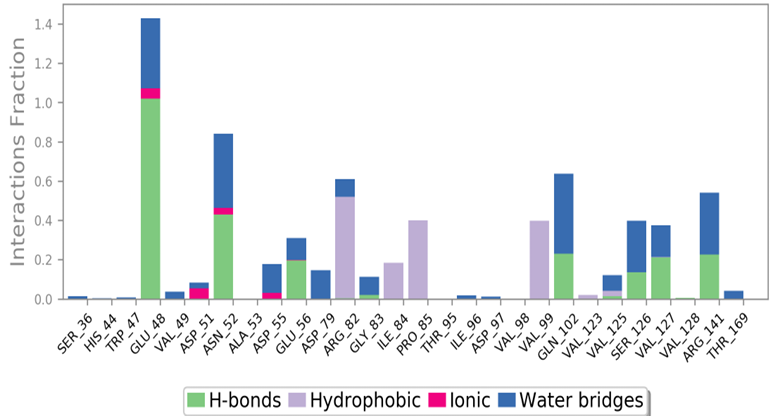 |
| **Erythrin** |
| 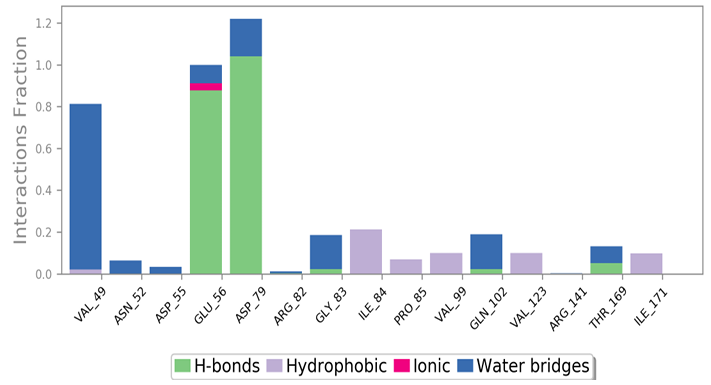 |
| **Pyrindolol K2** |

**Supplementary Figure S5. Protein-Ligand Contacts Histogram Plot for the Co-Crystal Ligand and Top 3 Hits (1-Hydroxy-D-788-7, Erythrin, and Pyrindolol K2) monitored throughout the entire simulation run. The interactions are organized by kind and depicted in a bar diagram.**
